# Protein kinase C-λ drives third-degree burn-induced muscle wasting via STAT3-dependent catabolic pathways

**DOI:** 10.3389/fphar.2026.1839235

**Published:** 2026-09-03

**Authors:** Peilong Li, Xinzhuang Liu, Guobao Huang

**Affiliations:** Department of Burn and Plastic Surgery, Central Hospital Affiliated to Shandong First Medical University, Jinan, Shandong, China

**Keywords:** burn injury, ICA-1, muscle atrophy, protein kinase C-lambda, STAT3 signaling

## Abstract

**Objective:**

Severe burn injury triggers profound skeletal muscle atrophy through incompletely understood molecular mechanisms. This study investigated the role of protein kinase C (PKC) isoforms in burn-induced muscle wasting and evaluated potential therapeutic targets.

**Methods:**

We examined PKC isoform expression in skeletal muscle biopsies from burn patients with second-degree (n = 5) and third-degree burns (n = 5) at 7 days post-injury. Mouse burn models were established to investigate PKC signaling at 14 days post-injury. Muscle-specific PKC-lambda (PKC-λ) knockout mice and pharmacological inhibition with [4-(5-amino-4-carbamoylimidazol-1-yl)-2,3-dihydroxycyclopentyl] methyl dihydrogen phosphate (ICA-1) were employed. C2C12 myotubes were used for mechanistic studies with signal transducer and activator of transcription 3 (STAT3) overexpression experiments.

**Results:**

Among six PKC isoforms examined, only PKC-λ was selectively upregulated in third-degree versus second-degree burns in both humans and mice. Third-degree burns caused 39.5% muscle mass loss and 57.3% grip strength reduction, accompanied by PKC-λ activation and downstream STAT3-CCAAT/enhancer-binding protein delta (C/EBPδ)/myostatin catabolic signaling. Muscle-specific PKC-λ knockout mice showed significant protection against burn-induced muscle atrophy. *In vitro* studies demonstrated that PKC-λ regulates muscle catabolism through STAT3 activation, as STAT3 overexpression reversed protective effects of PKC-λ inhibition. Pharmacological PKC-λ inhibition with ICA-1 restored muscle mass to 85.5% and grip strength to 73.6% of control levels while suppressing the PKC-λ/STAT3-C/EBPδ catabolic cascade.

**Conclusion:**

PKC-λ serves as a critical upstream mediator linking severe burn injury to skeletal muscle atrophy through STAT3-C/EBPδ catabolic signaling. These findings establish PKC-λ as a promising therapeutic target for preventing burn-induced muscle wasting.

## Introduction

Severe burn injury represents one of the most devastating forms of trauma, triggering complex systemic pathophysiological responses that profoundly impact multiple organ systems (Jeschke et al., 2020). Among these complications, burn-induced skeletal muscle atrophy emerges as a critical issue that significantly compromises patient outcomes, rehabilitation potential, and long-term functional recovery (Knuth et al., 2021). Burn patients can lose up to 25% of their pre-injury lean body mass, with muscle wasting often persisting for months to years after the initial injury (Knuth et al., 2021; Klein, 2018). This progressive muscle atrophy is associated with delayed wound healing, increased susceptibility to infections, prolonged mechanical ventilation, and elevated mortality rates (Gao et al., 2024).

Current understanding of burn-induced muscle atrophy has identified the STAT3-C/EBPδ catabolic signaling cascade as a key molecular pathway driving muscle protein degradation (Ono et al., 2022). This well-characterized pathway links inflammatory responses to muscle wasting through activation of transcription factors that upregulate critical atrophy-related genes, including myostatin, MuRF1, and MAFbx, which encode E3 ubiquitin ligases responsible for muscle protein degradation (Ono et al., 2022). Previous studies have demonstrated that pharmacological inhibition of STAT3 signaling effectively prevents thermal burn-induced skeletal muscle wasting in experimental models (Ono et al., 2022). However, the upstream regulatory mechanisms that initiate and control this catabolic cascade in severe burn injury remain incompletely understood.

The severity of burn injury significantly influences the magnitude and persistence of muscle wasting, with third-degree burn injury (3rd) typically associated with more severe and prolonged muscle atrophy compared to second-degree burn injury (2nd) (Gao et al., 2025). This differential response suggests that distinct molecular mechanisms may be activated depending on burn severity, providing an opportunity to identify specific therapeutic targets for the most critically affected patients. Understanding these severity-dependent molecular signatures could inform the development of targeted interventions to prevent or mitigate muscle wasting.

Protein kinase C (PKC) family members represent attractive candidates for investigating upstream regulation of muscle catabolic pathways, given their diverse roles in cellular signaling, inflammatory responses, and metabolic regulation (Stabel and Parker, 1991; Itani et al., 2001). The PKC family comprises multiple isoforms categorized into three subfamilies based on their regulatory mechanisms: conventional PKCs (α, β), novel PKCs (δ, θ), and atypical PKCs (ζ, λ/ι, PKC-λ in humans and PKC-ι in mice, here we use PKC-λ throughout this study) (Dekker et al., 1995). Different PKC isoforms exhibit distinct tissue distributions, subcellular localizations, and responsiveness to various cellular stimuli, including inflammatory mediators and metabolic stress signals that are prominently elevated in severe burn injury (Kenchappa et al., 2021). Understanding which specific PKC isoforms are activated in burn-induced muscle atrophy could provide insights into the upstream molecular events that initiate muscle catabolic signaling cascades.

Current therapeutic approaches for burn-induced muscle atrophy remain limited and largely supportive, emphasizing the urgent need for mechanism-based interventions (Knuth et al., 2021). While nutritional support and physical therapy provide some benefit, these approaches often fail to prevent the profound muscle wasting observed in severe burn patients. The identification of specific molecular targets within the catabolic signaling networks could enable the development of more effective therapeutic strategies.

Therefore, the present study was designed to investigate the involvement of PKC isoforms in burn-induced muscle atrophy, elucidate the molecular mechanisms linking burn severity to muscle catabolic pathways, and evaluate potential therapeutic targets for preventing burn-induced muscle wasting. Our findings reveal a critical role for specific PKC signaling in severe burn-induced skeletal muscle atrophy and identify promising therapeutic approaches for improving outcomes in burn patients.

## Materials and methods

### Ethics statements

All human studies were conducted in accordance with the Declaration of Helsinki and approved by the Ethics Committee of the Central Hospital Affiliated of Shandong First Medical University (approval number: 2023-185-01). Written informed consent was obtained from all patients or their legal guardians prior to tissue collection.

All animal experiments were performed in accordance with the National Institutes of Health Guide for the Care and Use of Laboratory Animals and approved by the Institutional Animal Care and Use Committee of Central Hospital Affiliated of Shandong First Medical University.

### Human subjects

Skeletal muscle biopsies were obtained from burn patients admitted to the First Affiliated Hospital of Shandong University. Patients were categorized into two groups based on burn severity: second-degree burn injury (2nd, n = 5) and third-degree burn injury (3rd, n = 5). Inclusion criteria included: age 18–65 years, burn injury within 24 h of admission, and absence of pre-existing muscle disorders. Exclusion criteria included: pregnancy, history of neuromuscular disease, chronic inflammatory conditions, and concurrent use of corticosteroids. Muscle biopsies were collected from the vastus lateralis muscle at 7 days post-injury under local anesthesia and immediately snap-frozen in liquid nitrogen for subsequent analysis.

### Animals

Male C57BL/6J mice (8–10 weeks old, 22–25 g) were purchased from Beijing Vital River Laboratory Animal Technology Co., Ltd. (Beijing, China). Muscle-specific PKC-λ knockout (KO) mice were generated by crossing PKC-λ floxed mice with muscle creatine kinase (MCK)-Cre transgenic mice, both obtained from The Jackson Laboratory (Bar Harbor, ME, United States). Animals were housed in a controlled environment (22 °C ± 2 °C, 12-h light/dark cycle) with free access to standard laboratory chow and water. Mice were acclimatized for at least 7 days before experimental procedures.

### Burn injury model

Burn injuries were induced under isoflurane anesthesia (2%–3% in oxygen; RWD Life Science, Shenzhen, China) using a modified protocol. For 2nd burn injury, the dorsal skin was exposed to a heated brass block (85 °C) for 10 s, covering approximately 20% of total body surface area. For 3rd burn injury, the temperature was increased to 95 °C for 15 s. Sham-operated mice received identical anesthesia and handling without heat application. Post-injury, mice received subcutaneous normal saline (1 mL) for fluid resuscitation and buprenorphine (0.05 mg/kg, subcutaneous; Reckitt Benckiser, Hull, UK) for analgesia. For time-course experiments, separate cohorts of mice were euthanized at each indicated time point (0, 3, 7, 14 days post-injury).

### Cell culture and *in vitro* burn serum stimulation model

C2C12 mouse myoblasts (ATCC CRL-1772; American Type Culture Collection, Manassas, VA, United States) were cultured in Dulbecco’s Modified Eagle Medium (DMEM; Gibco, Thermo Fisher Scientific, Waltham, MA, United States) with 10% fetal bovine serum (FBS; Gibco) and 1% penicillin-streptomycin (Gibco). Cultures were maintained at 37 °C in a humidified 5% CO2 environment. To induce differentiation, confluent myoblasts were switched to a differentiation medium (DMEM with 2% horse serum; Gibco) for 5–7 days until mature myotubes formed.

Plasma was obtained from mice 14 days post-3rd burn injury or sham procedure via cardiac puncture using ethylenediaminetetraacetic acid (EDTA)-coated tubes (BD Biosciences, Franklin Lakes, NJ, United States). Plasma samples were grouped by treatment, heat-inactivated at 56 °C for 30 min in a block incubator (BI-516H; Astec, Shime, Japan), and added to the differentiation medium at 5% (vol/vol).

For PKC-λ inhibition, ICA-1 was dissolved in sterile distilled water and applied to cells at 5 μM, 1 h prior to plasma addition. Control cells received an equivalent volume of sterile distilled water. The 5 μM ICA-1 concentration was determined from preliminary dose-response experiments (1–10 μM range), where PKC-λ expression and phosphorylation (p^Thr555^-PKC-λ) showed no significant difference from the control (N), as shown in Supplementary Figure S1A.

For STAT3 overexpression, C2C12 myotubes were transfected with a lentiviral STAT3 plasmid (Shanghai GenePharma, Shanghai, China) or an empty vector control, following the manufacturer’s protocol.

### Drug treatment

ICA-1 (Selleck Chemicals, Houston, TX, United States), a selective PKC-λ inhibitor, was prepared in sterile distilled water. Mice were administered daily subcutaneous injections of ICA-1 (50 mg/kg) or vehicle control (sterile distilled water) starting immediately after burn injury for 14 days. The 50 mg/kg dose was selected based on preliminary studies (10–80 mg/kg range), where PKC-λ expression and phosphorylation (p^Thr555^-PKC-λ) showed no significant difference compared to the sham control, as detailed in Supplementary Figure S1B.

### Muscle weight measurement

At 14 days post-injury, mice were euthanized by CO_2_ asphyxiation, and tibialis anterior (TA) muscles were carefully dissected, trimmed of visible connective tissue, and weighed using an analytical balance (Sartorius BSA124S, Göttingen, Germany).

### Grip strength testing

Forelimb grip strength was measured using a grip strength meter (Columbus Instruments, Columbus, OH, United States) at 14 days post-injury. Each mouse underwent three consecutive trials with 1-min intervals, and the maximum force generated was recorded.

### Histological analysis

Tibialis anterior (TA) muscles were dissected, fixed in 4% paraformaldehyde, embedded in paraffin, and sectioned transversely at 5 μm thickness. Sections were stained with hematoxylin and eosin (H&E) for morphological assessment. Images were captured using a light microscope (Olympus BX53) at 20× magnification. For quantitative analysis of myofiber cross-sectional area (CSA), at least 200 myofibers per animal (n ≥ 3 per group) were manually traced using ImageJ software. All analyses were performed by an investigator blinded to the experimental groups.

### Immunofluorescence microscopy

C2C12 myotubes cultured on glass coverslips were fixed with 4% paraformaldehyde for 15 min, permeabilized with 0.1% Triton X-100 (Sigma-Aldrich) for 10 min, and blocked with 5% normal goat serum (Vector Laboratories, Burlingame, CA, United States) for 1 h at room temperature. Cells were incubated overnight at 4 °C with primary antibody against myosin heavy chain (MyHC; 1:500 dilution; Developmental Studies Hybridoma Bank, Iowa City, IA, United States), followed by Alexa Fluor 594-conjugated secondary antibody (1:1,000; Thermo Fisher Scientific) for 1 h at room temperature. Nuclei were counterstained with 4′,6-diamidino-2-phenylindole (DAPI; 1 μg/mL; Sigma-Aldrich). Images were acquired using a fluorescence microscope (Olympus IX71) and analyzed using ImageJ software. Fusion index was calculated as the percentage of nuclei within multinucleated myotubes (≥3 nuclei) relative to total nuclei counted.

### Western blot analysis and co-immunoprecipitation (Co-IP)

Tissue samples and cultured cells were lysed in RIPA buffer (Thermo Fisher Scientific) supplemented with protease inhibitor cocktail (Roche Diagnostics, Basel, Switzerland) and phosphatase inhibitor cocktail (Sigma-Aldrich). Protein concentrations were determined using the BCA Protein Assay Kit (Pierce, Thermo Fisher Scientific). Equal amounts of protein (30–50 μg) were separated by SDS-PAGE using 4%–20% gradient gels (Bio-Rad Laboratories, Hercules, CA, United States) and transferred to polyvinylidene fluoride (PVDF) membranes (Millipore, Burlington, MA, United States). Membranes were blocked with 5% non-fat dry milk in Tris-buffered saline containing 0.1% Tween-20 (TBST) for 1 h at room temperature.

For co-immunoprecipitation, cell lysates were incubated with 2 μg of anti-PKC-λ antibody or normal rabbit IgG overnight at 4 °C, followed by incubation with Protein A/G agarose beads for 2 h at 4 °C. Bound proteins were eluted by boiling in SDS loading buffer for Western blot analysis.

Primary antibodies were used at the following dilutions: PKC-α (M1-157, Thermo Fisher Scientific, 1:1,000), phospho-PKC-α Thr638 (44–962G, Thermo Fisher Scientific, 1:1,000), PKC-β (MA5-51282, Thermo Fisher Scientific, 1:1,000), phospho-PKC-β Ser661 (PA5-105770, Thermo Fisher Scientific, 1:1,000), PKC-δ (#2058, Cell Signaling Technology, Danvers, MA, United States, 1:1,000), phospho-PKC-δ Tyr311 (#2055, Cell Signaling Technology, 1:1,000), PKC-θ (#13634, Cell Signaling Technology, 1:1,000), phospho-PKC-θ Tyr90 (PA5-99571, Thermo Fisher Scientific, 1:1,000), PKC-ζ (ab108970, Abcam, Cambridge, United Kingdom, 1:1,000), phospho-PKC-ζ Thr560 (ab62372, Abcam, 1:1,000), PKC-λ (#2998, Cell Signaling Technology, 1:1,000), phospho-PKC-λ Thr557/Thr564 (44–968G, Thermo Fisher Scientific, 1:1,000), STAT3 (MA5-15712, Thermo Fisher Scientific, 1:1,000), phospho-STAT3 Tyr705 (ab76315, Abcam, 1:1,000), C/EBPδ (EPR23518-259, Abcam, 1:1,000), myostatin (PA5-11936, Thermo Fisher Scientific, 1:1,000), MuRF1 (#4305, Cell Signaling Technology, 1:1,000), MAFbx (sc-166806, Santa Cruz Biotechnology, Dallas, TX, United States, 1:1,000), and β-tubulin (#2128, Cell Signaling Technology, 1:2000).

Membranes were incubated with primary antibodies overnight at 4 °C, followed by horseradish peroxidase-conjugated secondary antibodies (1:5,000; Cell Signaling Technology) for 1 h at room temperature. Protein bands were visualized using enhanced chemiluminescence (ECL) detection reagent (Pierce ECL, Thermo Fisher Scientific) and captured using a ChemiDoc imaging system (Bio-Rad Laboratories). Band intensities were quantified using ImageJ software. For all PKC isoforms, the phosphorylated form was first normalized to the total protein of the same isoform (p-PKC/total PKC) for each sample. For p-STAT3, it was normalized to total STAT3. These ratios were then normalized to β-tubulin when total protein expression varied between groups. For time-course experiments, all values were expressed as fold change relative to the sham group.

### Quantitative real-time polymerase chain reaction (PCR)

Total RNA was extracted from cultured cells using TRIzol reagent (Thermo Fisher Scientific) according to the manufacturer’s protocol. RNA quality and concentration were assessed using a NanoDrop spectrophotometer (Thermo Fisher Scientific). First-strand cDNA synthesis was performed using 1 μg of total RNA with the PrimeScript RT reagent kit (Takara Bio, Kusatsu, Japan). Quantitative PCR was performed using SYBR Green PCR Master Mix (Applied Biosystems, Foster City, CA, United States) on a StepOnePlus Real-Time PCR System (Applied Biosystems).

Primer sequences were as follows: *STAT3* forward 5′-CAC​CTT​GGA​TTG​AGA​GTC-AAG​AC-3′, reverse 5′-AGG​AAT​CGG​CTA​TAT​TGC​TGG​T-3'; *PKC-λ* forward 5′-CCA-CAC​TTT​TCA​AGC​CAA​ACG-3′, reverse 5′-TGC​ACT​TGT​ATC​CTT​GTC​GTC-3'; *C/EBPδ* forward 5′-CGA​CTT​CAG​CGC​CTA​CAT​TGA-3′, reverse 5′-CTA​GCG​A-CAG​ACC​CCA​CAC-3'; *myostatin* forward 5′-AGT​GGA​TCT​AAA​TGA​GGG​CAG​T-3′, reverse 5′-GGA​GTA​CCT​CGT​GTT​TTG​TCT​C-3'; *MuRF1* forward 5′-GTG​TGA​GG-TGC​CTA​CTT​GCT​C-3′, reverse 5′-TGA​GAG​ATG​ATC​GTC​TGC​ACT-3'; *MAFbx* forward 5′-CAG​CTT​CGT​GAG​CGA​CCT​C-3′, reverse 5′-GGC​AGT​CGA​GAA​GTC​C-AGT​C-3'; 18S rRNA forward 5′-AGT​CCC​TGC​CCT​TTG​TAC​ACA-3′, reverse 5′-CGA​T-CCG​AGG​GCC​TCA​CTA-3'. Relative gene expression was calculated using the 2^(−ΔΔCt)^ method with 18S rRNA as the internal control.

### Statistical analysis

All data are presented as mean ± standard error of the mean (SEM). Statistical analyses were performed using GraphPad Prism version 9.0 software (GraphPad Software, San Diego, CA, United States). For comparisons between two groups, unpaired Student's t-tests were used. For multiple group comparisons, one-way analysis of variance (ANOVA) followed by Tukey’s *post hoc* test was employed. Two-way ANOVA was used for time-course analyses with Bonferroni’s multiple comparisons test. Statistical significance was set at P < 0.05. All experiments were performed at least three times independently, and sample sizes (n) are indicated in the figure legends.

## Results

### PKC-λ expression and activation are selectively upregulated in severe burn injury

To elucidate the involvement of protein kinase C (PKC) isoforms in burn-induced skeletal muscle atrophy, we examined the expression and phosphorylation status of six PKC isoforms (α, β, δ, θ, ζ, and λ) in skeletal muscle samples from burn patients and experimental mouse models.

**FIGURE 1:**
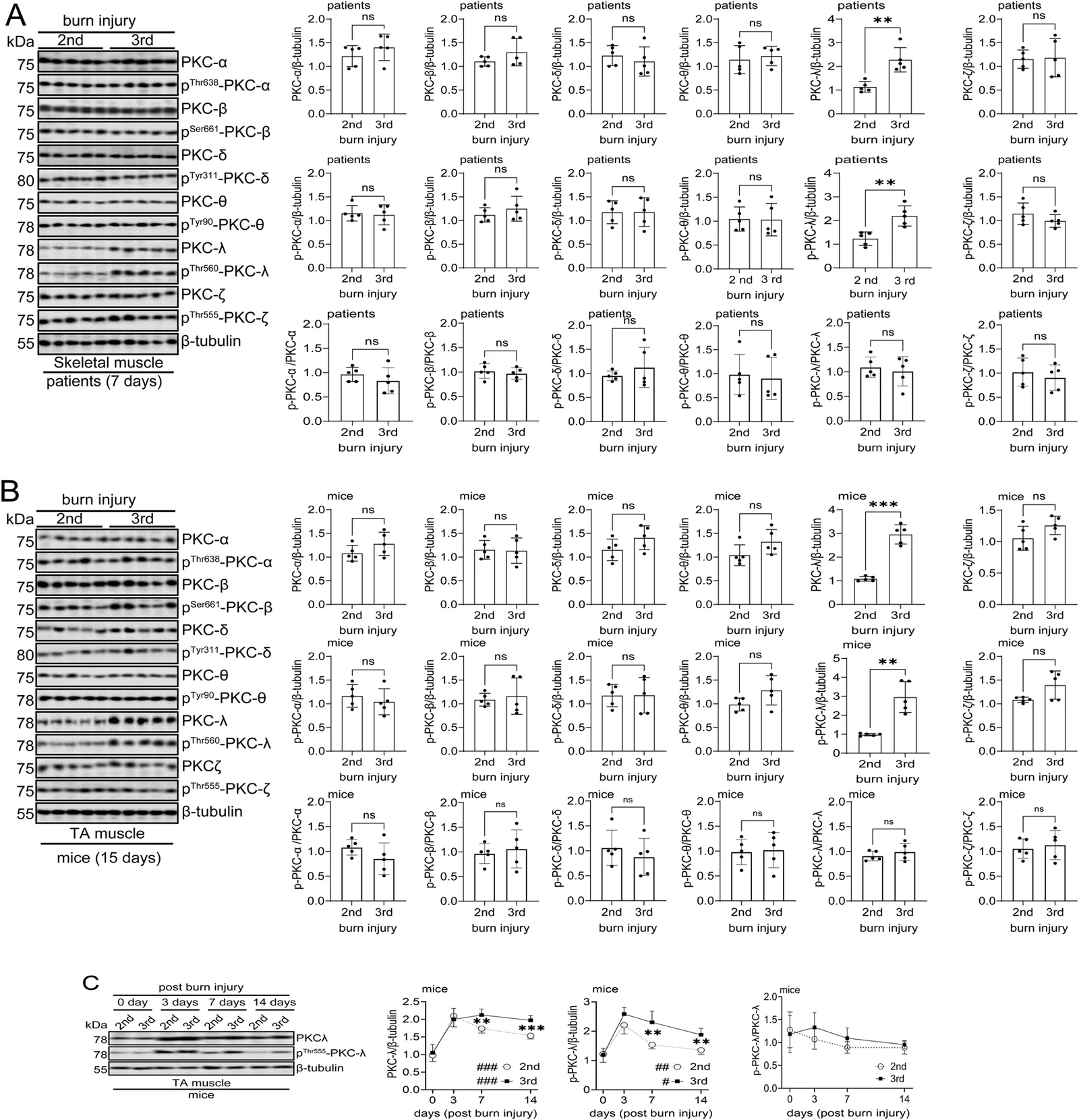
Expression and activation of PKC isoforms in skeletal muscle following second-degree and third-degree burn injuries. **(A)** Representative Western blots and quantitative analysis of PKC isoforms (α, β, δ, θ, ζ, λ) and their phosphorylated forms (p^Thr638^-PKC-α, p^Ser661^-PKC-β, p^Tyr331^-PKC-δ, p^Tyr90^-PKC-θ, p^Thr560^-PKC-ζ, p^Thr555^-PKC-λ) in skeletal muscle biopsies from patients with 2nd and 3rd burn injury at 7 days post-injury. β-tubulin served as loading control. Right panel: Quantification of PKC isoforms expression and phosphorylation levels (normalized to β-tubulin) in patient samples. Data represent mean ± SEM (n = 5 per group). **P < 0.01; ns, not significant. **(B)** Representative Western blots and quantitative analysis of PKC isoforms and their phosphorylated forms in TA muscle from mice with 2nd and 3rd burn injury at 14 days post-injury. β-tubulin served as loading control. Right panel: Quantification of PKC isoforms expression and phosphorylation levels (normalized to β-tubulin) in patient samples. Data represent mean ± SEM (n = 5 per group). **P < 0.01, ***P < 0.001; ns, not significant. **(C)** Time-course analysis of PKC-λ expression and phosphorylation (p^Thr555^-PKC-λ) in mouse TA muscle at 0, 3, 7, and 14 days following 2nd and 3rd burn injury. Samples were obtained from different mice at each indicated time point. Left panel shows representative Western blots with β-tubulin as loading control. Right panels show quantitative analysis of PKC-λ expression and phosphorylation levels normalized to β-tubulin. Data represent mean ± SEM (n = 5 per group). Comparisons between 2nd vs. 3rd burn injury: **P < 0.01, ***P < 0.001; ns, not significant. Full-length membranes with molecular weight markers for human PKC-λ, human PKC-ζ, and mouse PKC-λ are shown in the Supplementary Material. For mouse PKC-ζ, the marker lane was not retained; however, because this panel was run on a gel adjacent to the mouse PKC-λ panel under identical conditions, the PKC-λ markers serve as a reliable reference for band positions.

Analysis of skeletal muscle biopsies from burn patients demonstrated isoform-specific alterations in PKC signaling. At 7 days post-injury, patients with 3rd burn injury (n = 5) exhibited significantly elevated PKC-λ protein expression and phosphorylation levels compared to patients with 2nd burn injury (n = 5) (Figure 1A). In contrast, the expression and phosphorylation of other PKC isoforms (PKC-α, -β, -δ, -θ, and -ζ) remained unchanged between the two groups (P > 0.05 for all comparisons). Notably, PKC-ζ and PKC-λ have similar molecular weights and exhibit comparable basal expression levels in muscle tissue.

These clinical observations were validated in a controlled mouse burn model. Tibialis anterior (TA) muscles from mice with 3rd burn injury showed significantly increased PKC-λ expression and phosphorylation at 14 days post-injury compared to mice with 2nd burn injury (Figure 1B). Consistent with the human data, other PKC isoforms displayed no significant differences between burn severity groups (P > 0.05 for all isoforms except PKC-λ).

To examine the temporal activation pattern of PKC-λ in response to burn injury, we performed a time-course analysis in mouse TA muscle at 0, 3, 7, and 14 days post-injury (Figure 1C). While PKC-λ expression and phosphorylation levels were comparable between 2nd and 3rd burn injury groups at 3 days post-injury (P > 0.05), significant upregulation became evident in the 3rd burn injury group at 7 days (P < 0.01) and further increased at 14 days post-injury (P < 0.001), indicating sustained activation of PKC-λ signaling in severe burn conditions.

To exclude the possibility that other PKC isoforms might also be activated in a time-dependent manner, we analyzed the expression and phosphorylation of PKC-ζ. Using the same murine muscle samples (at 0, 3, 7, and 14 days post-injury), we found no significant differences in total PKC-ζ protein levels or p-PKC-ζ (Tyr311) levels between the grade 2 and grade 3 burn groups at any time point (Supplementary Figure S1). These data indicate that, among the six PKC isoforms examined, only PKC-λ exhibited sustained and severity-dependent activation following severe burn injury.

### PKC-λ mediates third-degree burn-induced muscle atrophy through activation of catabolic signaling pathways

**FIGURE 2:**
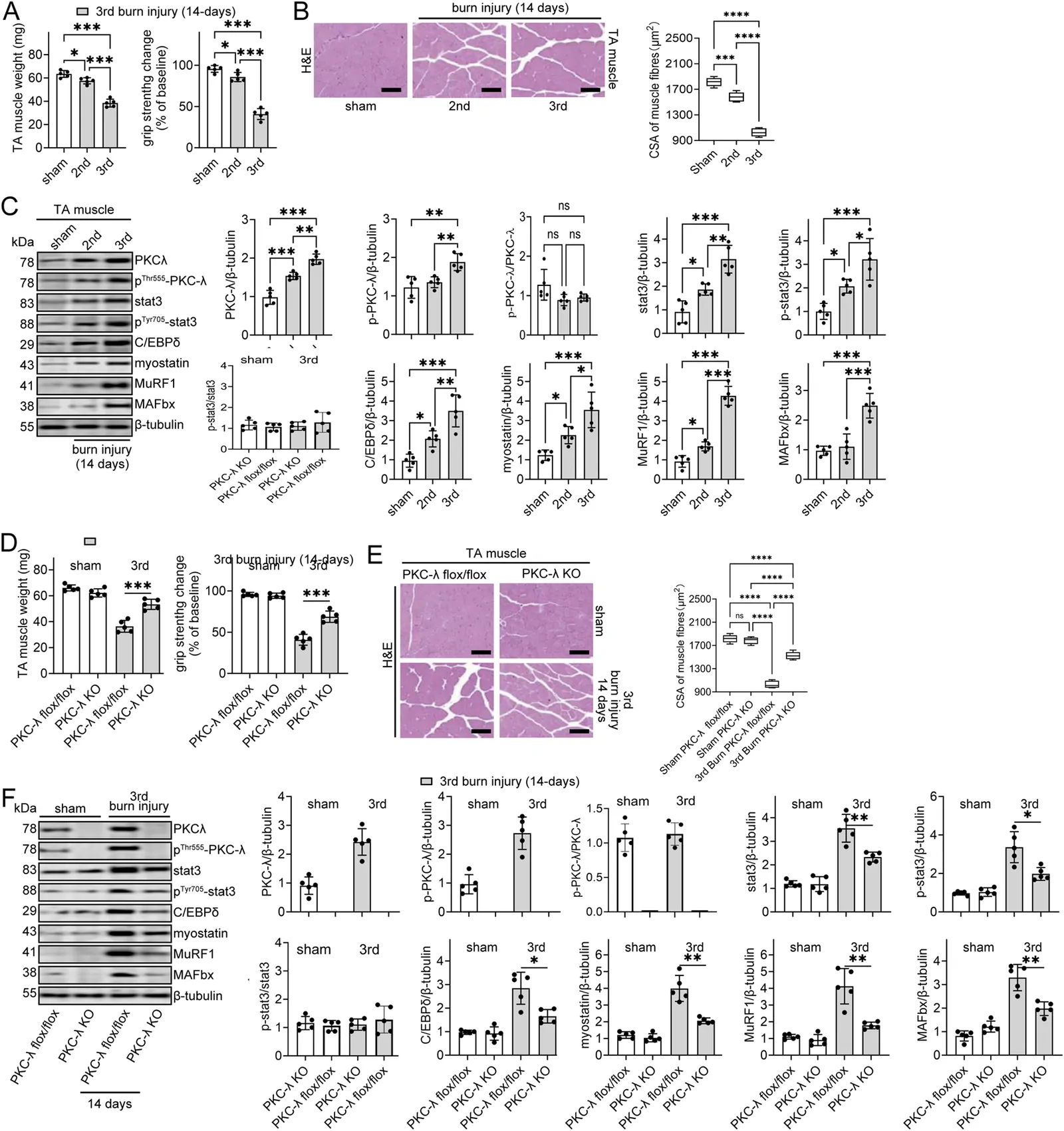
PKC-λ mediates third-degree burn-induced muscle wasting via activation of catabolic signaling pathways. **(A)** TA muscle weight (left panel) and forelimb grip strength (right panel) in mice 14 days following sham procedure, 2nd burn injury and 3rd burn injury. Data are presented as mean ± SEM (n = 5 per group). **P < 0.01, ***P < 0.001. **(B)** Haematoxylin–eosin (H&E) staining of TA muscles (scale bar, 50 μm) and cross-sectional area (CSA) of muscle fibres. **(C)** Representative Western blot analysis of PKC-λ, phospho-PKC-λ (p^Thr555^-PKC-λ), STAT3, phospho-STAT3 (p^Tyr705^-STAT3), C/EBPδ, myostatin, MuRF1, MAFbx, and β-tubulin (loading control) in TA muscle lysates from sham, 2nd burn injury, and 3rd burn injury mice at 14 days post-injury. Right panels: Densitometric quantification of protein expression levels normalized to β-tubulin. Data are presented as mean ± SEM (n = 5 per group). *P < 0.05, **P < 0.01, ***P < 0.001. **(D)** TA muscle weight (left panel) and forelimb grip strength (right panel) in PKC-λ flox/flox and muscle-specific PKC-λ knockout (KO) mice at 14 days following sham procedure or 3rd burn injury. Data are presented as mean ± SEM (n = 5 per group). ***P < 0.001. **(E)** Representative H&E stained cross-sections of TA muscle from PKC-λ flox/flox and PKC-λ KO mice at 14 days following sham procedure or 3rd burn injury and CSA of muscle fibres. Scale bar = 50 μm. **(F)** Representative Western blot analysis of PKC-λ, phospho-PKC-λ (p^Thr555^-PKC-λ), STAT3, phospho-STAT3 (p^Tyr705^-STAT3), C/EBPδ, myostatin, MuRF1, MAFbx, and β-tubulin in TA muscle lysates from PKC-λ flox/flox and PKC-λ KO mice at 14 days following sham procedure or 3rd burn injury. Right panels: Densitometric quantification of protein expression levels normalized to β-tubulin. Data are presented as mean ± SEM (n = 5 per group). *P < 0.05, **P < 0.01 vs. corresponding sham group.

To investigate the role of PKC-λ in burn-induced muscle atrophy, we evaluated muscle mass, functional capacity, and histological changes in mice with varying burn severities. At 14 days post-injury, mice with 3rd burn injury demonstrated significant reductions in TA muscle weight (39.5% decrease, P < 0.001) and grip strength (57.3% decrease, P < 0.001) compared to sham controls, while 2nd burn injury showed only modest changes (Figure 2A). Histological examination and CSA quantitative analysis showed that, compared with the sham group, the TA muscle fibers of burn mice exhibited varying degrees of atrophy. Specifically, the intermuscular spaces were increased in mice with second-degree burns, while in mice with third-degree burns, the muscle fiber atrophy was most severe, the intermuscular spaces were significantly widened, and the muscle tissue structure was markedly disrupted (Figure 2B).

We next examined the molecular mechanisms underlying this burn injury associated muscle atrophy in TA muscle. Western blot analysis revealed that 3rd burn injury significantly upregulated PKC-λ expression and phosphorylation compared to 2nd burn injury and sham controls (Figure 2C). Concomitantly, key catabolic mediators including the expression and phosphorylation levels of STAT3, C/EBPδ, myostatin, MuRF1, and MAFbx were markedly elevated in 3rd burn injury mice, indicating activation of muscle protein degradation pathways.

To establish the causal relationship between PKC-λ and burn-induced muscle wasting, we employed muscle-specific PKC-λ KO mice. 3rd burn injury-induced reductions in TA muscle weight and grip strength were significantly attenuated in PKC-λ KO mice compared to PKC-λ flox/flox controls (Figure 2D). Histological and CSA quantitative analysis confirmed that muscle fiber atrophy and structural deterioration were markedly reduced in PKC-λ KO mice following 3rd burn injury (Figure 2E). Importantly, genetic ablation of PKC-λ substantially diminished the expression of catabolic markers, including the expression and phosphorylation levels of STAT3, C/EBPδ, myostatin, MuRF1, and MAFbx, in burn-injured muscle (Figure 2F). These findings demonstrate that PKC-λ is a critical mediator of burn-induced muscle atrophy through its regulation of catabolic signaling pathways.

### In C2C12 myotubes, PKC-λ regulates muscle catabolic pathways via STAT3

STAT3 is considered a key factor involved in muscle catabolic pathways. To investigate whether STAT3 functions downstream of PKC-λ in the context of burn injury associated muscle atrophy, we conducted *in vitro* experiments using C2C12 myotubes. After 48 h of treatment, the myotubes were divided into four experimental groups: untreated/blank (controls, wild-type cells with serum from sham mice and empty vector), serum/blank (cells treated with serum from 3rd burn injury mice and empty vector), serum/blank/ICA-1 (cells treated with 3rd burn injury serum plus 5 µM PKC-λ inhibitor ICA-1 and empty vector), and serum/ICA-1/STAT3-OE (cells treated with 3rd burn injury serum plus 5 µM ICA-1 and STAT3 overexpression vector).

**FIGURE 3:**
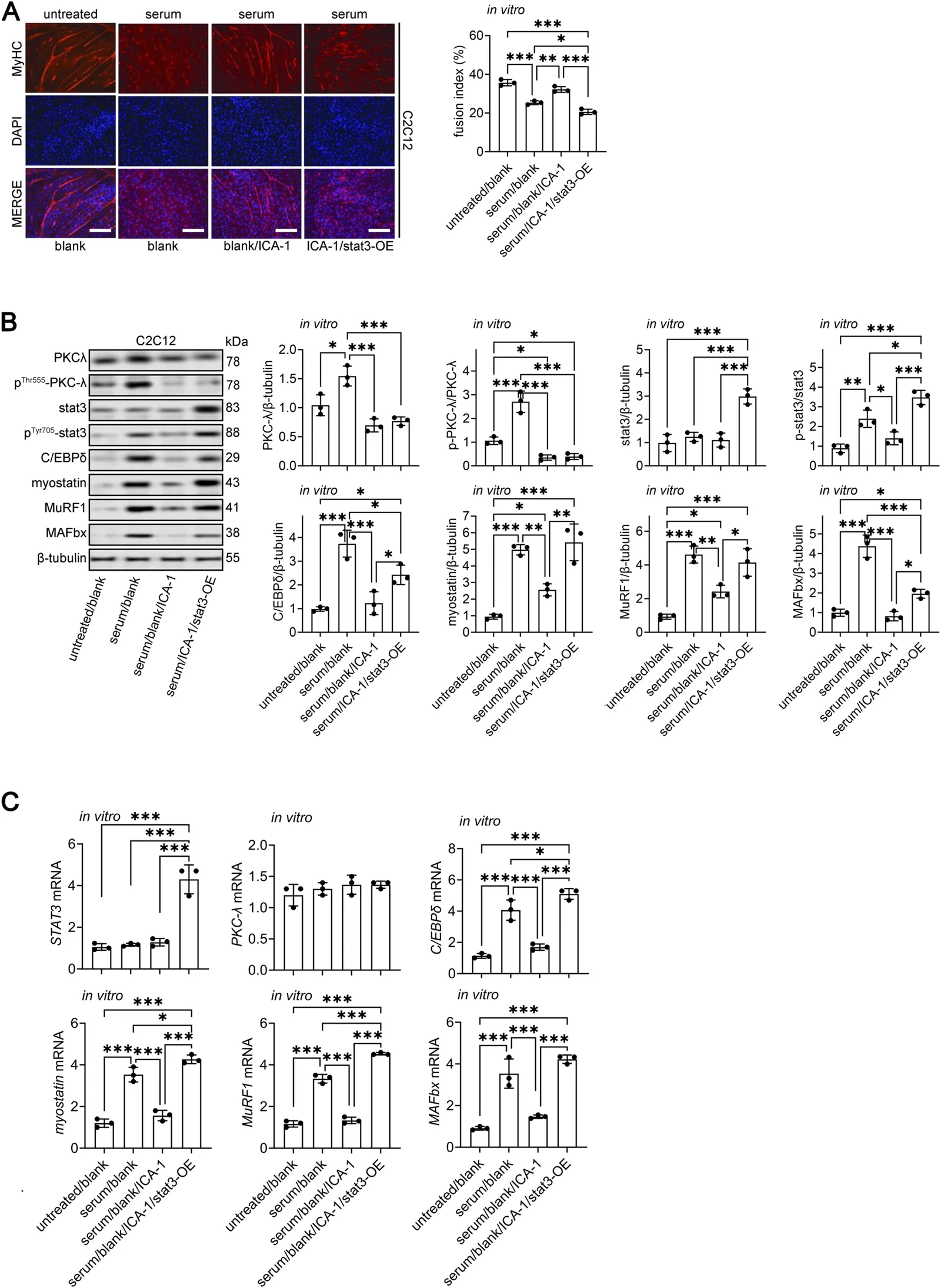
*In vitro* STAT3 overexpression reverses the protective effects of ICA-1-mediated PKC-λ inhibition on myotube formation and catabolic pathways. C2C12 myotubes were divided into four groups: untreated/blank (controls, wild-type cells with untreated serum and empty vector), serum/blank (cells treated with serum from 3rd burn injury mice and empty vector), serum/blank/ICA-1 (cells treated with 3rd burn injury serum plus 5 µM ICA-1 and empty vector), and serum/ICA-1/STAT3-OE (cells treated with 3rd burn injury serum plus 5 µM ICA-1 and STAT3 overexpression vector). **(A)** Representative immunofluorescence images showing MyHC (red) and nuclei (DAPI, blue) staining in C2C12 myotubes. Scale bar represents 100 μm. Right panel shows quantification of fusion index calculated as the percentage of nuclei within multinucleated myotubes. Data are presented as mean ± SEM from three independent experiments. *P < 0.05, **P < 0.01, ***P < 0.001. **(B)** Representative Western blot analysis of PKC-λ, phospho-PKC-λ (p^Thr555^-PKC-λ), STAT3, phospho-STAT3 (p^Tyr705^-STAT3), C/EBPδ, myostatin, MuRF1, MAFbx, and β-tubulin (loading control) in C2C12 myotubes. Right panels show densitometric quantification of protein expression levels normalized to β-tubulin. Data are presented as mean ± SEM from three independent experiments. *P < 0.05, **P < 0.01, ***P < 0.001. **(C)** Quantitative real-time PCR analysis of mRNA expression levels for STAT3, C/EBPδ, myostatin, MuRF1, and MAFbx. Data are normalized to 18S rRNA and expressed as fold change relative to controls. Values represent mean ± SEM from three independent experiments performed in triplicate. *P < 0.05, ***P < 0.001.

Immunofluorescence analysis (Figure 3A) revealed that treatment with serum from burn-injured mice (serum/blank group) significantly reduced the myotube fusion index compared to controls (untreated/blank group). PKC-λ inhibition with ICA-1 (serum/blank/ICA-1 group) significantly restored the fusion index, indicating improved myotube formation. However, STAT3 overexpression (serum/ICA-1/STAT3-OE group) completely reversed the protective effects of PKC-λ inhibition, demonstrating that STAT3 acts downstream of PKC-λ in regulating myotube development.

We next examined the protein levels of catabolic signaling components in the four groups. Western blot analysis (Figure 3B) demonstrated that burn serum treatment (serum/blank group) significantly increased PKC-λ expression and phosphorylation, STAT3 phosphorylation, and the expression of key catabolic markers including C/EBPδ, myostatin, MuRF1, and MAFbx compared to controls (untreated/blank group). ICA-1 treatment (serum/blank/ICA-1 group) effectively suppressed PKC-λ activation, STAT3 phosphorylation, and downstream catabolic protein expression. Importantly, STAT3 overexpression (serum/ICA-1/STAT3-OE group) not only restored STAT3 levels and phosphorylation but also reversed the suppression of catabolic markers induced by PKC-λ inhibition, confirming that STAT3 functions downstream of PKC-λ in this signaling cascade.

Quantitative PCR analysis further supported these findings at the transcriptional level (Figure 3C). STAT3 overexpression (serum/ICA-1/STAT3-OE group) significantly upregulated mRNA levels of *STAT3*, *C/EBPδ*, *myostatin*, *MuRF1*, and *MAFbx* compared to ICA-1 treatment (serum/ICA-1/STAT3-OE group). These results demonstrate that PKC-λ regulates muscle catabolic gene transcription through STAT3 activation, which subsequently drives the expression of key atrophy-related genes.

To determine whether PKC-λ physically interacts with STAT3, co-immunoprecipitation (Co-IP) experiments were performed in C2C12 myotubes treated with burn serum. As shown in Supplementary Figure S2, co-immunoprecipitation experiments revealed that PKC-λ interacted with STAT3 specifically in burn serum-treated C2C12 myotubes. These findings suggest that burn injury promotes the formation of a PKC-λ-STAT3 protein complex, further supporting a close functional relationship between the two molecules in the catabolic cascade.

### Pharmacological PKC-λ inhibition ameliorates muscle atrophy in third-degree burn mice

To investigate the therapeutic potential of PKC-λ inhibition in burn-induced muscle wasting, we administered 50 mg/kg ICA-1, a selective PKC-λ inhibitor, to 3rd burn injury mice and evaluated functional, histological, and molecular parameters at 14 days post-injury.

**FIGURE 4:**
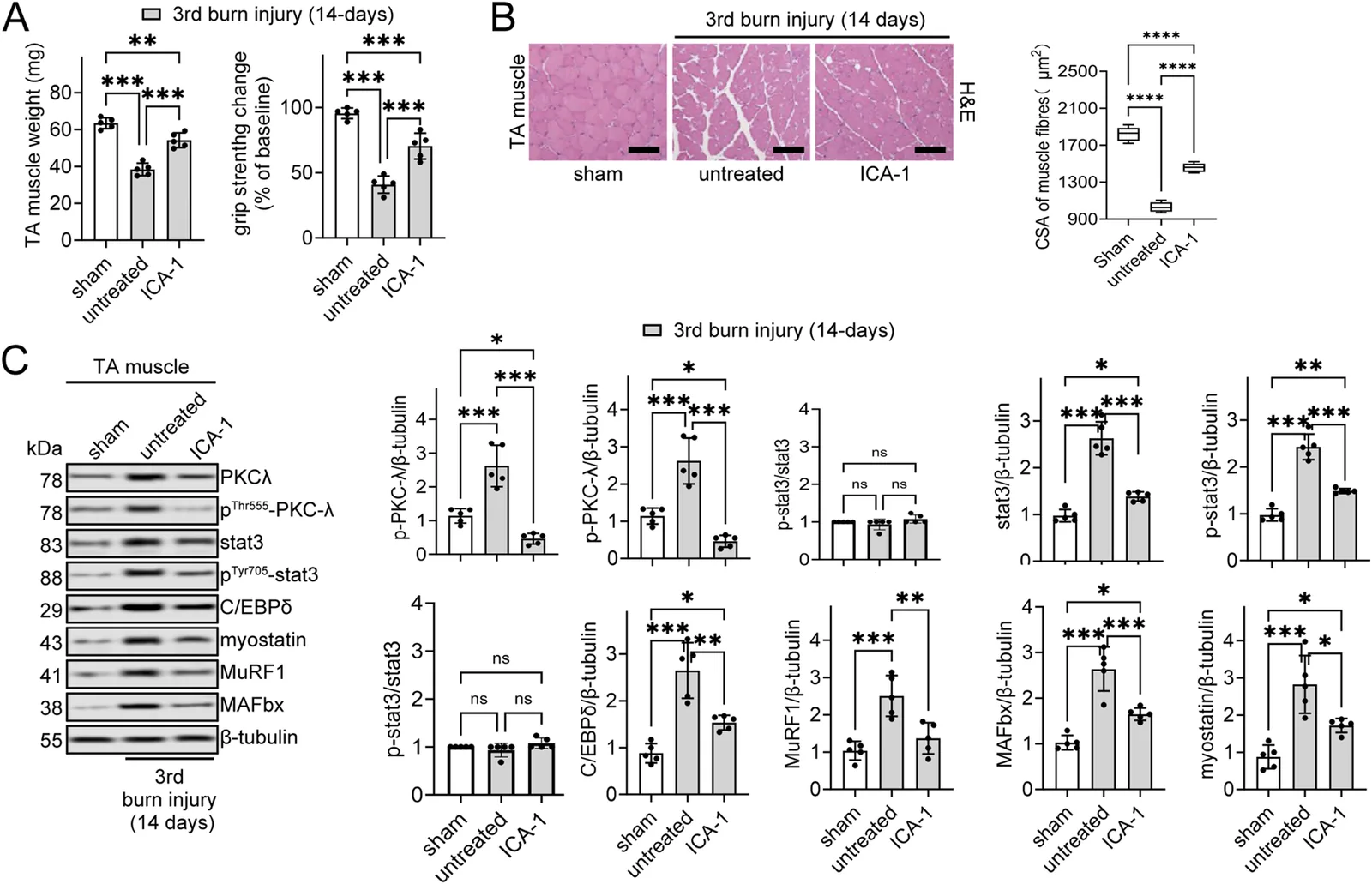
ICA-1 treatment mitigates muscle wasting in third-degree burn mice by suppressing PKC-λ mediated catabolic pathways. **(A)** Quantitative analysis of TA muscle weight (left panel) and grip strength (right panel) in sham-operated controls (sham group), vehicle-treated 3rd burn injury mice (untreated group), and 50 mg/kg ICA-1-treated 3rd burn injury mice (ICA-1 group) at 14 days post-injury. Data are presented as mean ± SEM (n = 5 mice per group). ***P < 0.001. **(B)** Representative hematoxylin and eosin (H&E)-stained cross-sections and CSA analysis of TA muscle from sham, untreated, and ICA-1 group mice at 14 days post-3rd burn injury. Scale bar = 50 μm. **(C)** Representative Western blot analysis of PKC-λ, phospho-PKC-λ (p^Thr555^-PKC-λ), STAT3, phospho-STAT3 (p^Tyr705^-STAT3), C/EBPδ, myostatin, MuRF1, MAFbx, and β-tubulin (loading control) in TA muscle lysates from sham, untreated, and ICA-1 group mice at 14 days post-3rd burn injury. Right panels show densitometric quantification of protein expression levels normalized to β-tubulin. Data are presented as mean ± SEM (n = 5 mice per group). *P < 0.05, **P < 0.01, ***P < 0.001.

Functional assessment demonstrated that 3rd burn injury (untreated group) significantly reduced TA muscle weight by approximately 39.5% and grip strength by 57.3% compared to sham group (controls; P < 0.001; Figure 4A). Treatment with ICA-1 (ICA-1 group) significantly attenuated these burn-induced deficits, restoring TA muscle weight to 85.5% and grip strength to 73.6% of sham control levels (P < 0.001 vs. untreated group; Figure 4A).

Histological examination of TA muscle cross-sections revealed marked structural deterioration in untreated group mice, characterized by extensive muscle fiber atrophy and increased interstitial space compared to sham controls (Figure 4B). ICA-1 treatment substantially preserved muscle architecture, with notably reduced fiber atrophy and minimal interstitial space expansion compared to untreated group mice (Figure 4B).

Molecular analysis by Western blot revealed significant upregulation of key components of the PKC-λ mediated catabolic pathway in TA muscle of untreated burn mice (Figure 4C). Specifically, burn injury of untreated group mice increased PKC-λ protein expression and phosphorylation (p^Thr555^-PKC-λ) by 2.6-fold and 2.3-fold, respectively, compared to sham controls (P < 0.001). Similarly, STAT3 expression and phosphorylation (p^Tyr705^-STAT3), C/EBPδ, myostatin, MuRF1, and MAFbx protein levels were significantly elevated in untreated group mice (2.4- to 3.2-fold increases; P < 0.001 vs. sham group). ICA-1 treatment (ICA-1 group) significantly suppressed the expression of all these catabolic markers, reducing their levels to near-sham control values (P < 0.05 to P < 0.001 vs. untreated group; Figure 4C).

These results demonstrate that pharmacological inhibition of PKC-λ with ICA-1 effectively mitigates burn-induced muscle atrophy through suppression of the PKC-λ/STAT3-C/EBPδ catabolic signaling cascade.

## Discussion

This study provides the first comprehensive evidence that PKC-λ serves as a critical mediator of severe burn-induced skeletal muscle atrophy through activation of the STAT3-C/EBPδ catabolic signaling cascade. Our findings demonstrate that PKC-λ is selectively upregulated in 3rd burn injury compared to 2nd burn injury in both human patients and experimental mouse models, and that both genetic ablation and pharmacological inhibition of PKC-λ significantly attenuate muscle wasting in severe burn injury. Most importantly, we reveal for the first time that phosphorylation at threonine 555 (Thr555) plays a crucial role in PKC-λ mediated muscle catabolic pathways following severe burn injury, representing a novel regulatory mechanism in burn-induced muscle atrophy.

Our comprehensive analysis of six PKC isoforms (α, β, δ, θ, ζ, and λ) in burn-induced muscle atrophy reveals remarkable specificity for PKC-λ activation, while other isoforms remained unchanged regardless of burn severity. Time-course analysis of PKC-ζ further demonstrated that neither total PKC-ζ nor p-PKC-ζ (Tyr311) levels differed between 2nd and 3rd degree burns at any post-injury day (3, 7, 14), reinforcing that PKC-λ is the uniquely responsive isoform in severe burn-induced muscle wasting. This isoform-specific response is particularly noteworthy given that PKC-λ belongs to the atypical PKC subfamily, which differs from conventional and novel PKC isoforms in its independence from diacylglycerol and calcium for activation. Instead, atypical PKCs are primarily regulated by protein-protein interactions and phosphorylation events, making them responsive to different upstream signals compared to other PKC family members (Reina-Campos et al., 2019). To definitively establish the causal role of PKC-λ in burn-induced muscle atrophy, we employed muscle-specific PKC-λ KO mice, a well-characterized model that has been previously utilized to investigate PKC-λ function in skeletal muscle metabolism. Previous studies using these KO mice have demonstrated that muscle-specific deletion of PKC-λ impairs glucose transport and induces metabolic abnormalities characteristic of diabetic syndromes, while PKC-λ haploinsufficiency has been shown to prevent diabetes through alterations in hepatic enzyme activities (Farese et al., 2007). However, the role of PKC-λ in muscle wasting conditions, particularly in the context of burn injury, has not been previously investigated, making our study the first to explore this connection. The significant preservation of muscle mass, strength, and histological architecture in PKC-λ KO mice following 3rd burn injury demonstrates that this kinase plays a causative role in the pathogenesis of burn-induced muscle atrophy.

The STAT3-C/EBPδ catabolic signaling cascade represents one of the most extensively characterized molecular pathways driving skeletal muscle wasting. Previous studies have demonstrated that C188-9, a specific inhibitor of STAT3 signaling, effectively prevents thermal burn-induced skeletal muscle wasting in mice, establishing the critical role of STAT3 activation in burn-related muscle atrophy (Ono et al., 2022; Di and Zhang, 2019). Furthermore, research investigating cancer cachexia has shown that STAT3 activation in skeletal muscle directly links muscle wasting to the acute phase response, demonstrating the broad relevance of this pathway across different wasting conditions (Zheng et al., 2024). The downstream effector C/EBPδ serves as a key transcriptional regulator that links STAT3 activation to myostatin pathway stimulation, ultimately promoting loss of muscle mass through upregulation of critical atrophy-related genes including MuRF1 (muscle RING finger protein 1) and MAFbx (muscle atrophy F-box protein), which encode E3 ubiquitin ligases responsible for muscle protein degradation. Our novel finding that PKC-λ functions upstream of this classical catabolic cascade in burn-induced muscle atrophy provides important mechanistic insights into how severe burn injury initiates muscle wasting. The ability of both genetic PKC-λ deletion and pharmacological PKC-λ inhibition to suppress STAT3 phosphorylation, C/EBPδ expression, myostatin upregulation, and downstream atrophy markers (MuRF1 and MAFbx) demonstrates that PKC-λ serves as a critical upstream regulator of this well-established pathway. This represents the first demonstration of PKC-λ involvement in regulating the STAT3-C/EBPδ/myostatin axis in any muscle wasting condition, expanding our understanding of the molecular networks controlling muscle catabolism. We further demonstrated by co-immunoprecipitation that PKC-λ physically interacts with STAT3 under catabolic conditions. While this interaction supports a direct regulatory axis, it does not prove that PKC-λ directly phosphorylates STAT3. Given that STAT3 Tyr705 is a canonical JAK substrate, PKC-λ may facilitate STAT3 activation through intermediate signaling events. Future studies using *in vitro* kinase assays are required to determine whether PKC-λ directly phosphorylates STAT3 or acts via other kinases.

The selection of 7-day post-injury timepoints for human burn patients and 14-day endpoints for mouse studies was based on established evidence in the burn literature. Previous studies have demonstrated that the hypermetabolic and hypercatabolic responses become fully established by 7 days post-injury in humans, with significant muscle protein breakdown and lean body mass loss detectable at this timepoint (Greabu et al., 2021). The 14-day endpoint for mouse studies was chosen based on extensive precedent in burn cachexia research, where studies have consistently shown that inflammation, organomegaly, muscle wasting, compromised muscle regenerative capacity, and neuronal changes are well-established by this timepoint despite hyperphagia. This timeline accounts for species-specific differences in metabolic rates while ensuring consistency with established experimental protocols in the burn research field. Our temporal analysis revealing progressive PKC-λ activation from 7 to 14 days in mice validates the appropriateness of these selected timepoints and demonstrates that both human and mouse studies captured periods of active catabolic signaling.

Based on these mechanistic insights, we investigated the therapeutic potential of PKC-λ inhibition using ICA-1, a selective small molecule inhibitor that has demonstrated efficacy in various experimental models (Peltonen et al., 2023). ICA-1 has been previously characterized as a potent and selective inhibitor of atypical PKC isoforms, with particular specificity for PKC-λ (McCray et al., 2014). Previous studies have established its anti-proliferative and pro-apoptotic effects in neuroblastoma models, where ICA-1 effectively abrogated cell proliferation and induced apoptosis through inhibition of PKC-λ signaling pathways (Jeschke et al., 2011). Additionally, ICA-1 has been shown to suppress oncogenic epithelial-mesenchymal transition in melanoma by targeting PKC-λ mediated vimentin activation, demonstrating its ability to modulate key cellular processes involved in cancer progression (Ratnayake et al., 2018). Building on this established pharmacological profile, our dose-response studies determined that 5 μM ICA-1 *in vitro* and 50 mg/kg *in vivo* effectively suppressed PKC-λ activation without affecting baseline PKC-λ expression in non-injured controls, indicating a selective therapeutic window. The therapeutic efficacy of ICA-1 in our burn model, demonstrated by significant preservation of muscle mass (restoration to 85.5% of control levels) and functional capacity (restoration to 73.6% of control grip strength), suggests that PKC-λ inhibition could provide clinically meaningful benefits for burn patients. The ability of ICA-1 to suppress multiple downstream catabolic mediators indicates that targeting PKC-λ can effectively interrupt the entire catabolic signaling cascade rather than affecting individual components. The delayed onset of PKC-λ activation provides a potentially viable therapeutic window for early intervention to prevent or attenuate muscle wasting in clinical settings.

In conclusion, this study establishes PKC-λ as a critical mediator of severe burn-induced skeletal muscle atrophy through activation of the STAT3-C/EBPδ catabolic signaling cascade. The selective upregulation of PKC-λ in 3rd versus 2nd burn injury, coupled with the therapeutic efficacy of both genetic and pharmacological PKC-λ inhibition, provides compelling evidence for the clinical potential of targeting this pathway. These findings represent a significant advance in understanding the molecular mechanisms underlying burn-induced muscle wasting and offer a promising therapeutic approach for improving outcomes in this challenging patient population. However, several limitations should be acknowledged. The sample size of human burn patients was relatively small (n = 5 per group), which may limit the generalizability of our findings across diverse patient populations. Our analysis focused specifically on the vastus lateralis muscle, and it remains unclear whether PKC-λ activation patterns are consistent across different muscle groups with varying fiber type compositions and metabolic profiles. Additionally, while our studies demonstrate the therapeutic efficacy of PKC-λ inhibition in preventing muscle atrophy, the potential for this approach to reverse established muscle wasting remains unexplored, which would be particularly relevant for clinical applications where patients may present with varying degrees of pre-existing muscle loss. Furthermore, we did not perform direct PKC-λ kinase activity assays. The lack of a STAT3 overexpression alone group in Figure 3 limits the gain-of-function evidence; however, the Co-IP data and pharmacological rescue strongly support the hierarchical relationship. Additionally, we did not examine other PKC isoforms such as PKCε, which may play roles in muscle regeneration (Di Marcantonio et al., 2015; Pozzi et al., 2023). Future studies should include larger patient cohorts, examine multiple muscle groups, and investigate the potential for PKC-λ inhibition as both a preventive and therapeutic intervention in established muscle wasting.

## Data Availability

The raw data supporting the conclusions of this article will be made available by the authors, without undue reservation.
