# Supplementary material for: Protein kinase C-λ drives third-degree burn-induced muscle wasting via STAT3-dependent catabolic pathways
Source: Front Pharmacol. 2026 Sep 3;17:1839235. doi: 10.3389/fphar.2026.1839235 (PMC13584706; doi:10.3389/fphar.2026.1839235)

Fig 1A PKC-α.jpg

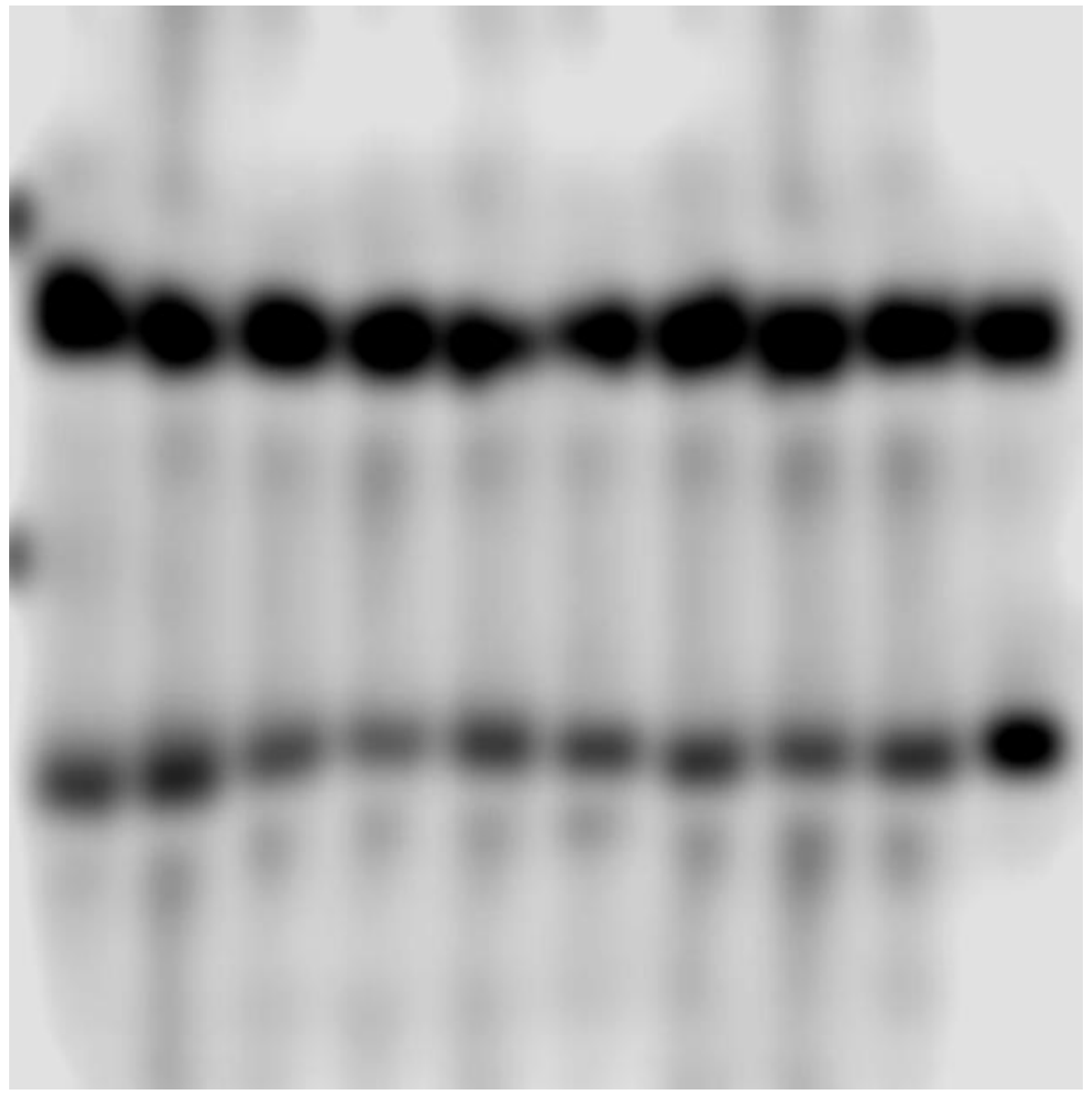

Fig 1A PKC-β.jpg

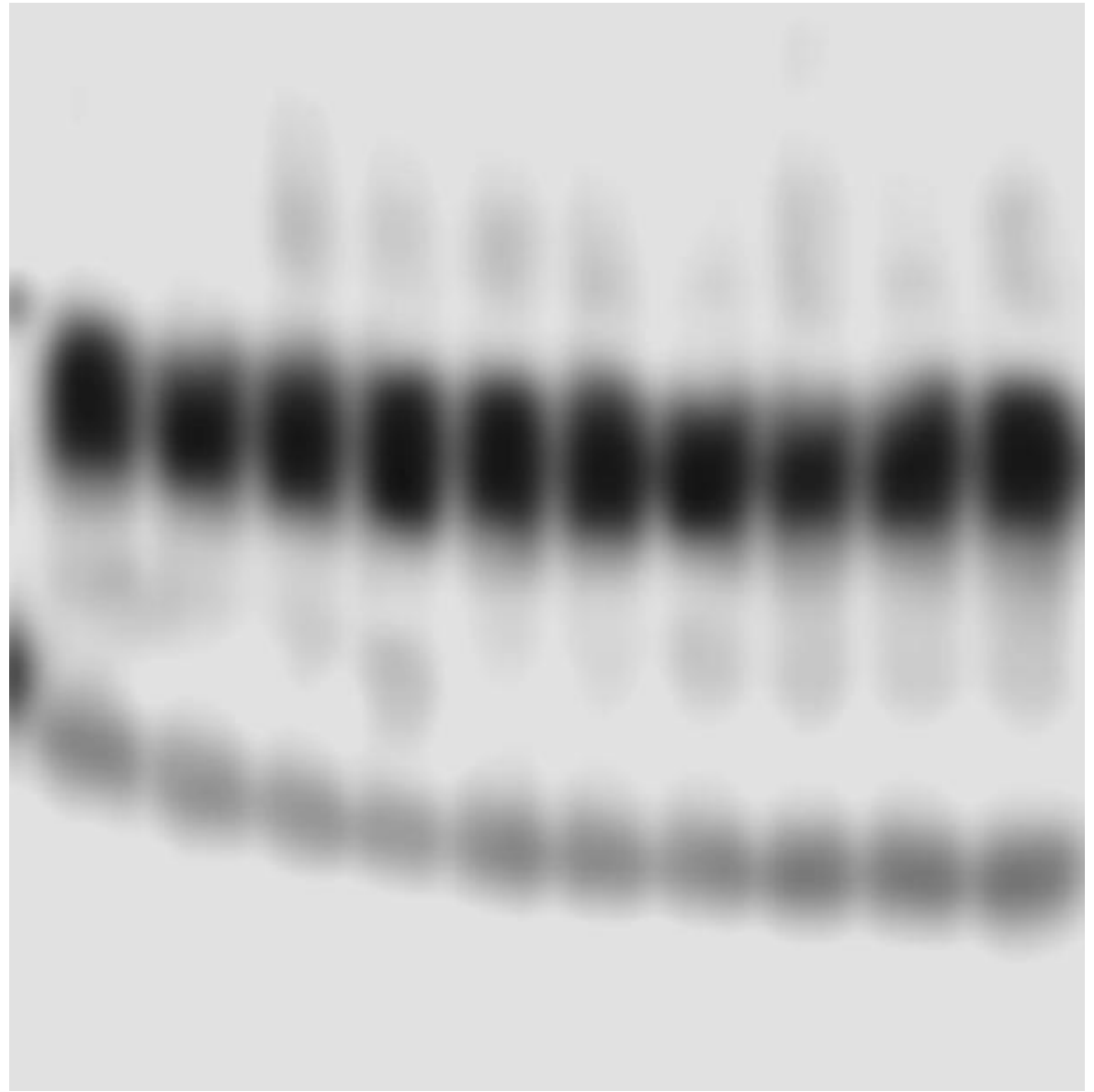

Fig 1A p-PKC-α.jpg

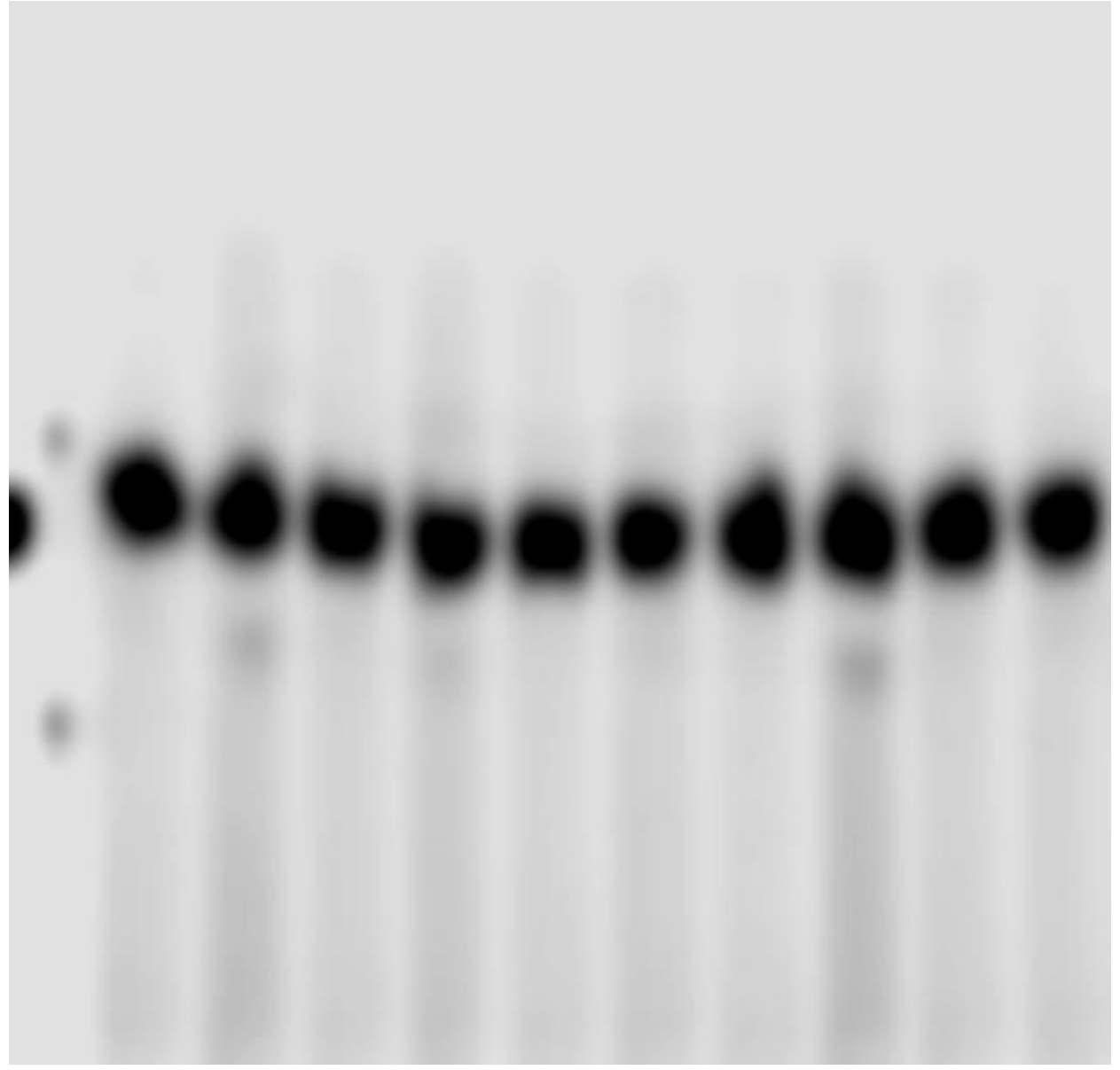

Fig 1A p-PKC-β.jpg

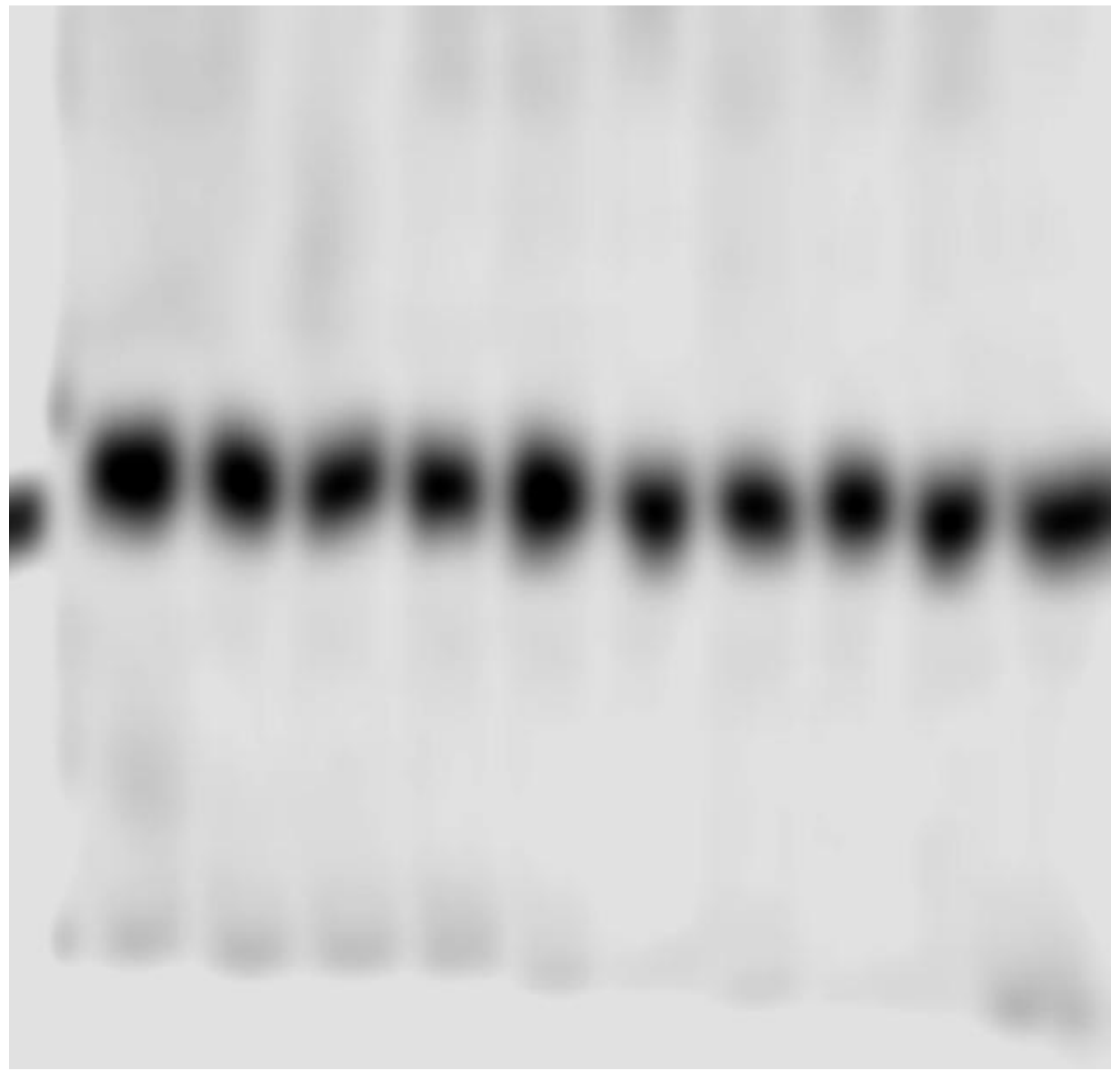

Fig 1A β-tubulin.jpg

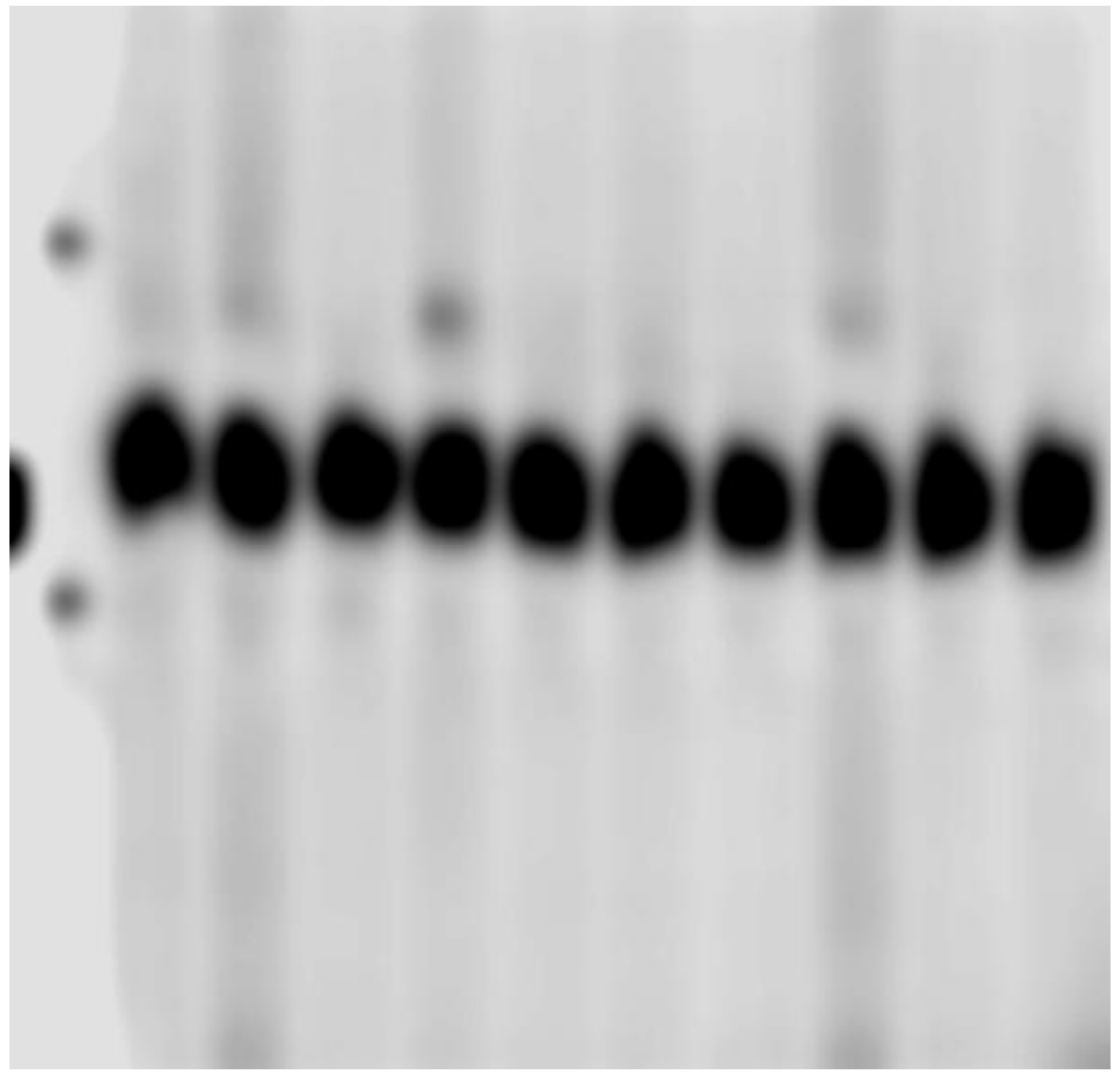

Fig 1B PKC-α.jpg

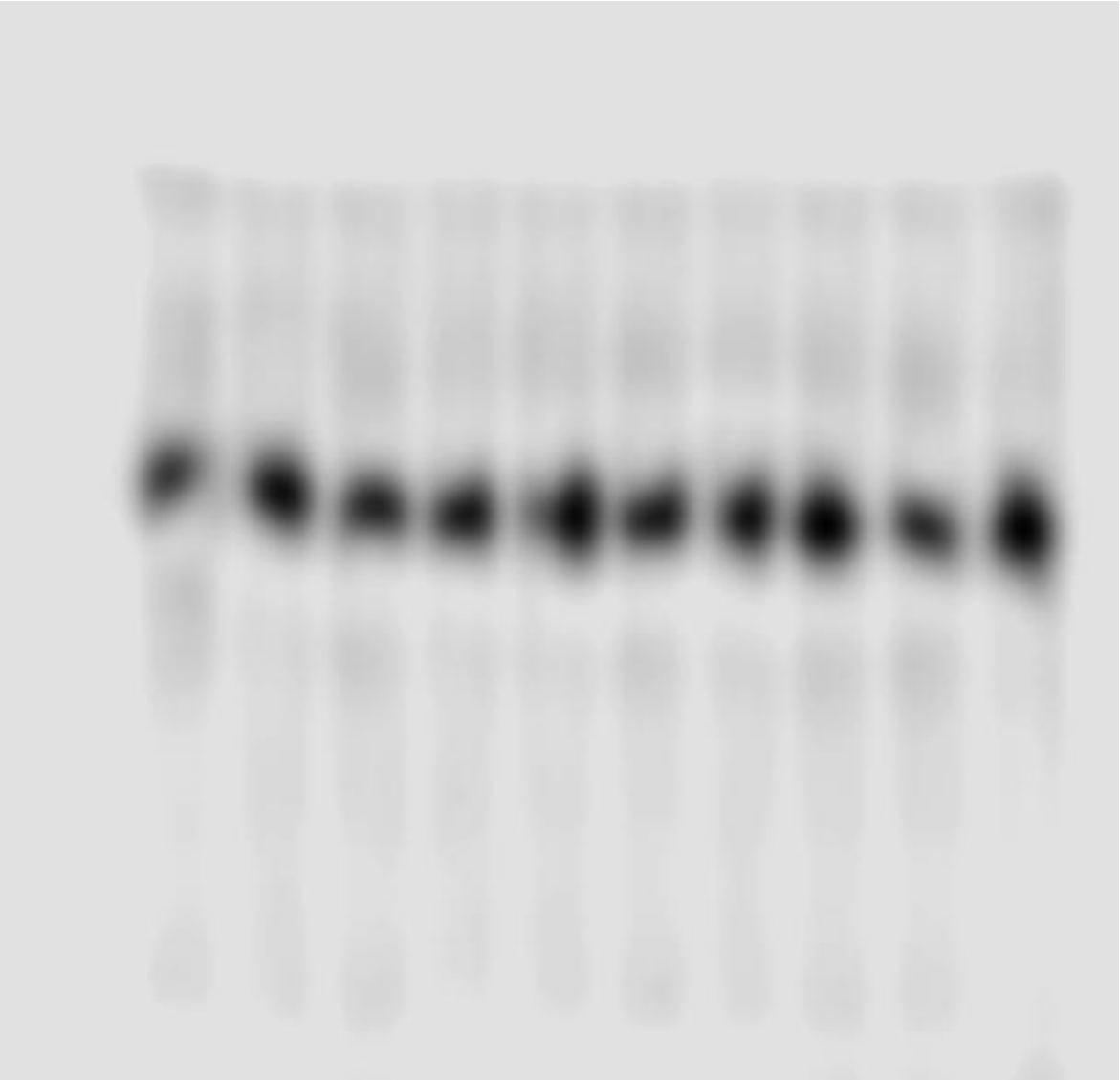

Fig 1B PKC-β.jpg

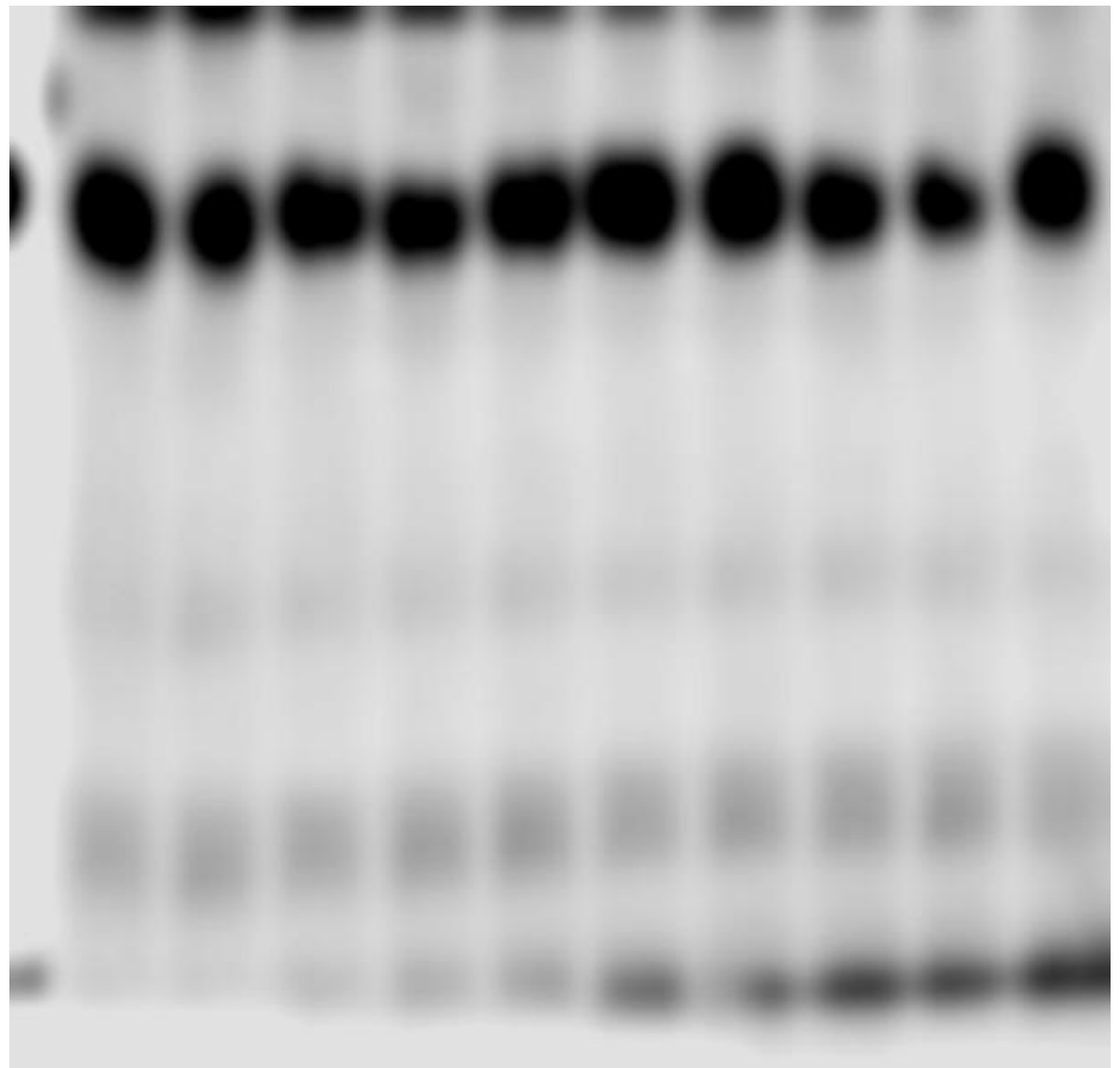

Fig 1B p-PKC-α.jpg

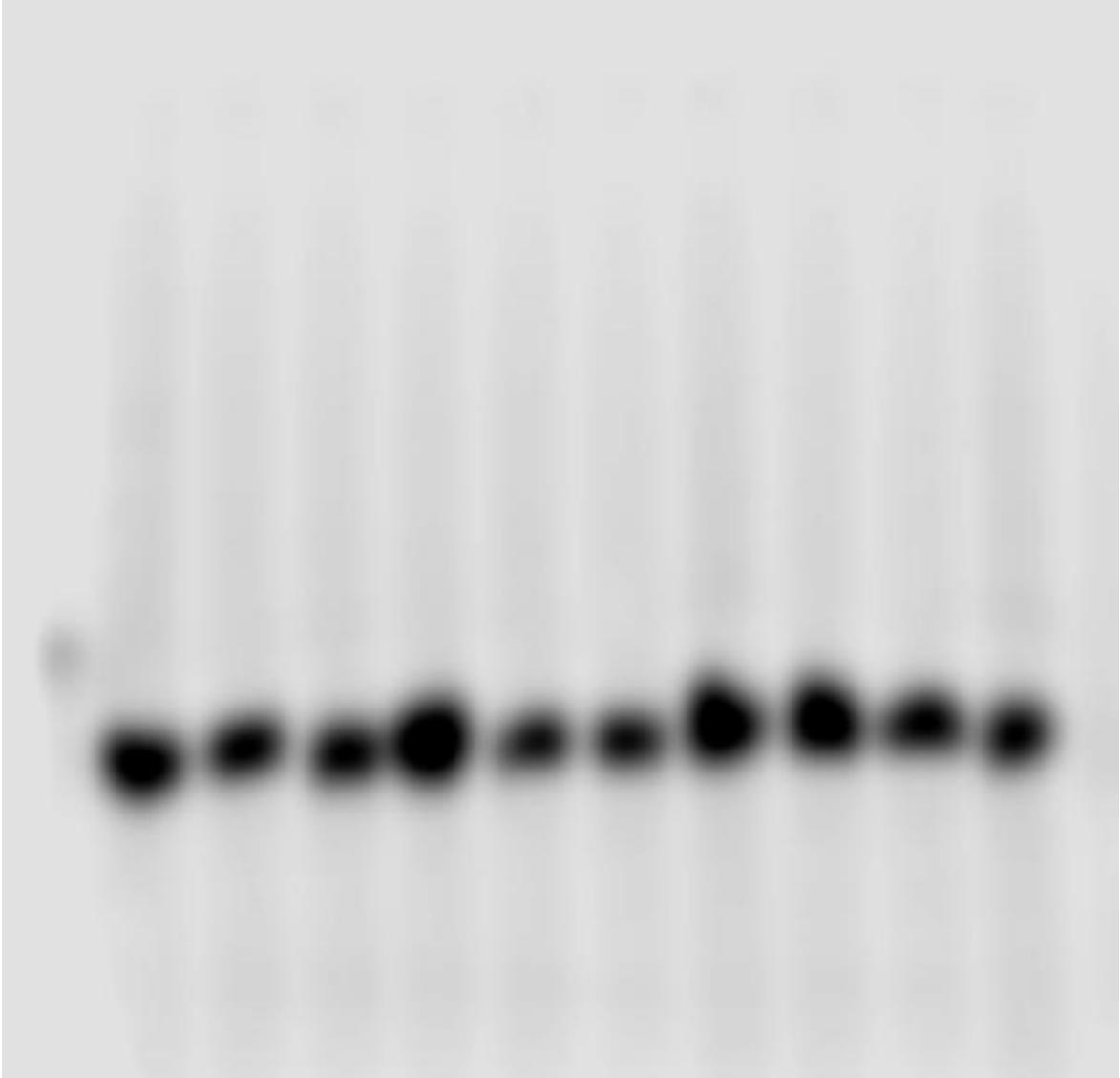

Fig 1B p-PKC-β.jpg

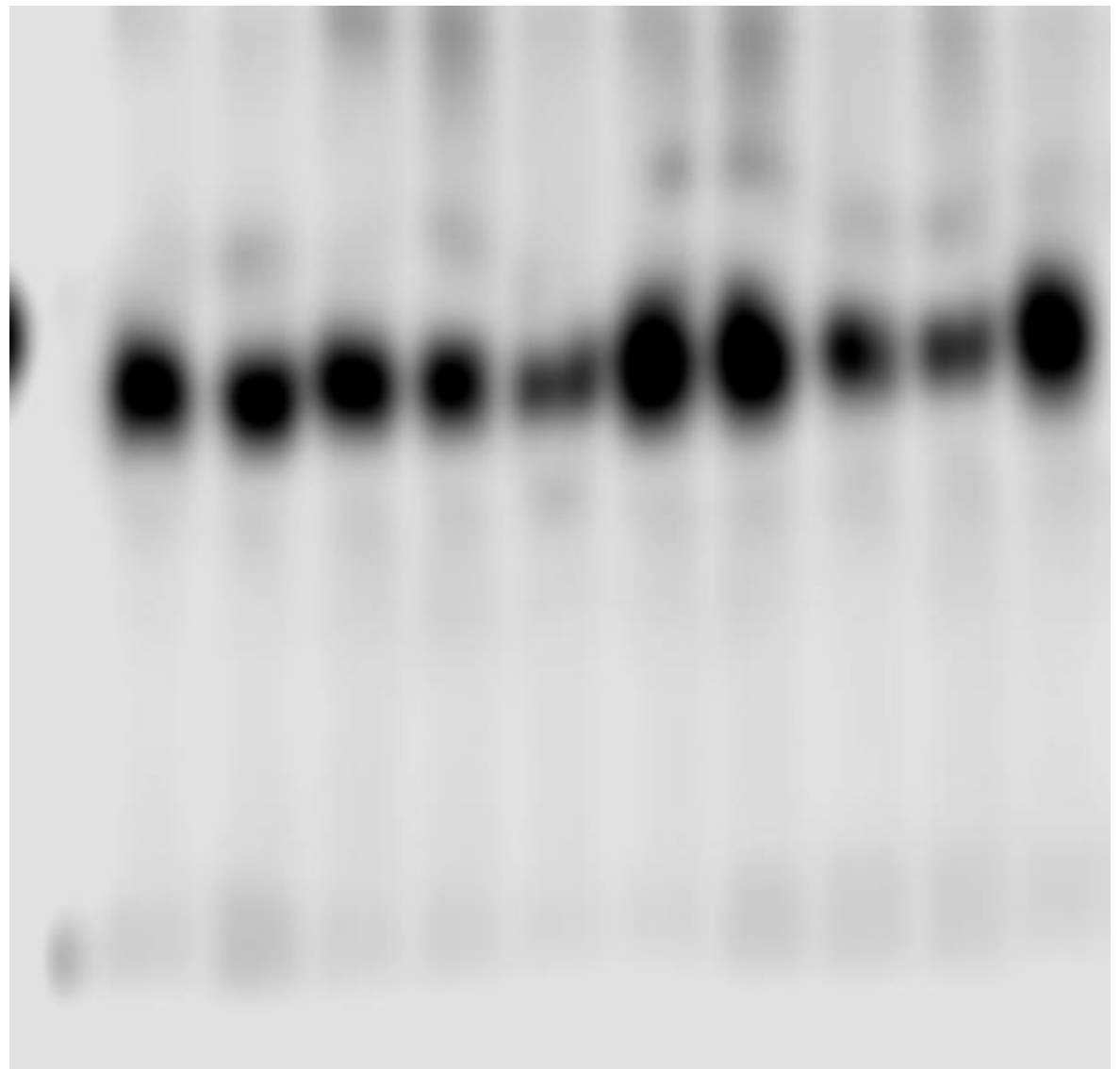

Fig 1B β-tubulin.jpg

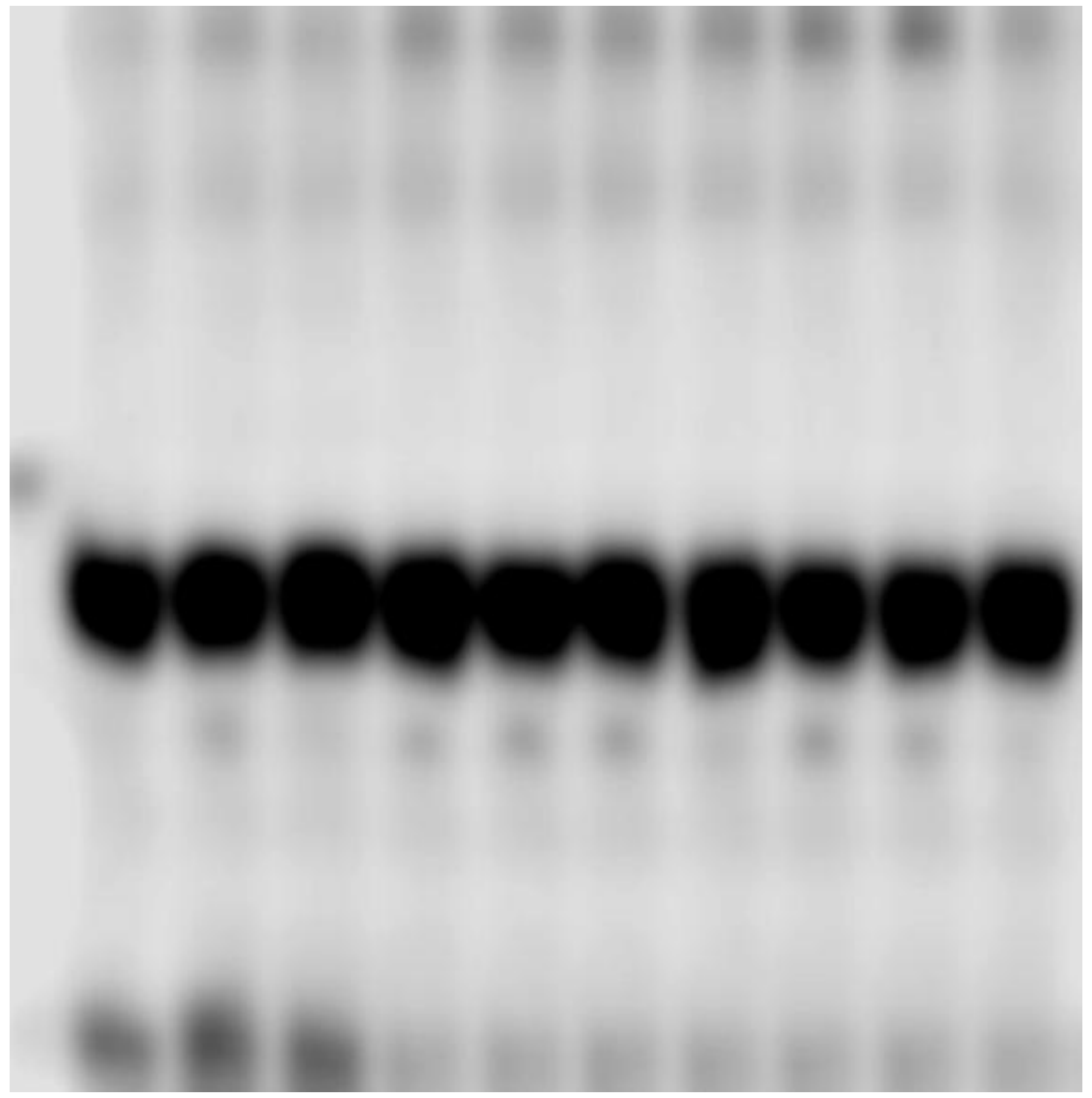

Fig 1C β-tubulin.jpg

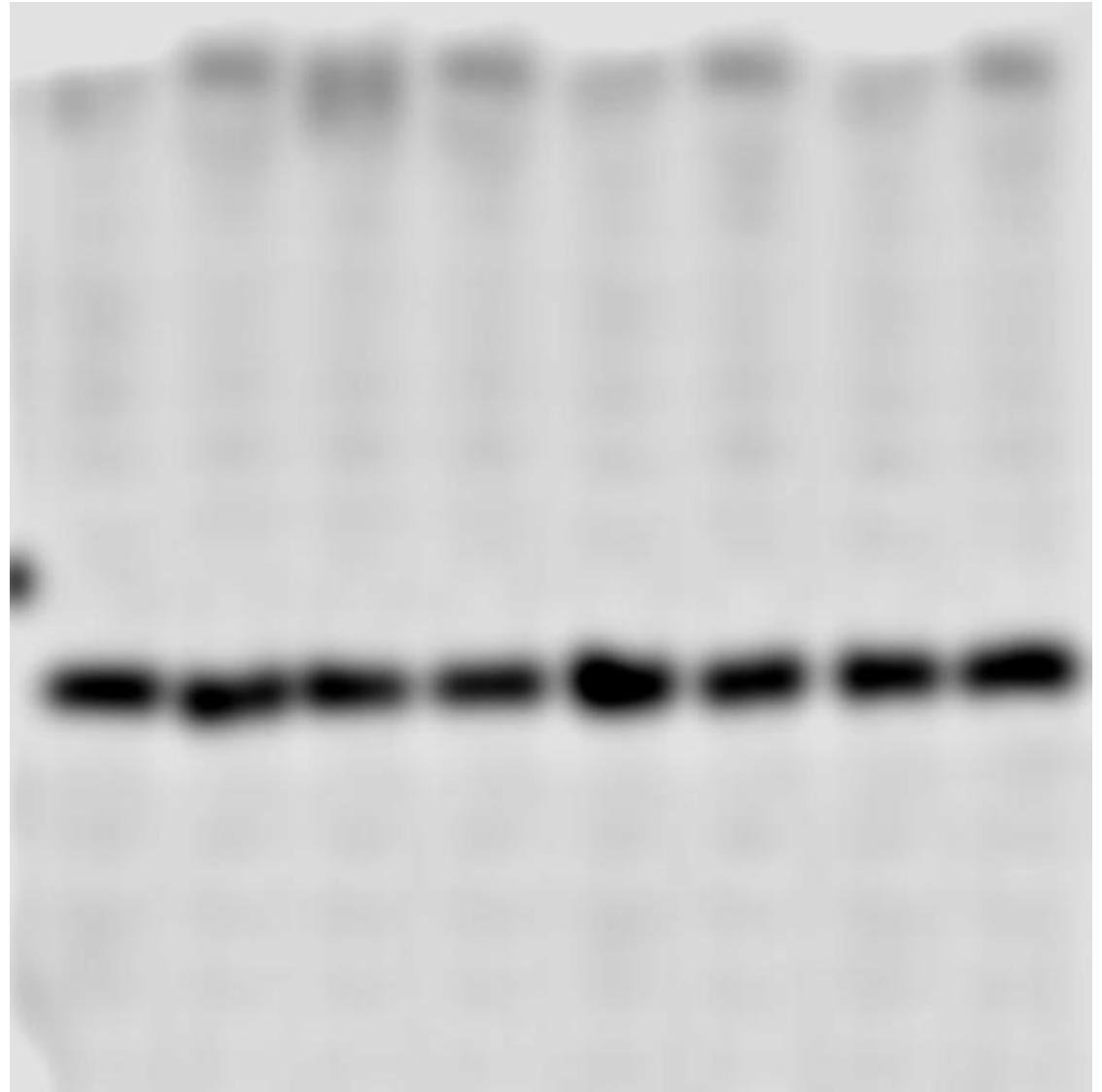

Fig1A PKC-δ.jpg

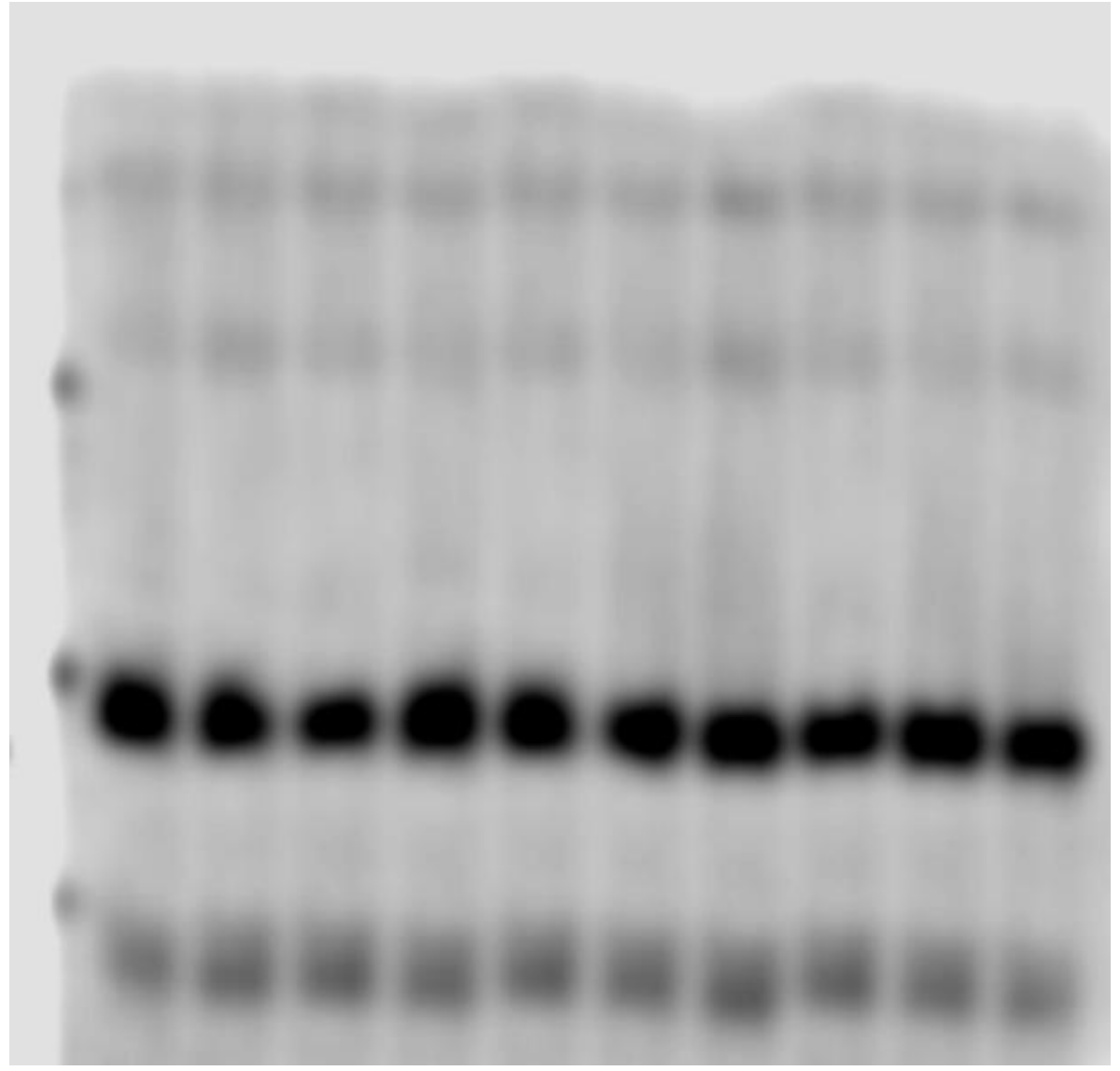

Fig1A PKC-ζ .jpg

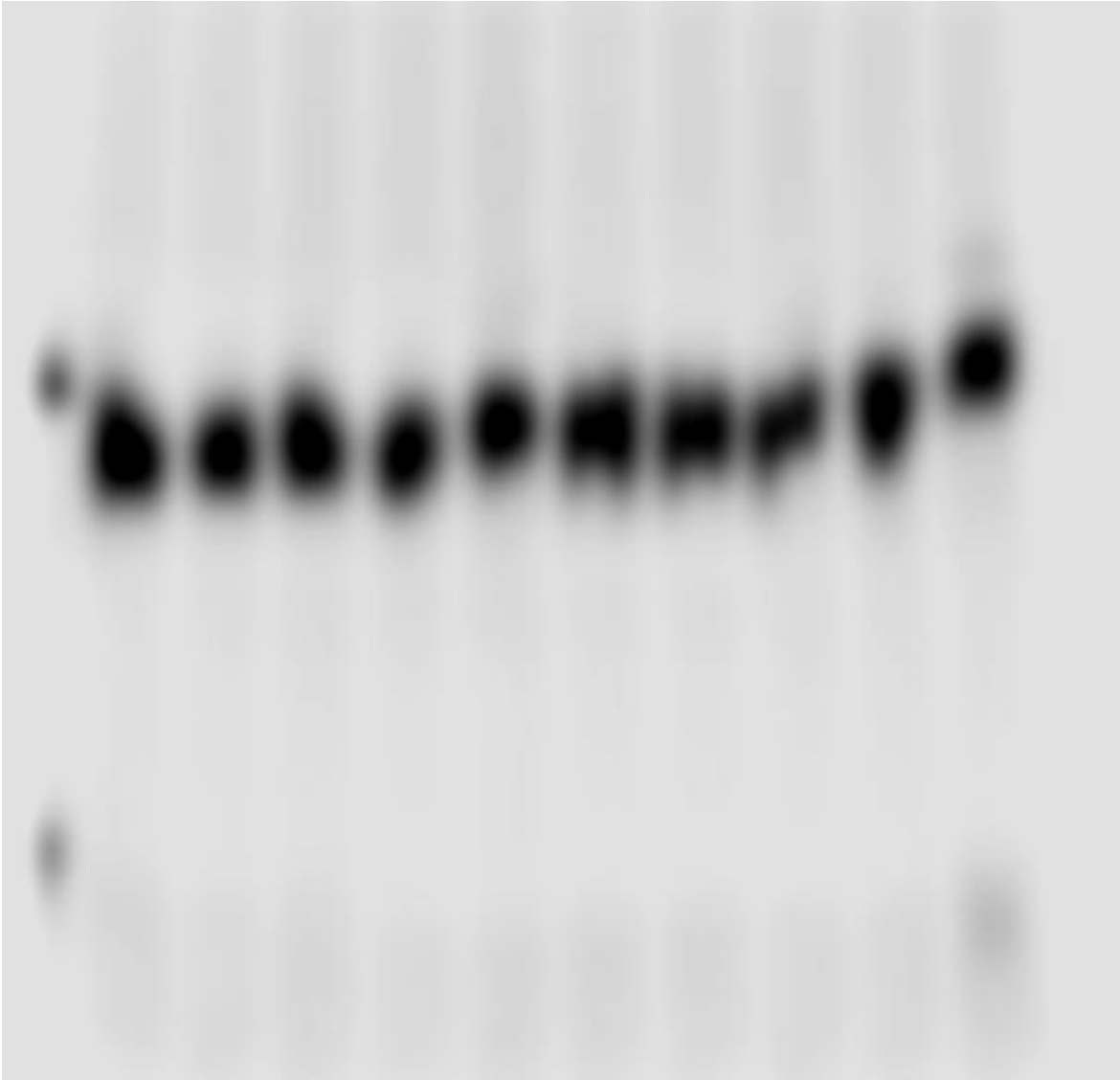

Fig 1B PKC-α.jpg

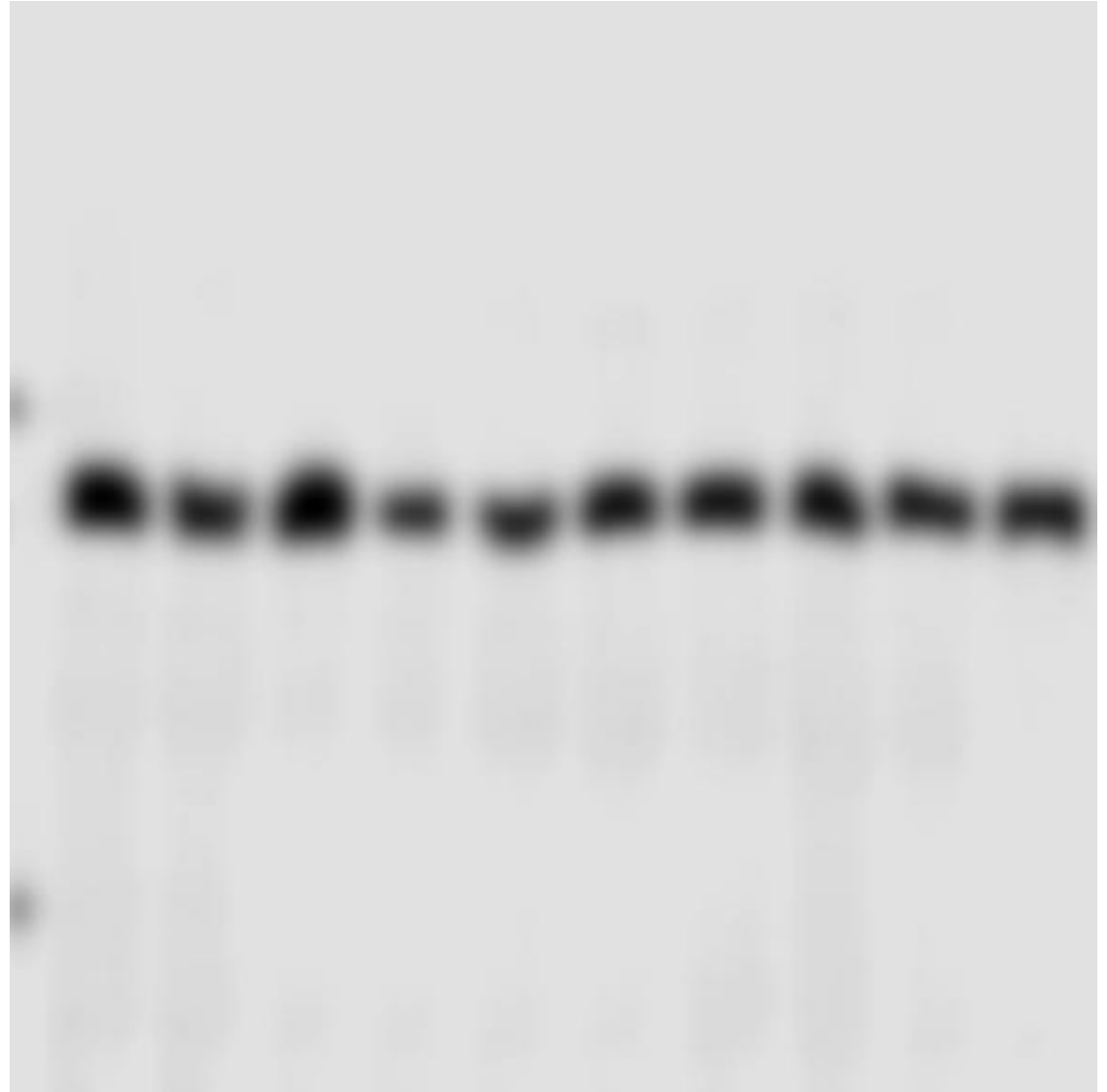

Fig1A PKC-λ

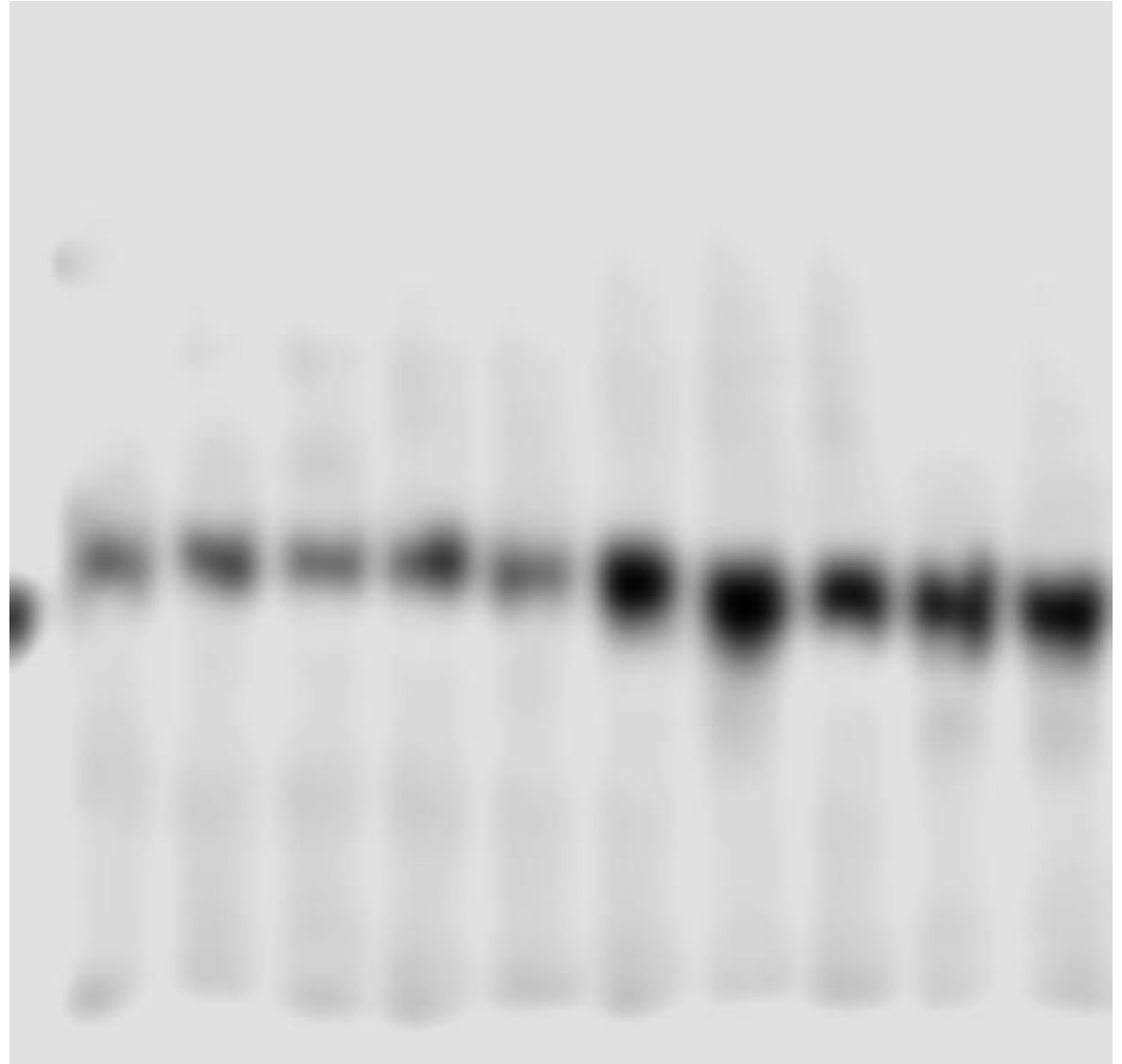

Fig1A p-PKC-δ

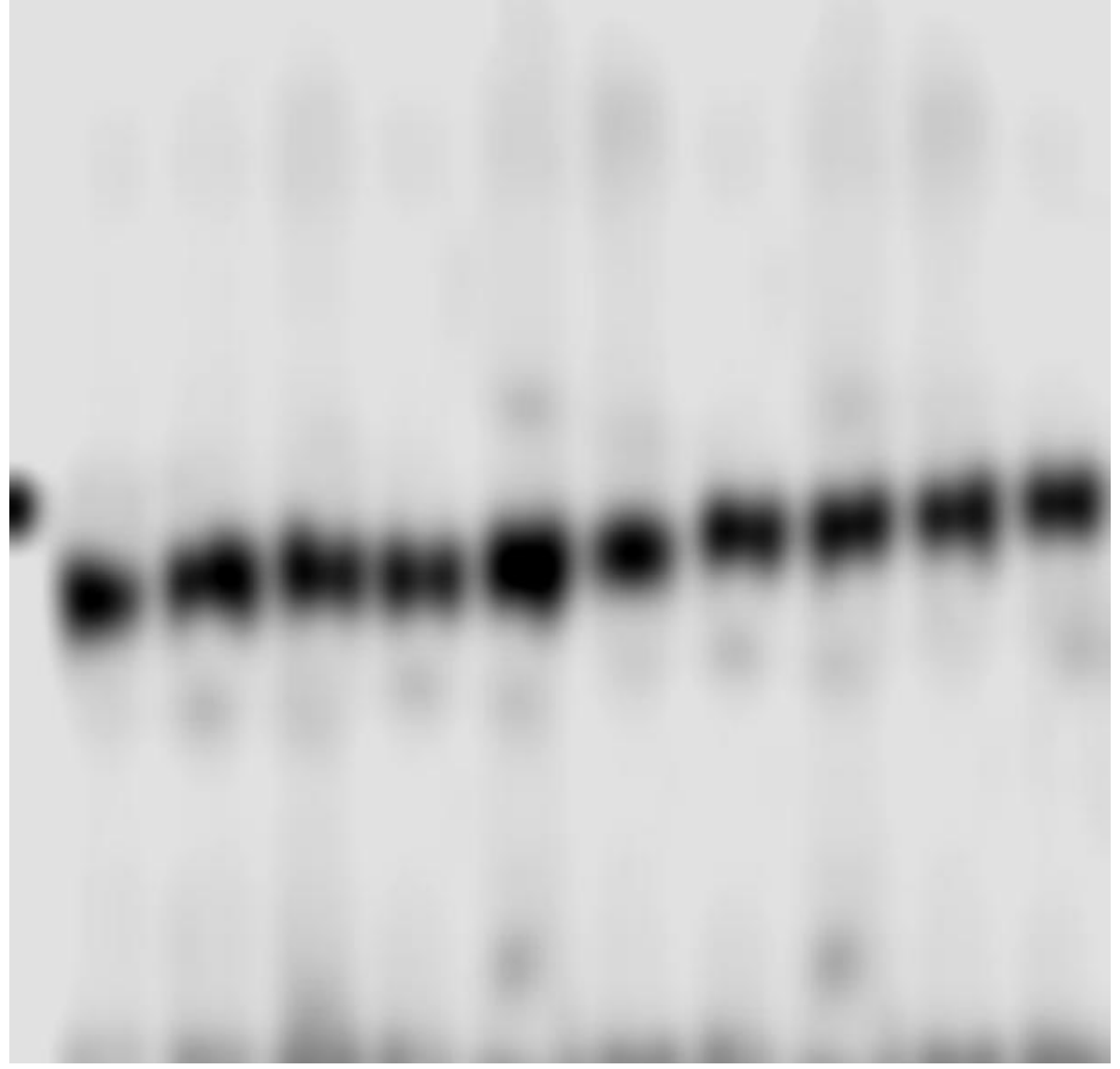

Fig1A p-PKC-ζ.jpg

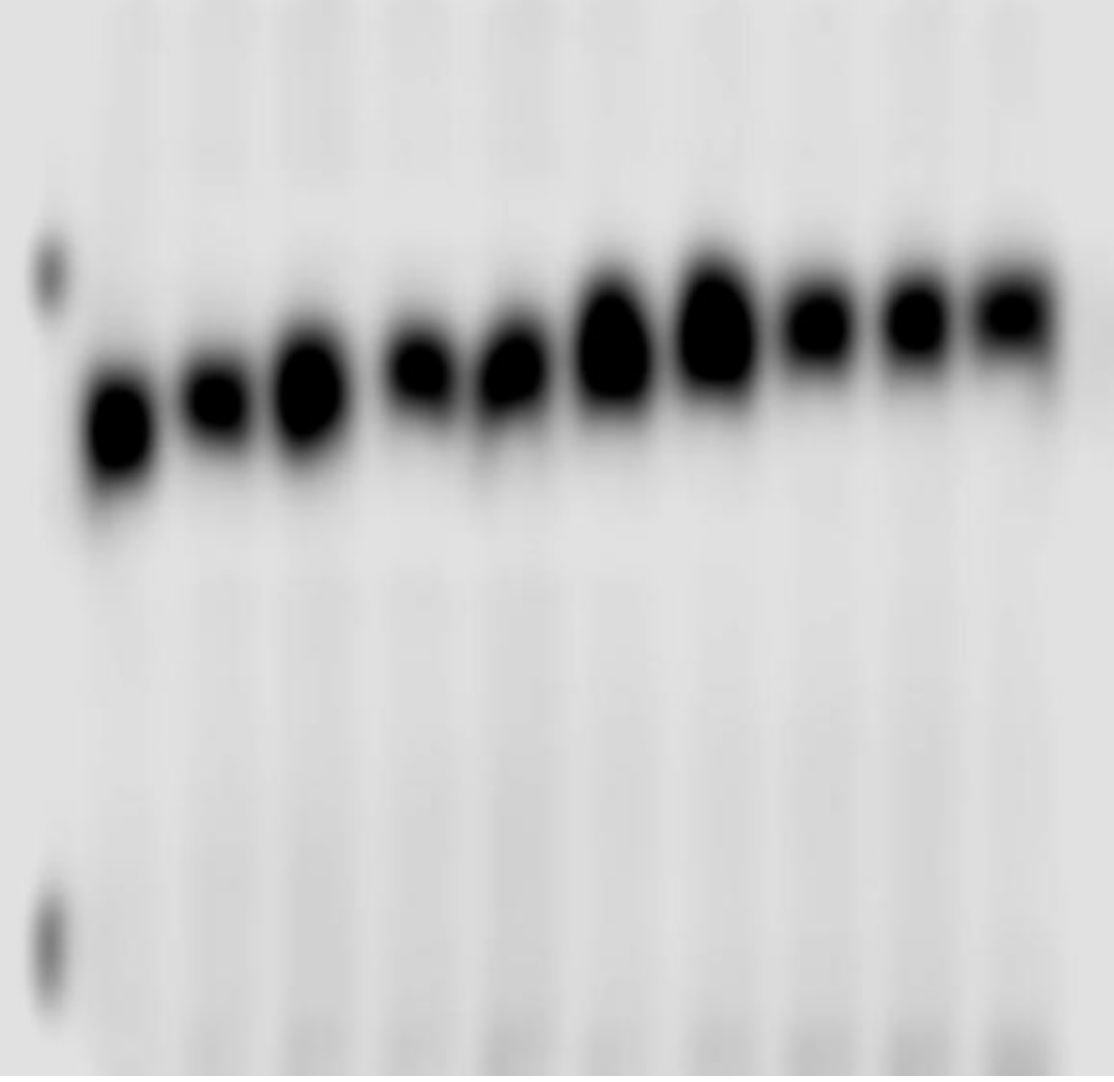

Fig1A p-PKC-θ.jpg

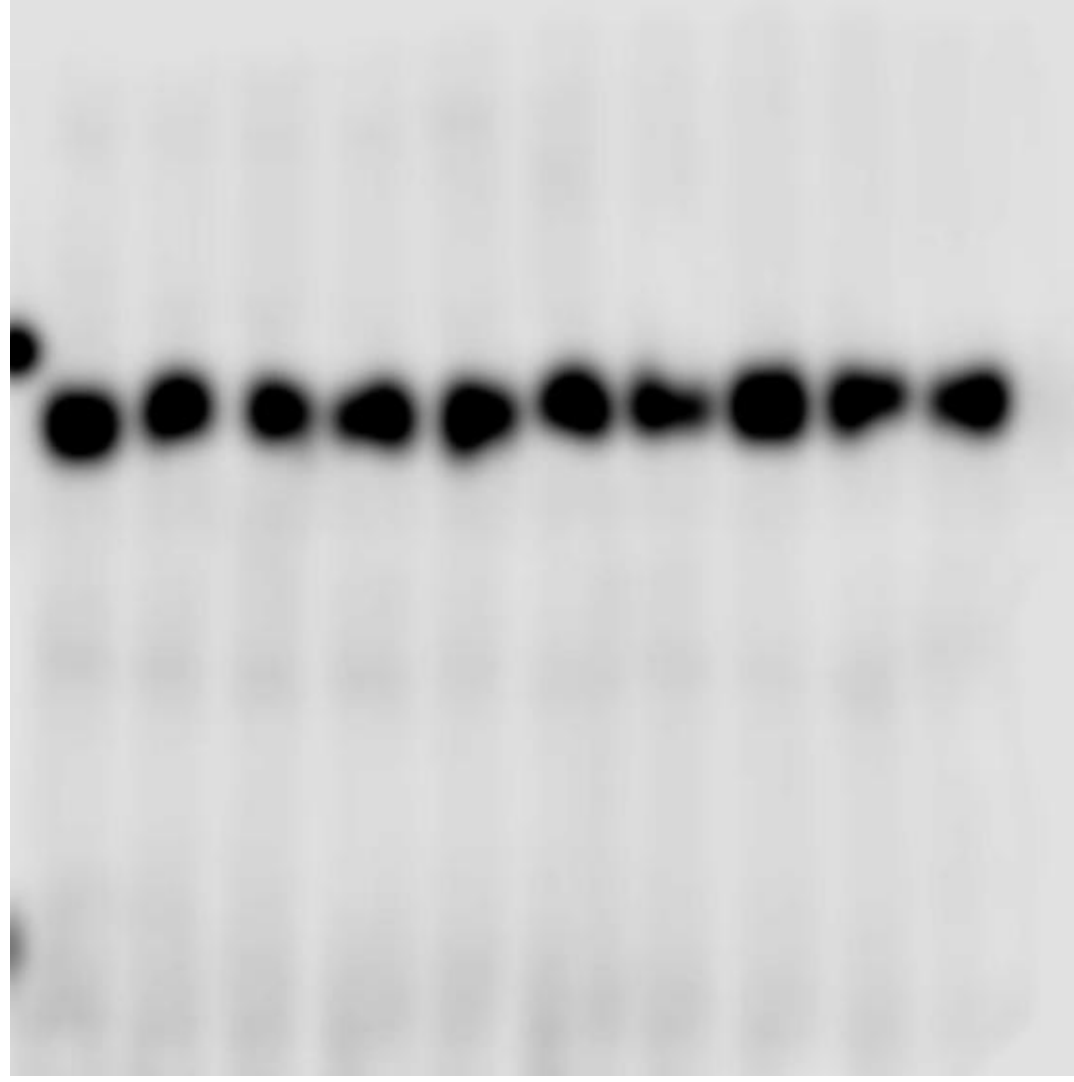

Fig1A p-PKC-λ.jpg

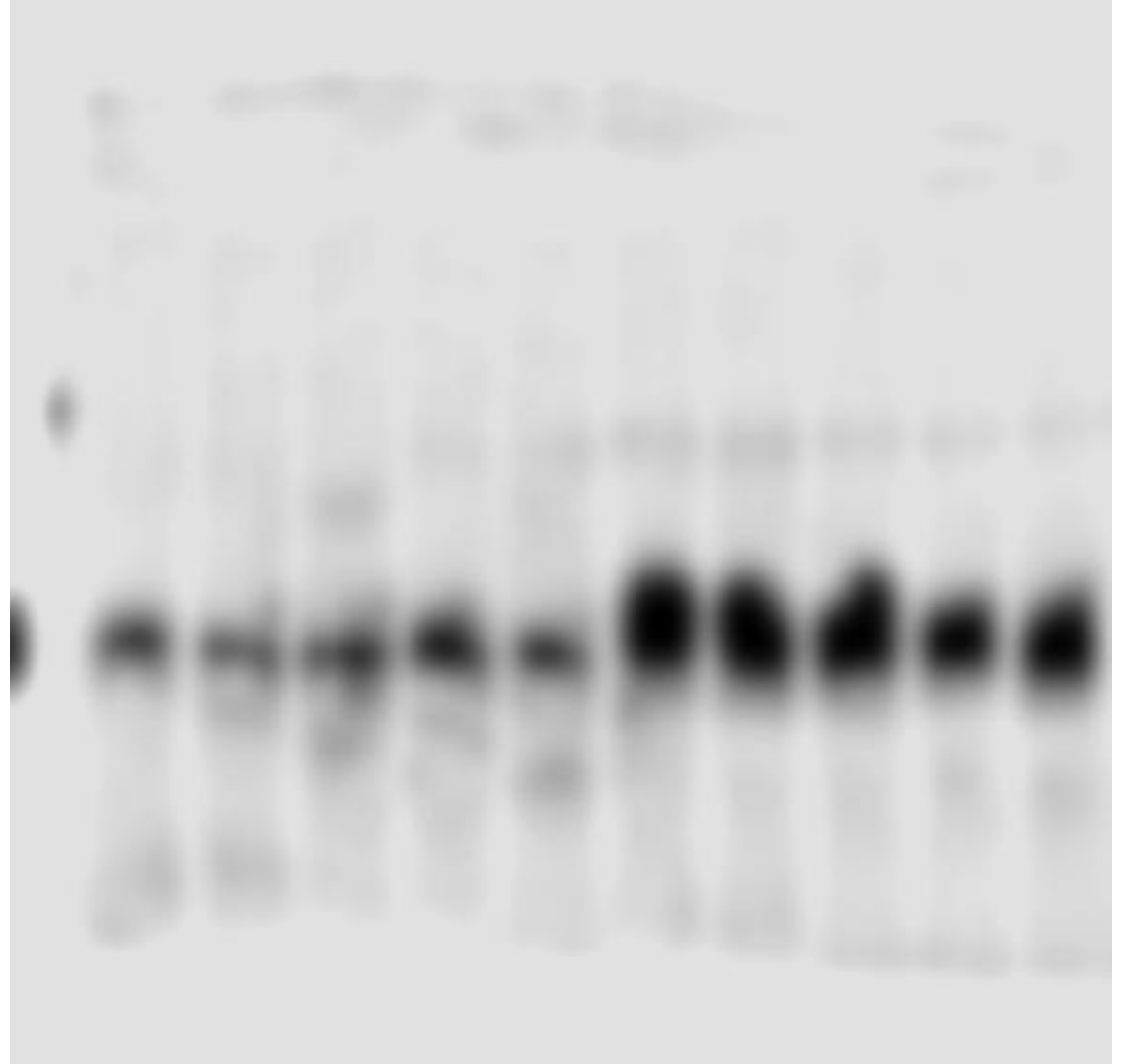

Fig1B PKC-δ.jpg

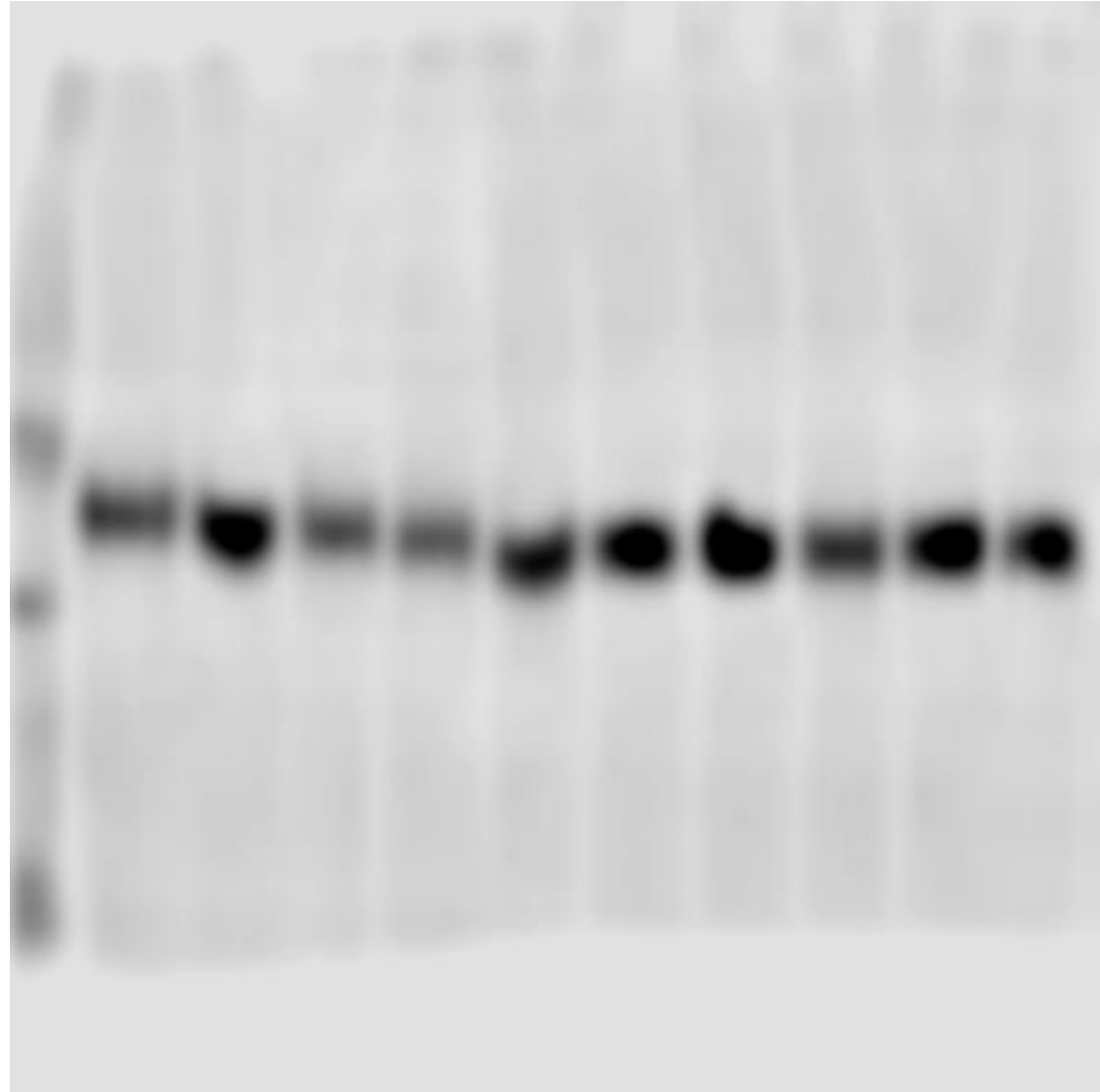

Fig1B PKC-ζ.jpg

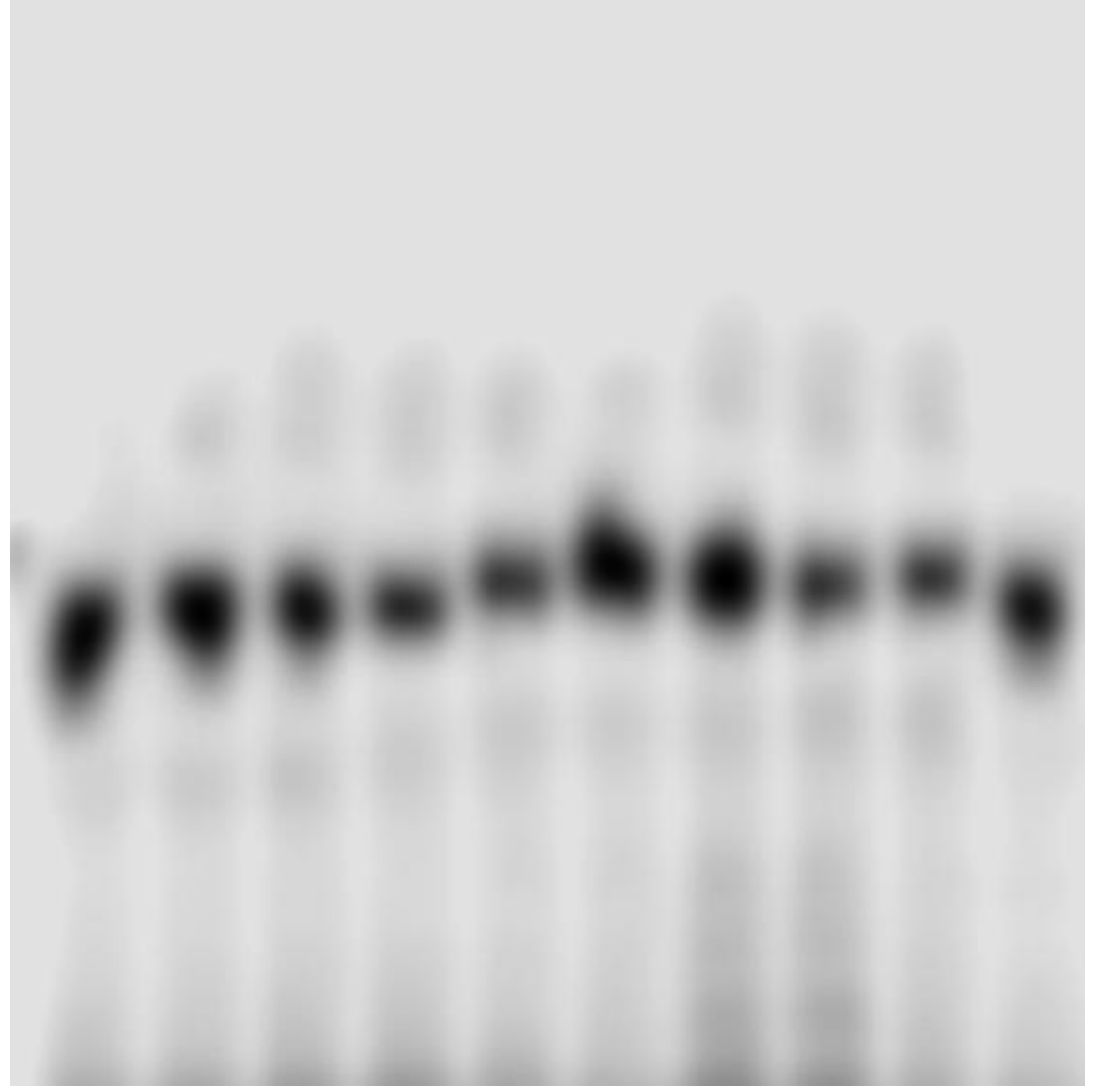

Fig1B PKC-θ.jpg

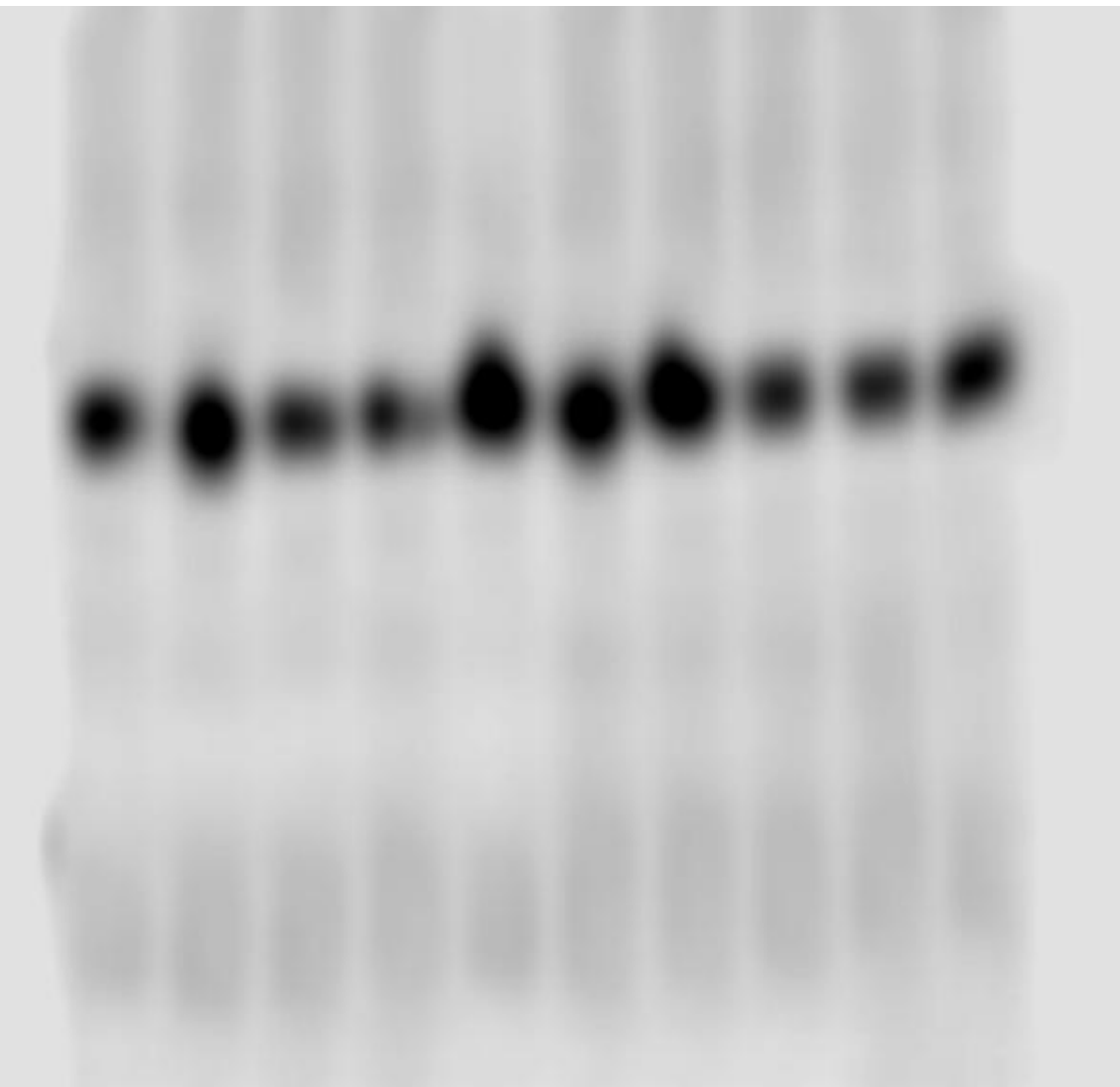

Fig1B PKC-λ.jpg

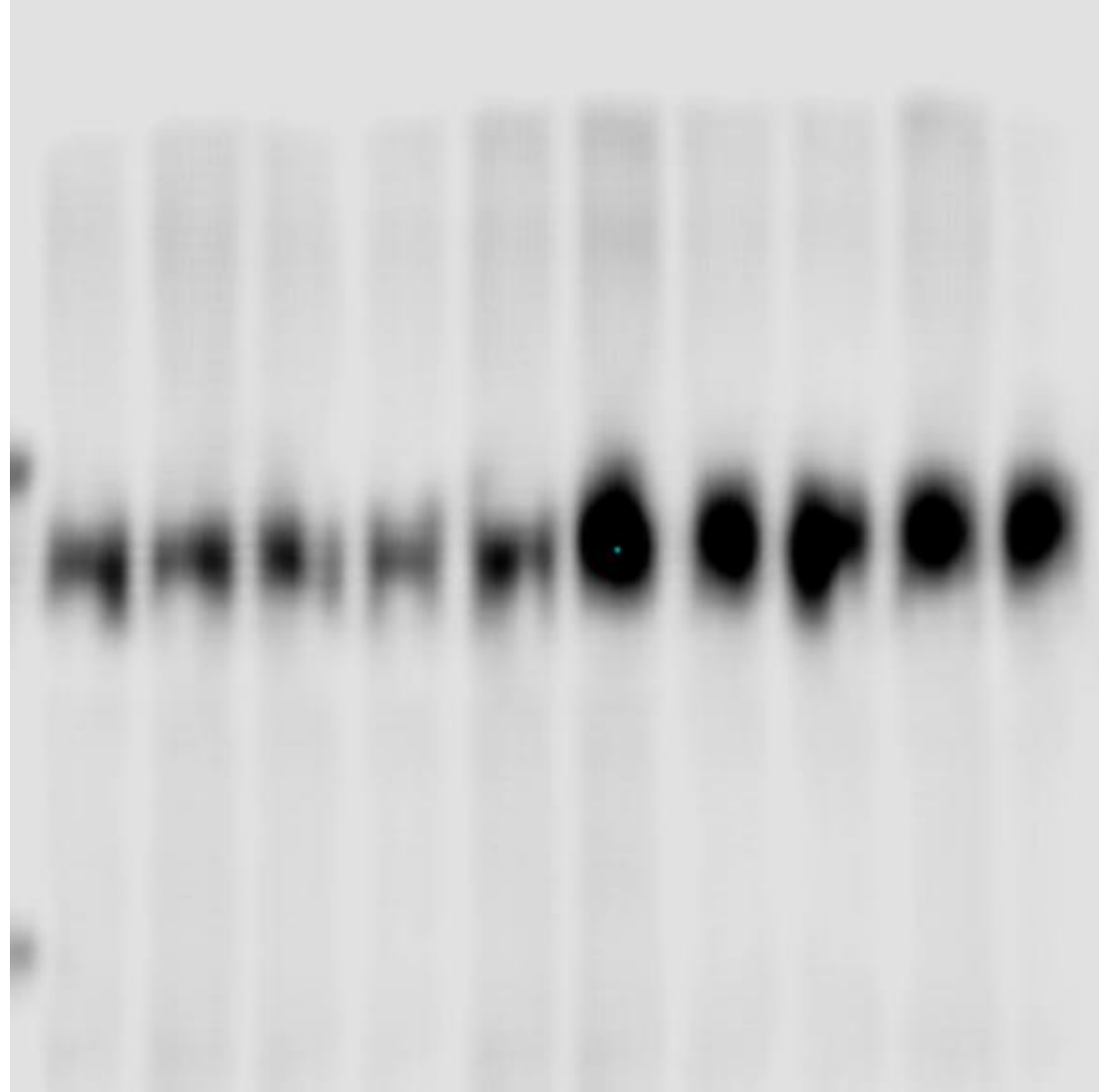

Fig1B p-PKC-δ.jpg

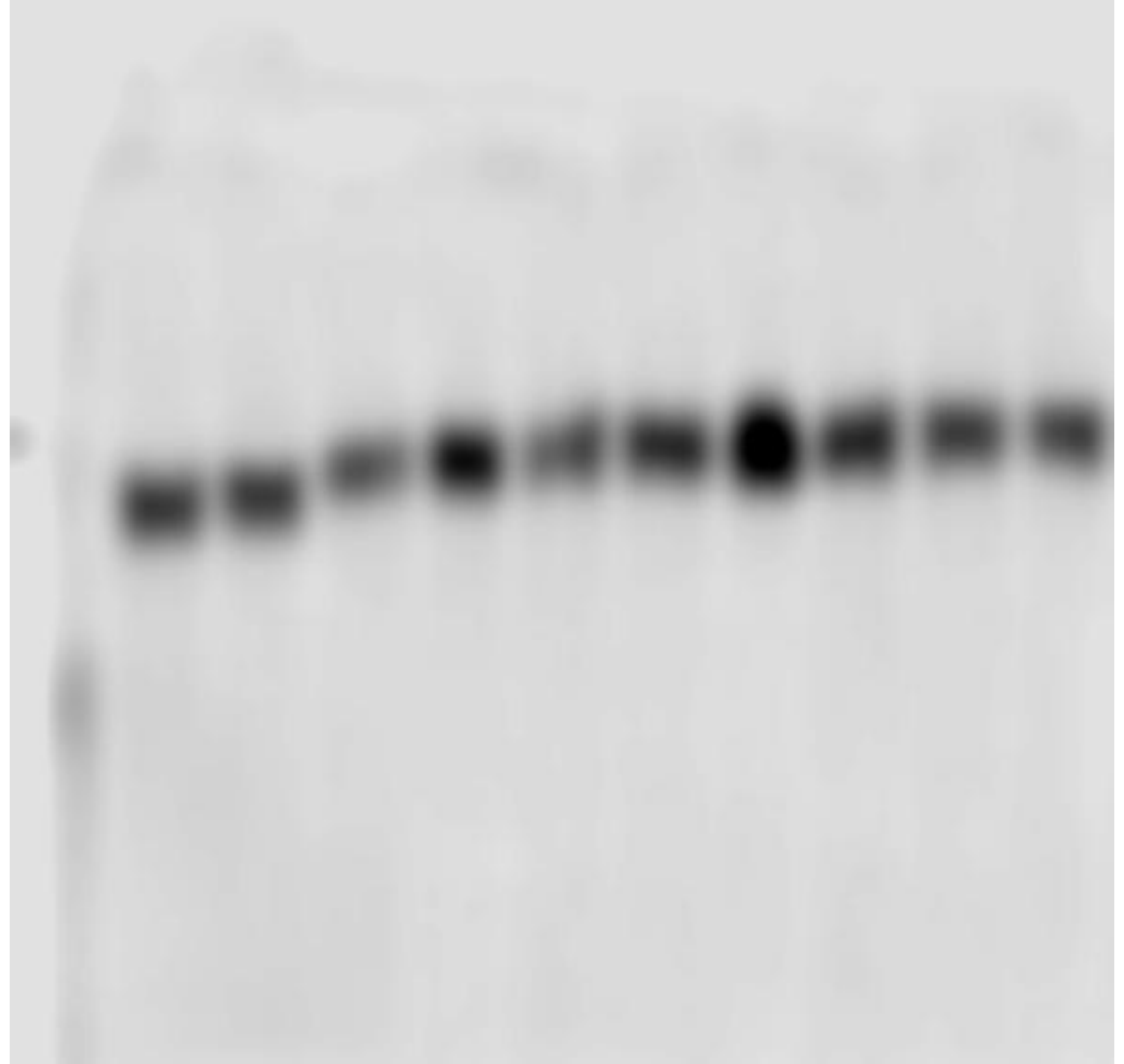

Fig1B p-PKC-ζ.jpg

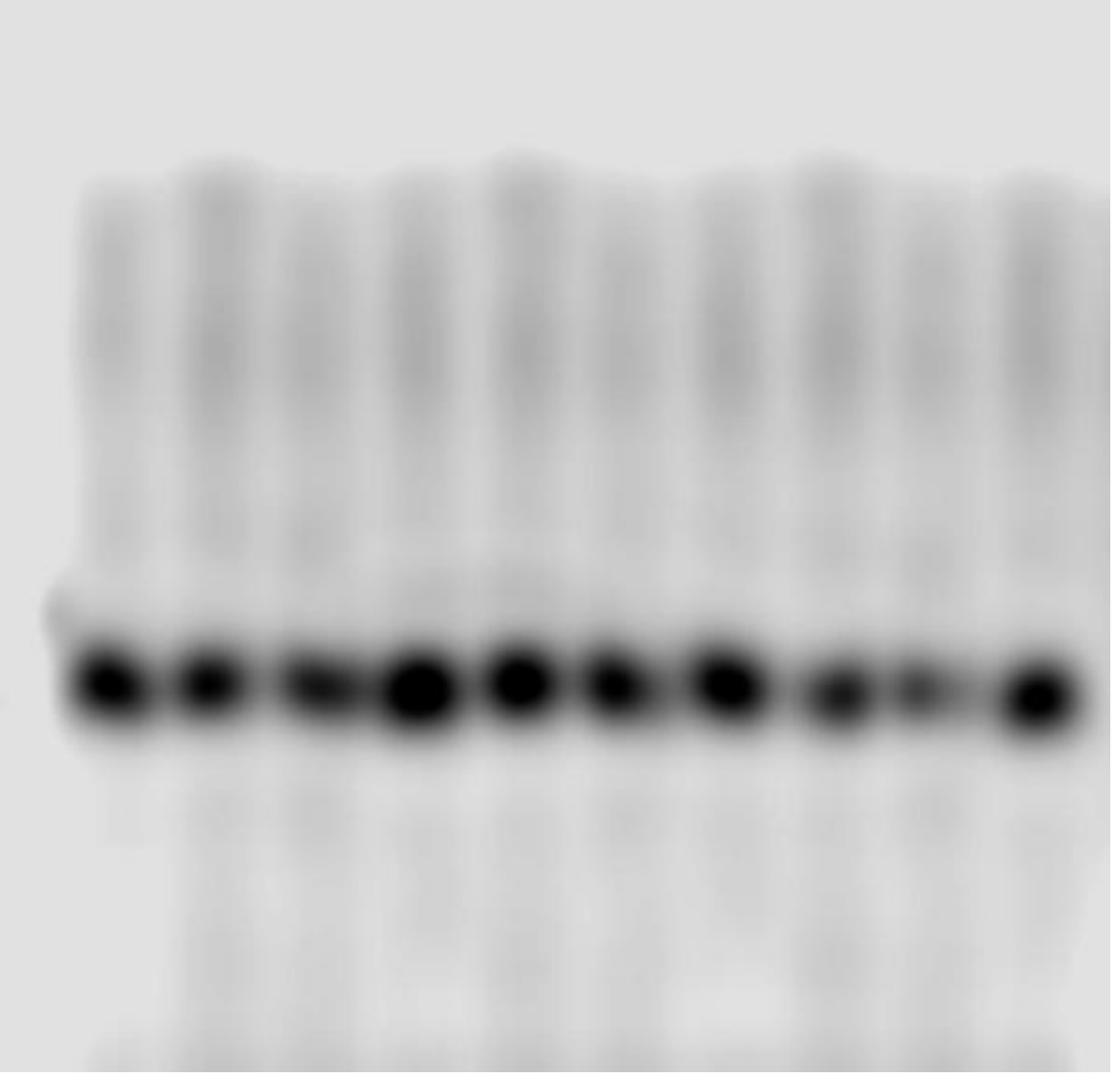

Fig1B p-PKC-θ.jpg

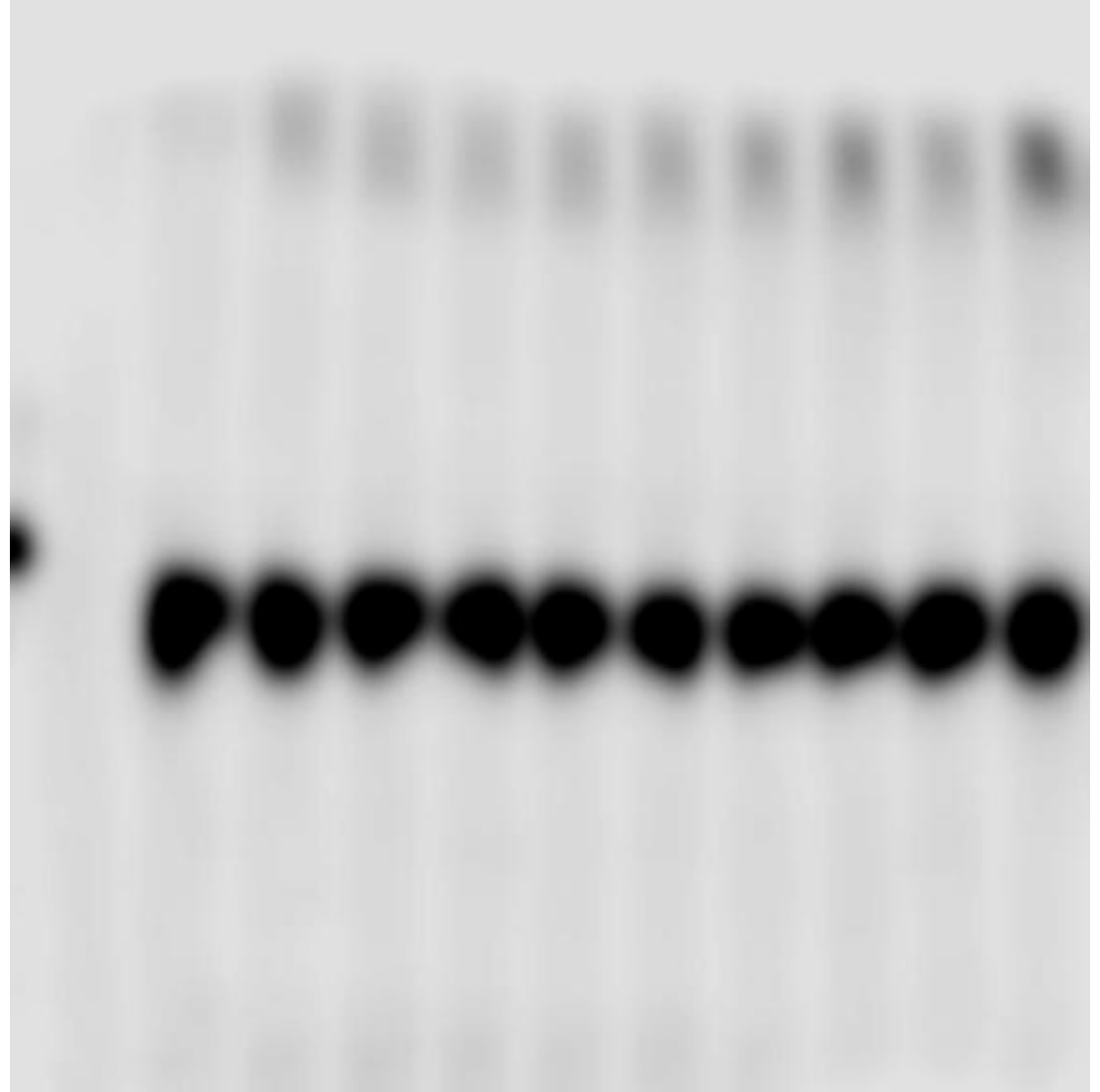

Fig1B p-PKC-λ .jpg

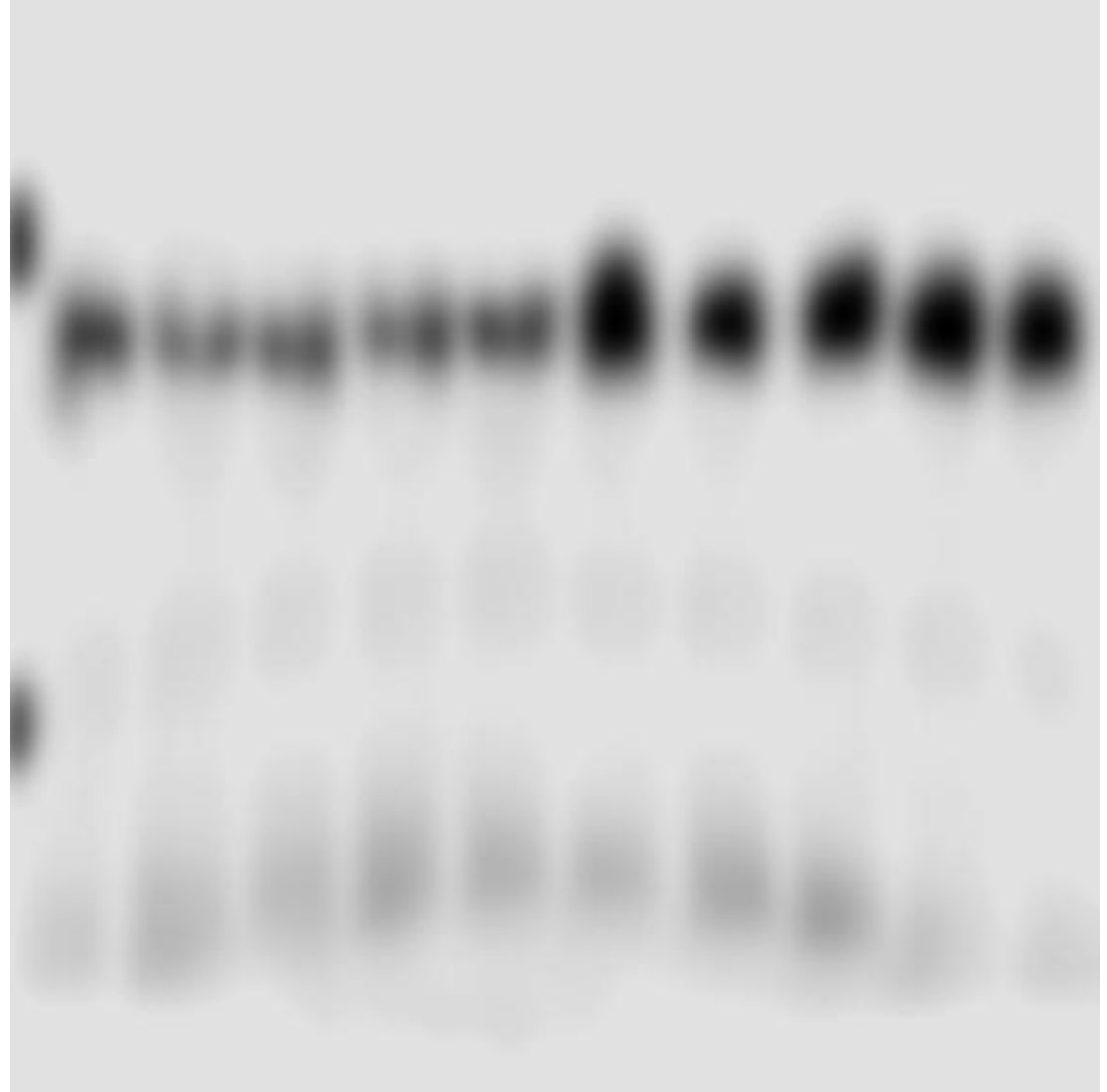

Fig1C PKC-λ

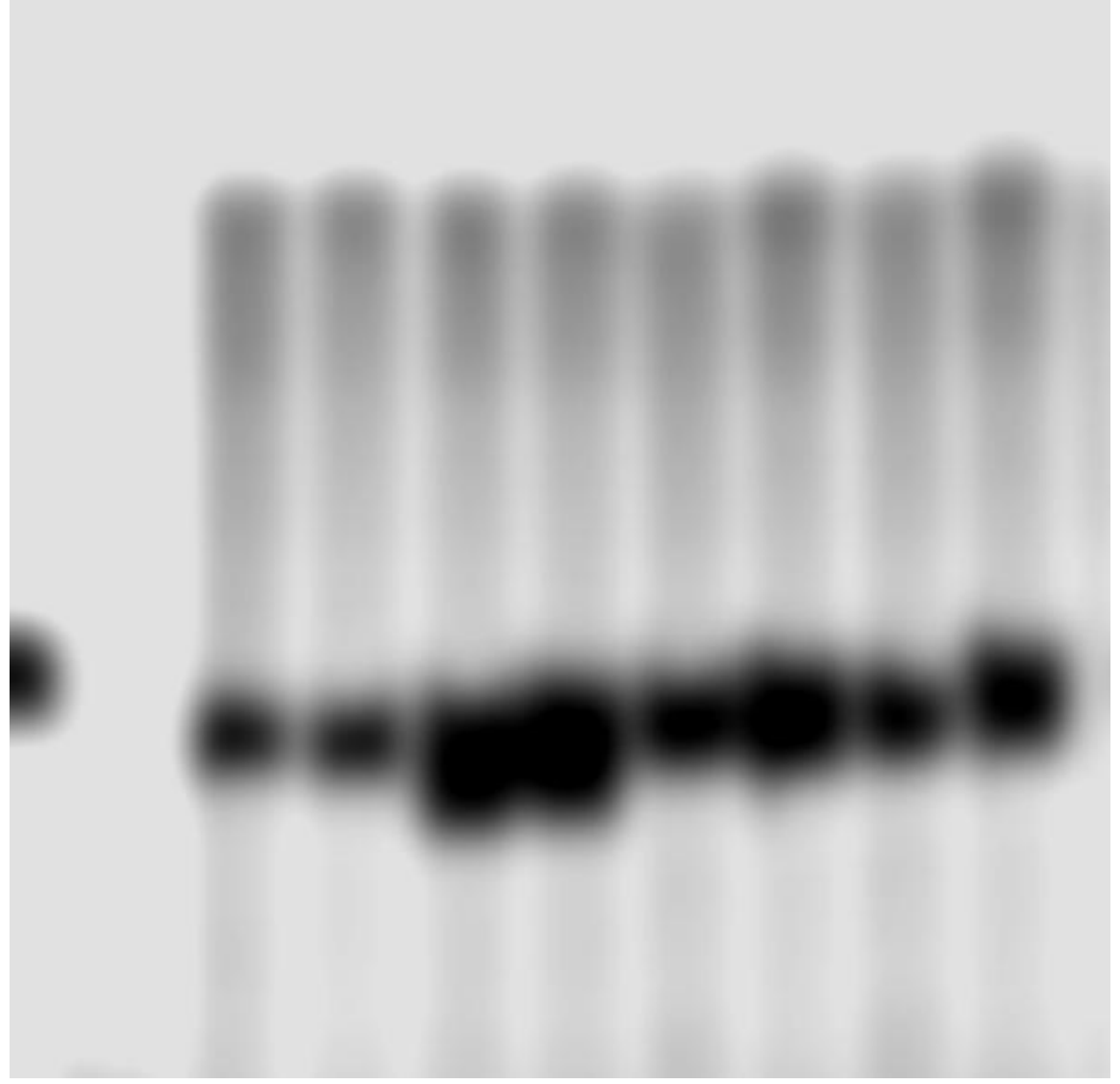

Fig1C p-PKC-λ.jpg

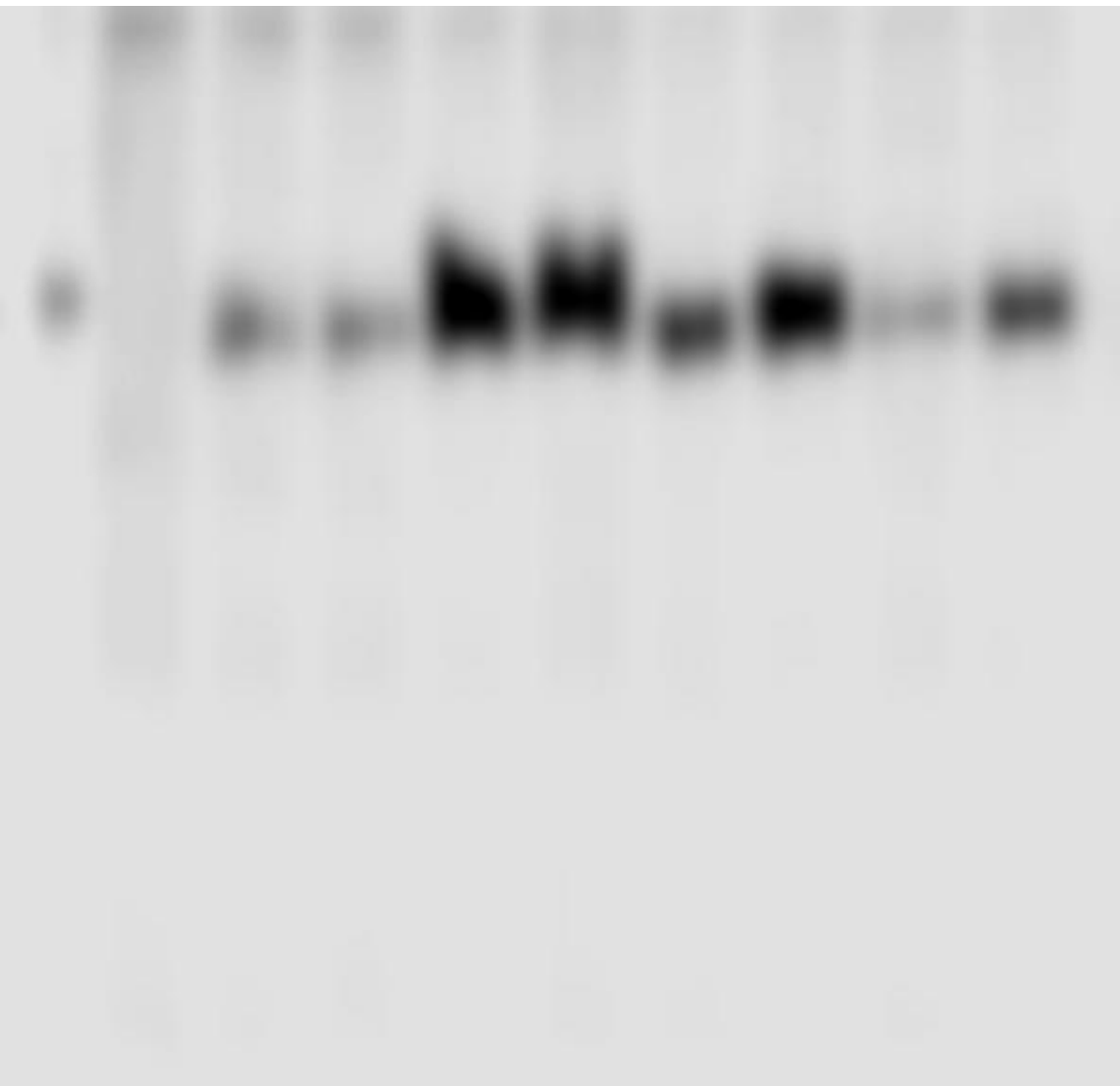

fig2c CEBP

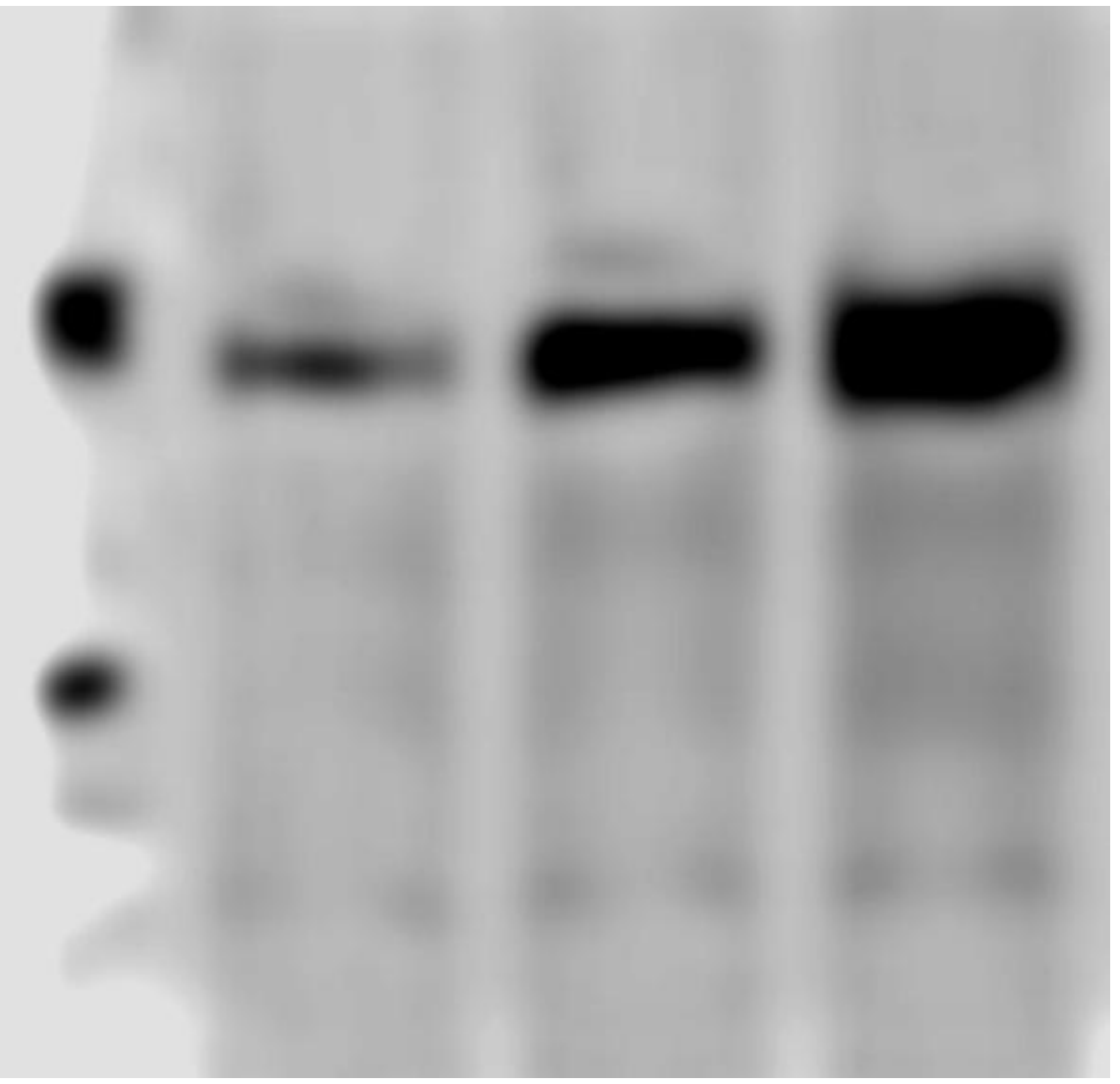

fig2c MAFbx .jpg

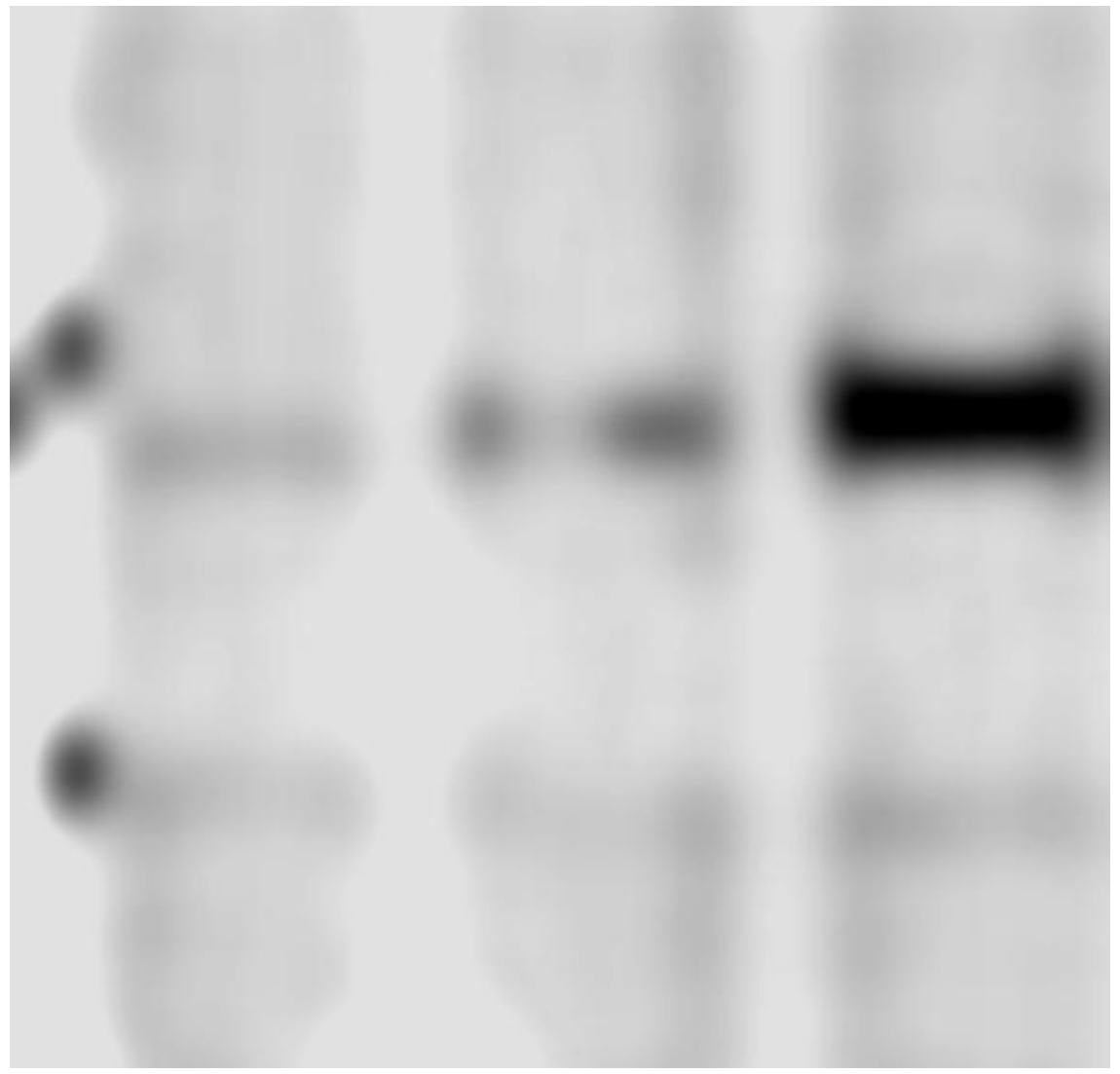

fig2c MuRF1

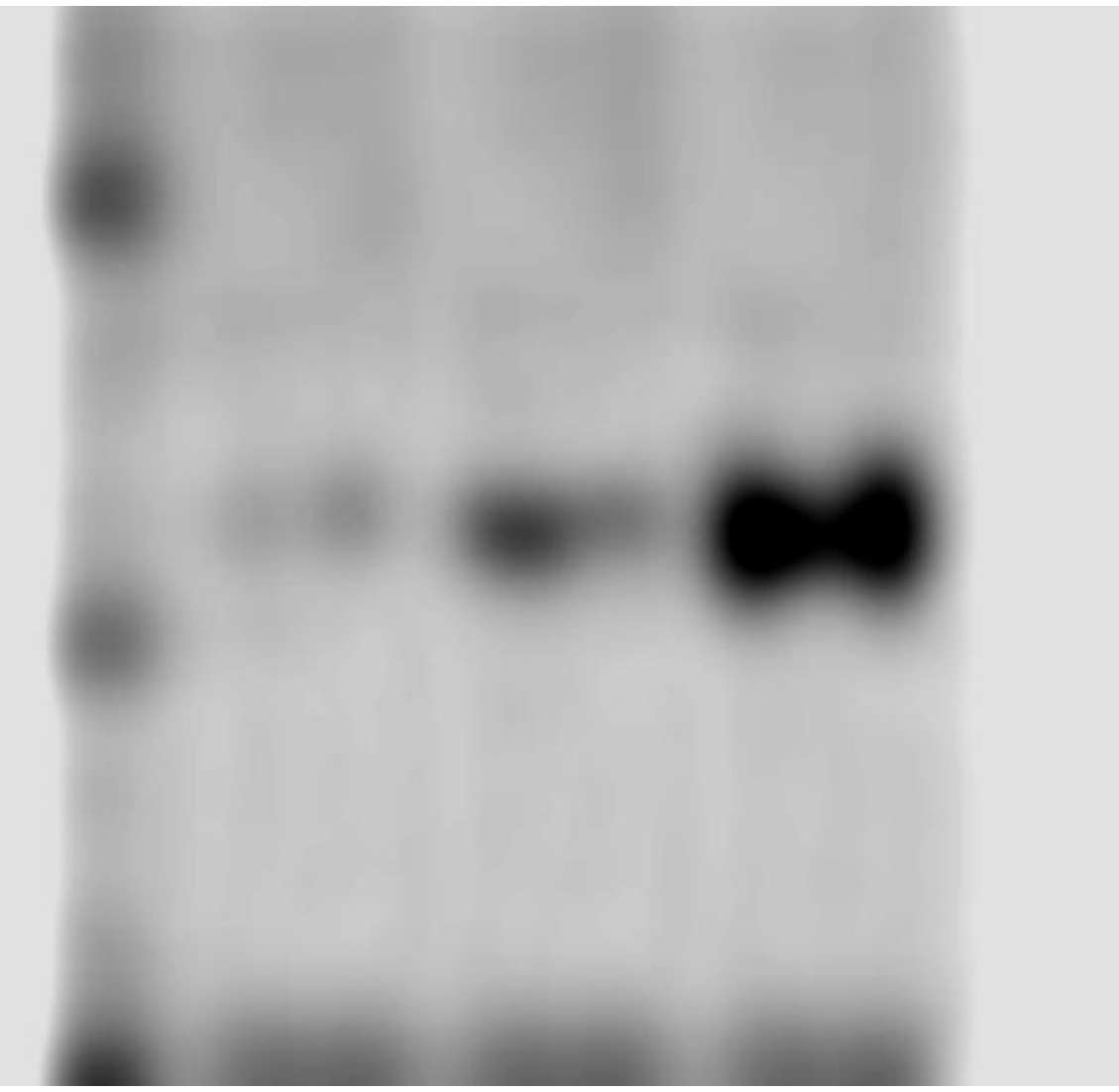

fig2c myostatin

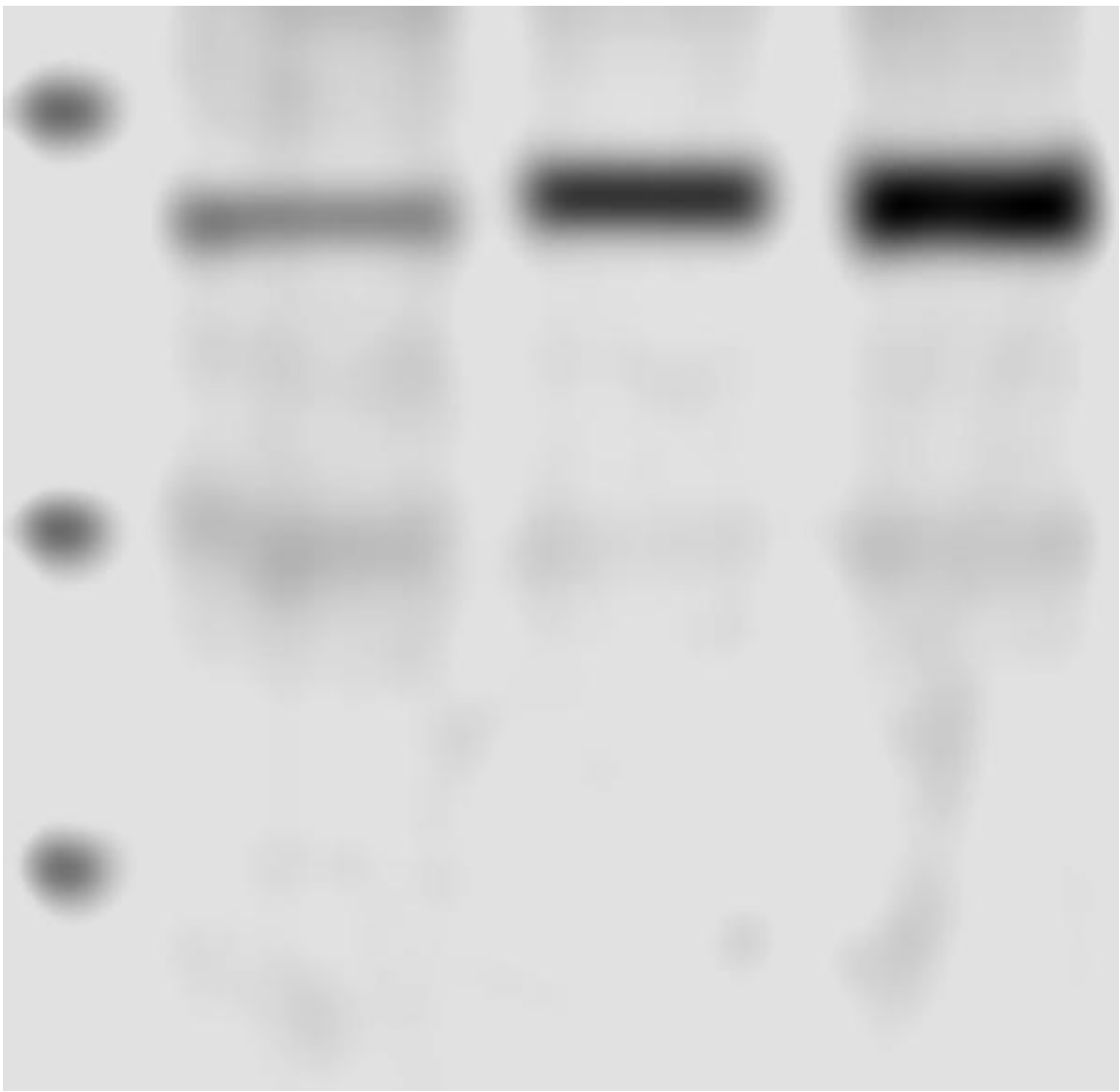

fig2c PKCλ

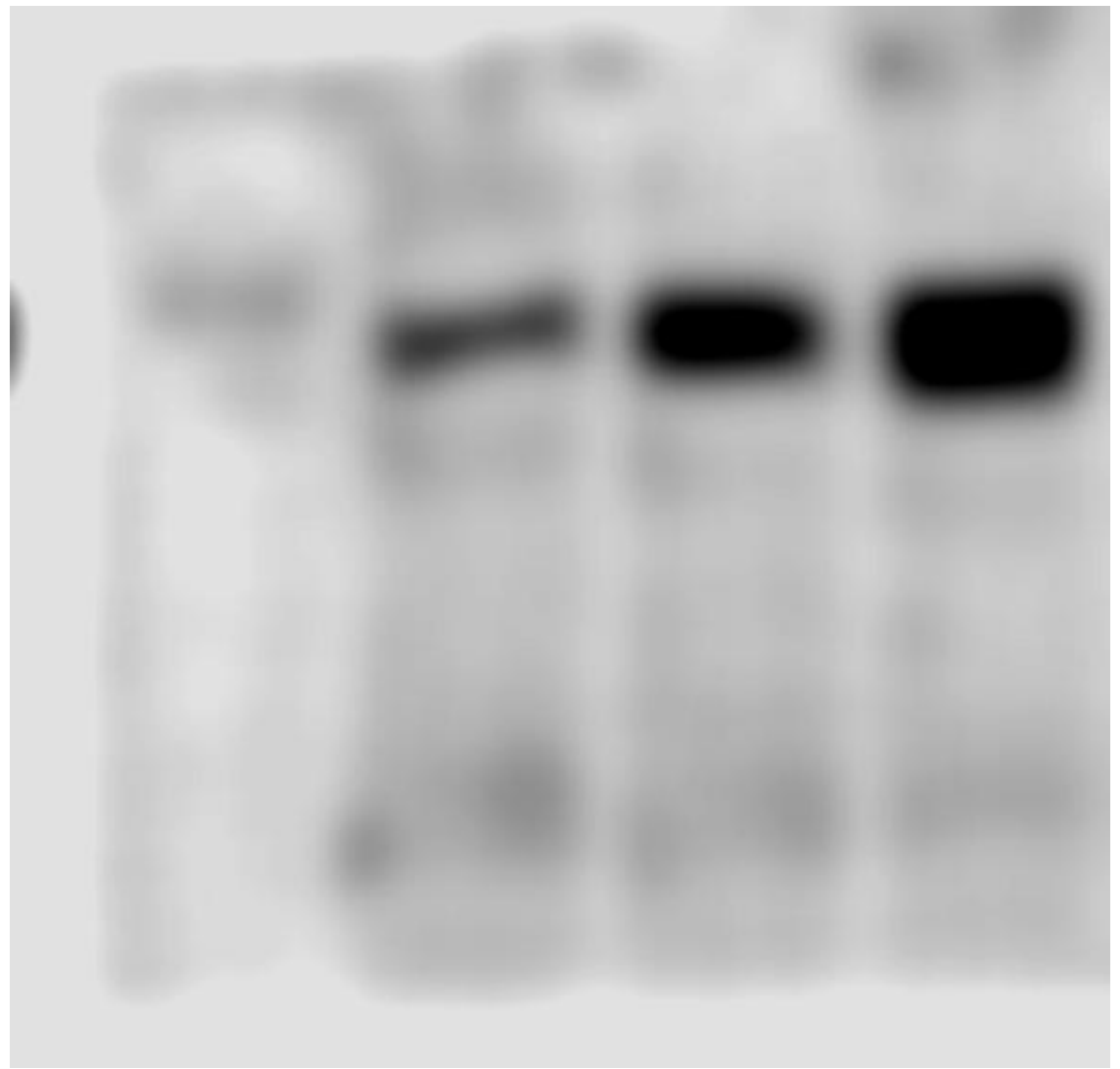

fig2c p-PKCλ

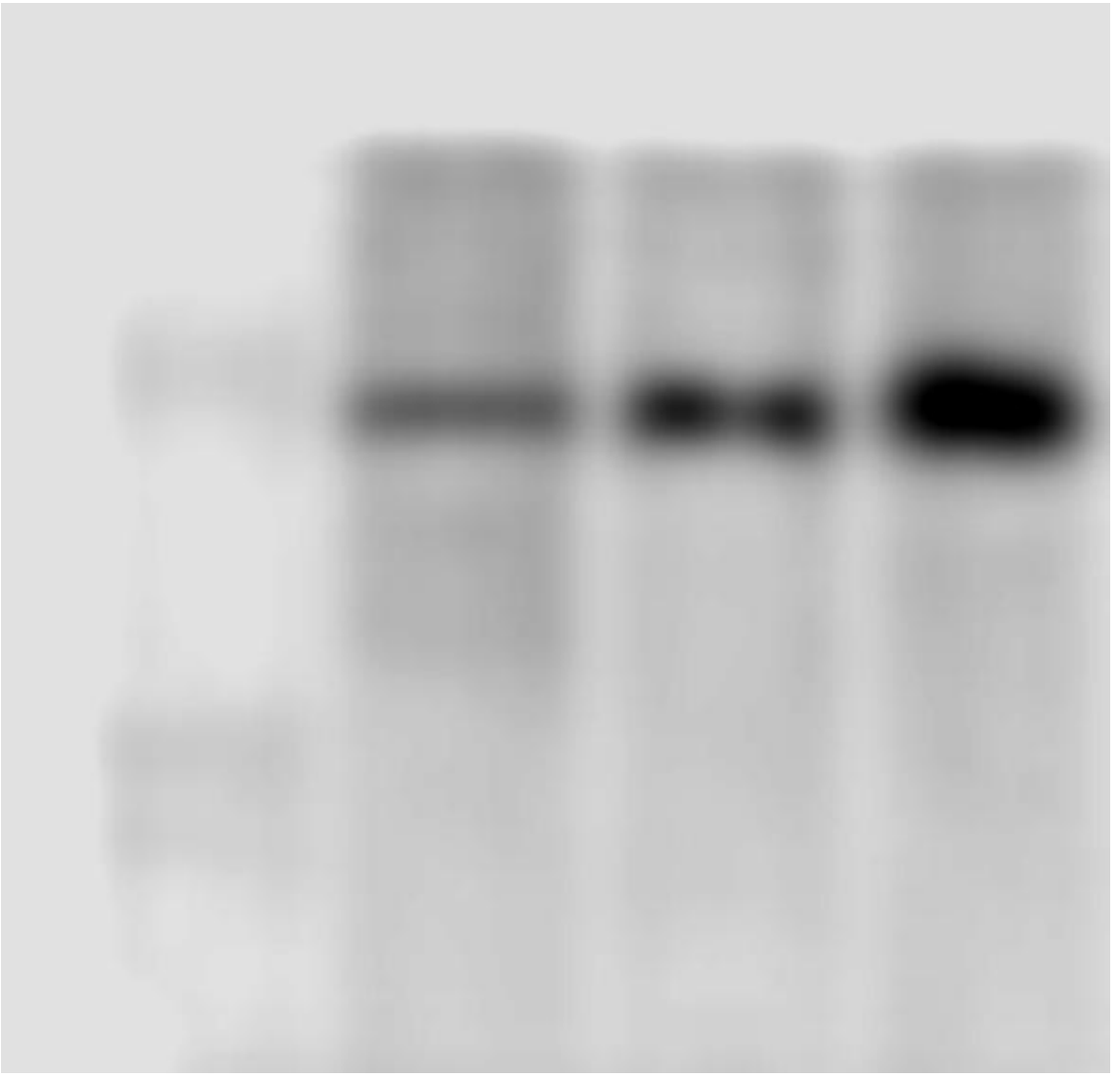

fig2c PSTAT3.jpg

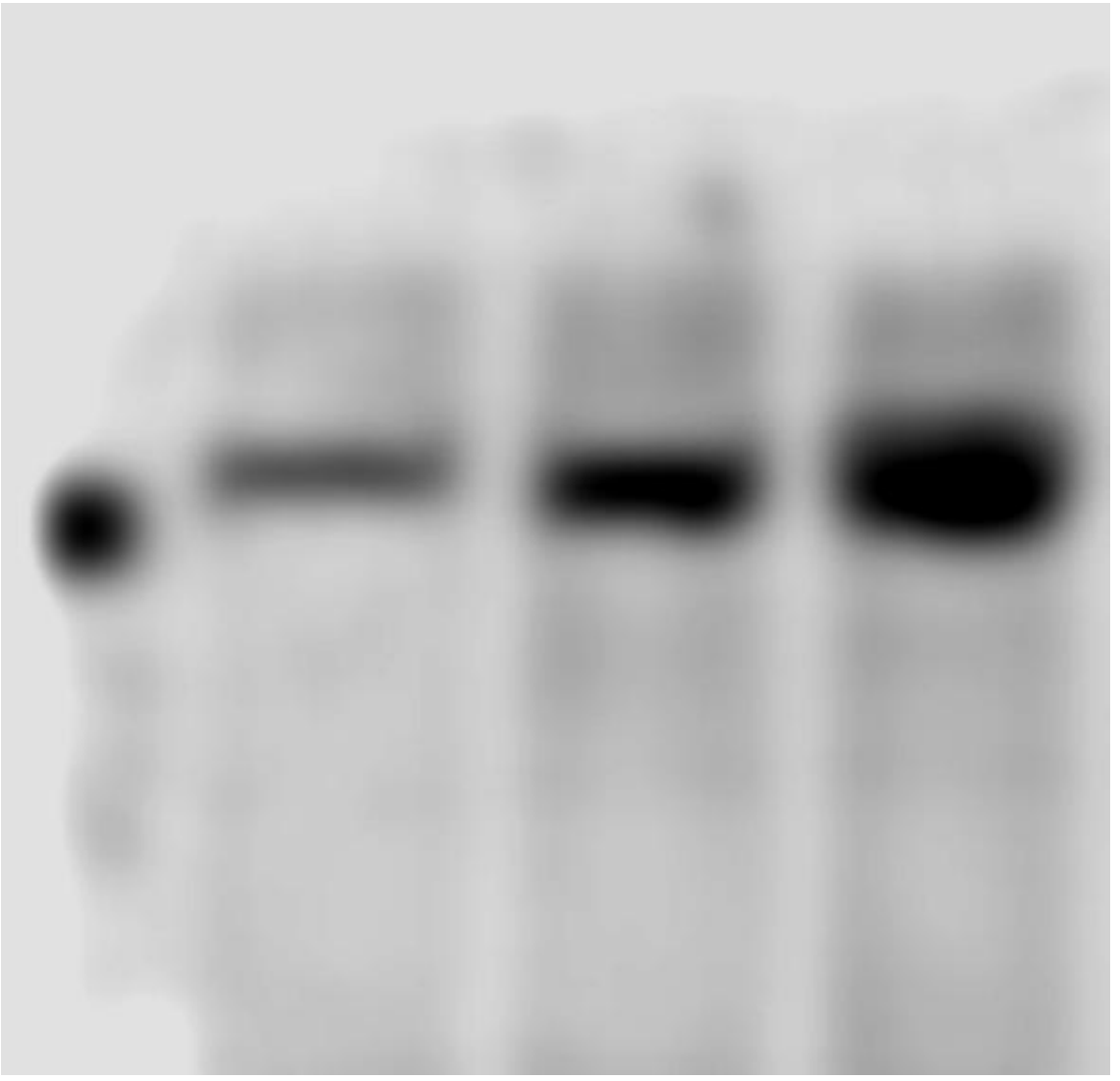

fig2c STAT3

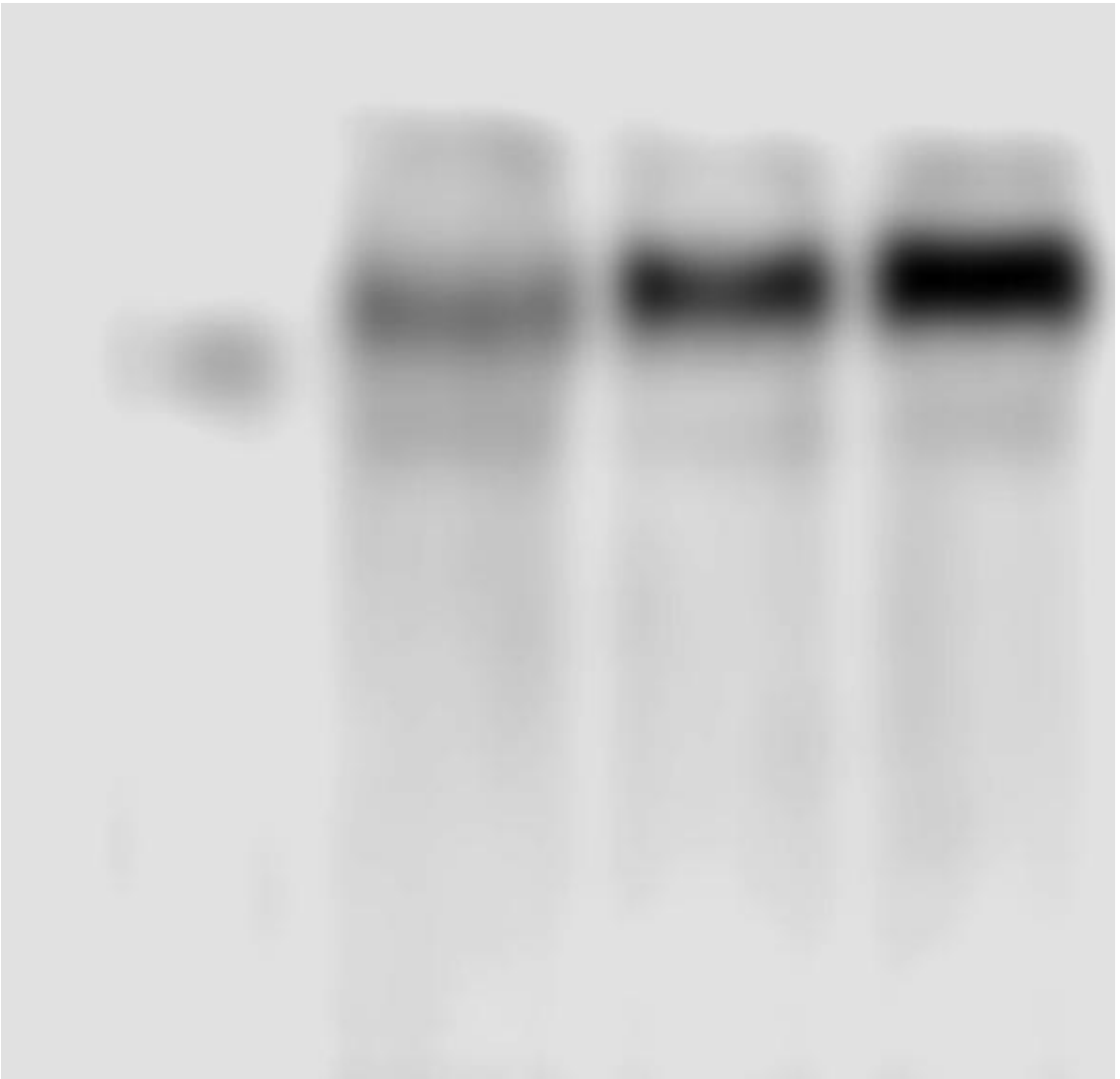

fig2c β-tubulin.jpg

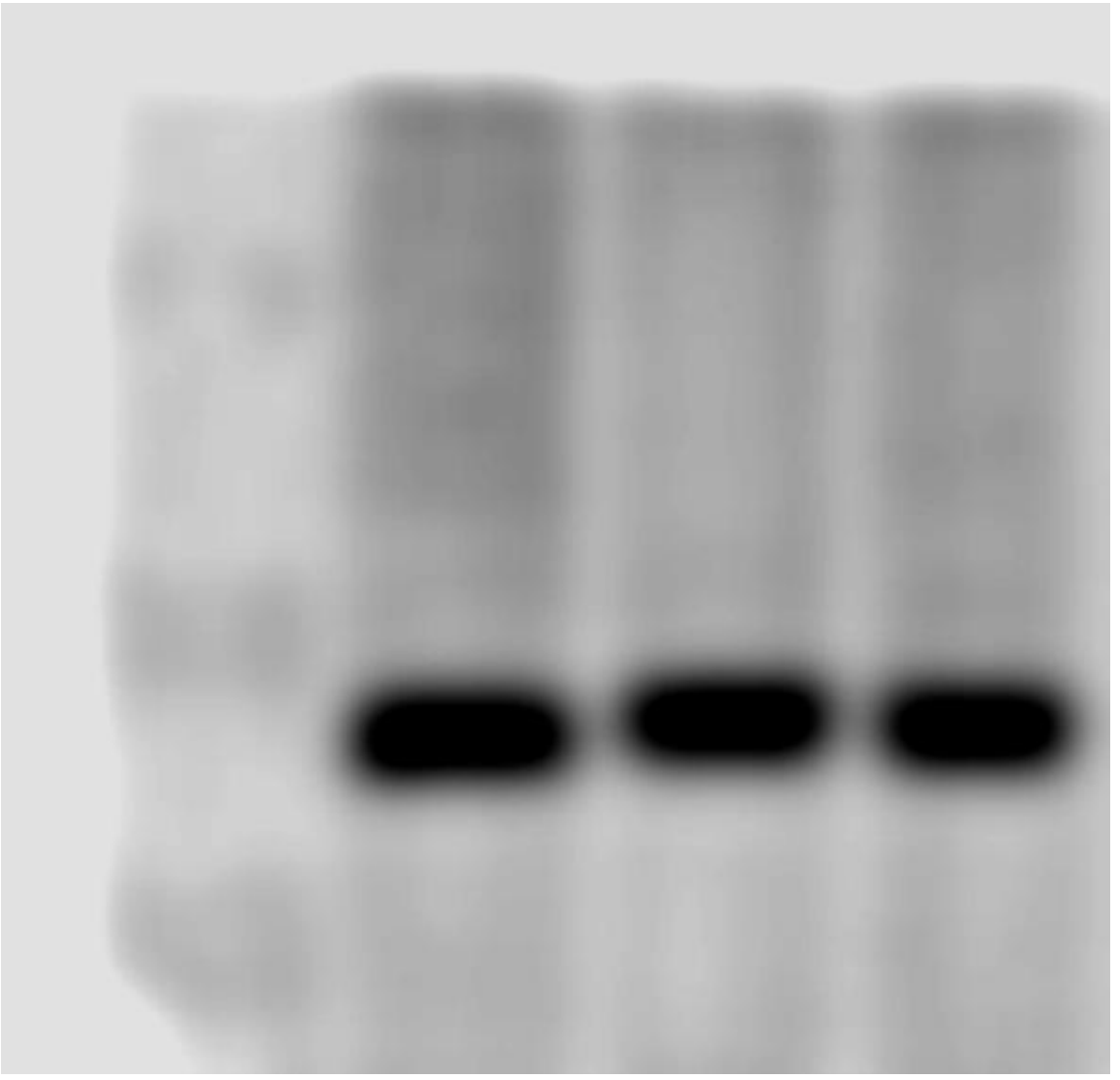

fig2f CEBP

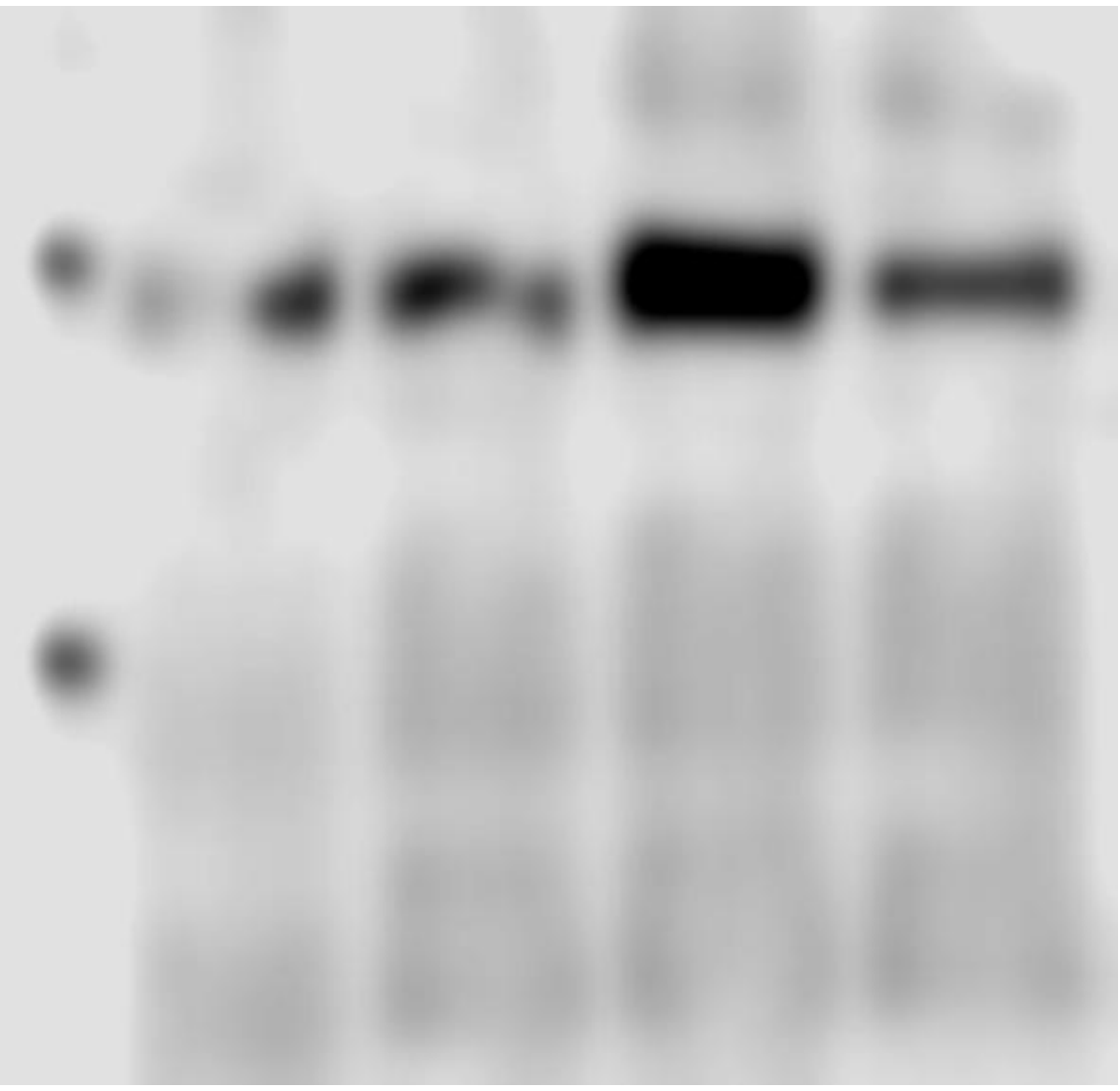

fig2f MAFbx.jpg

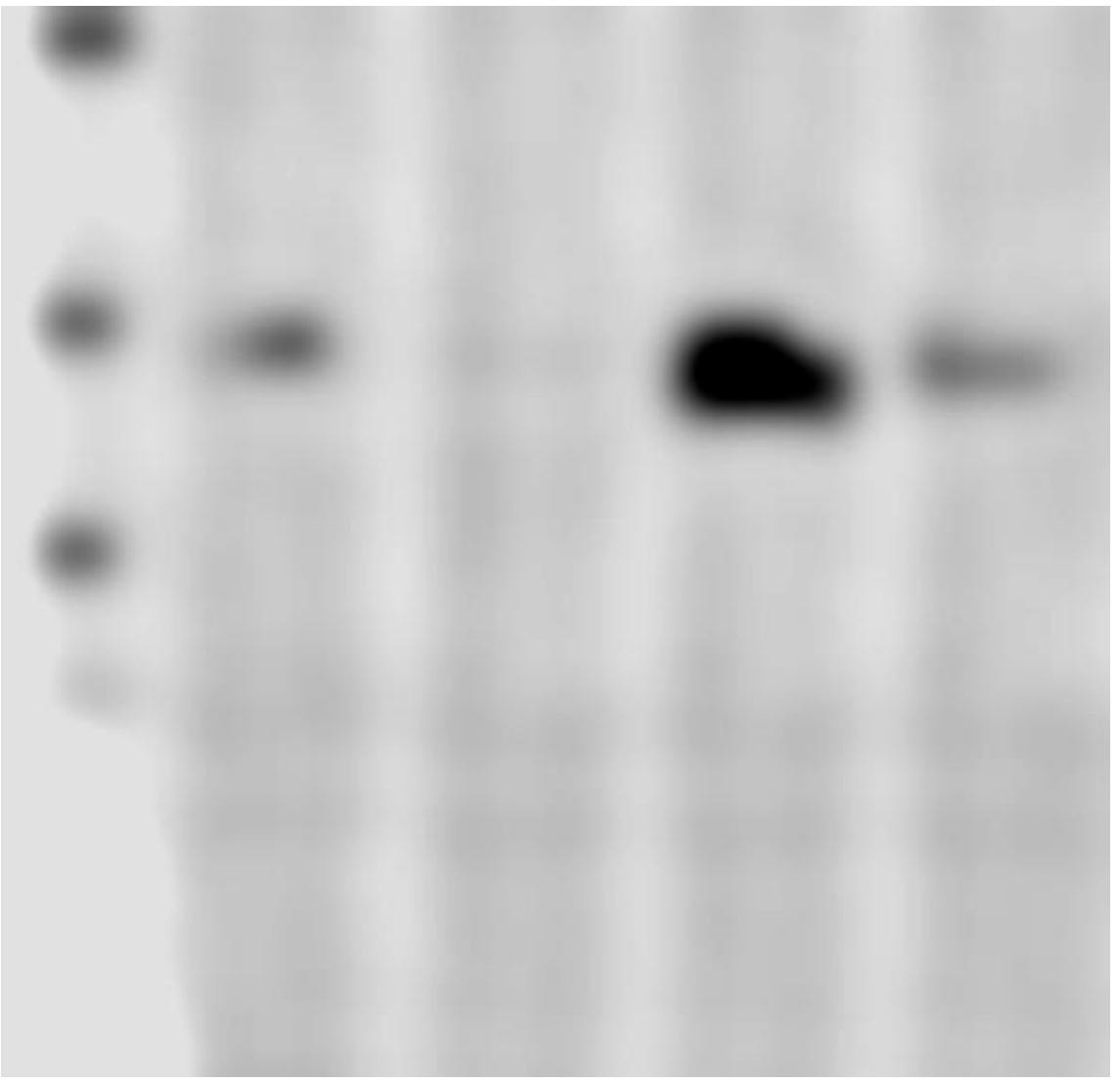

fig2f MuRF1.jpg

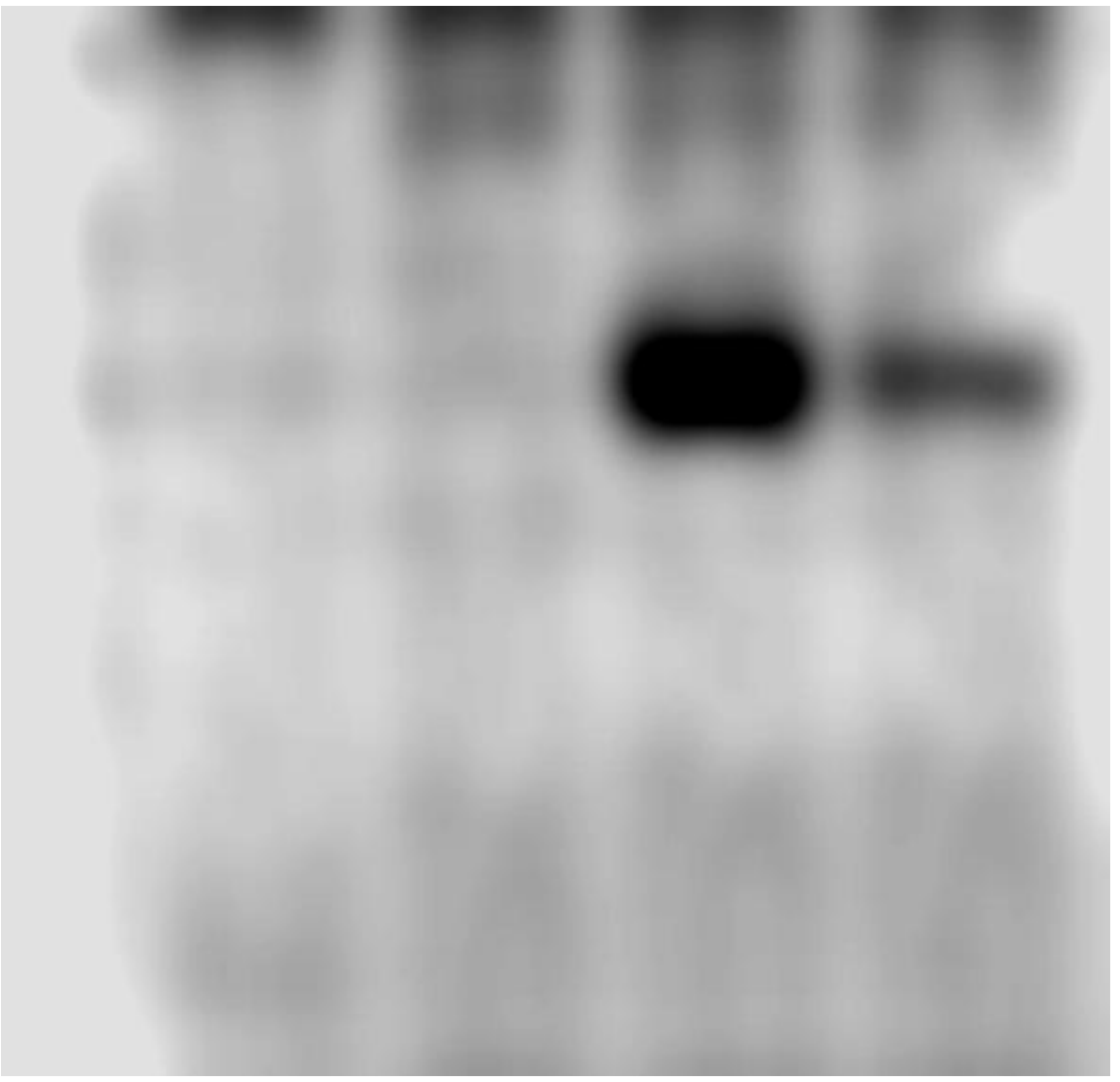

fig2f myostatin

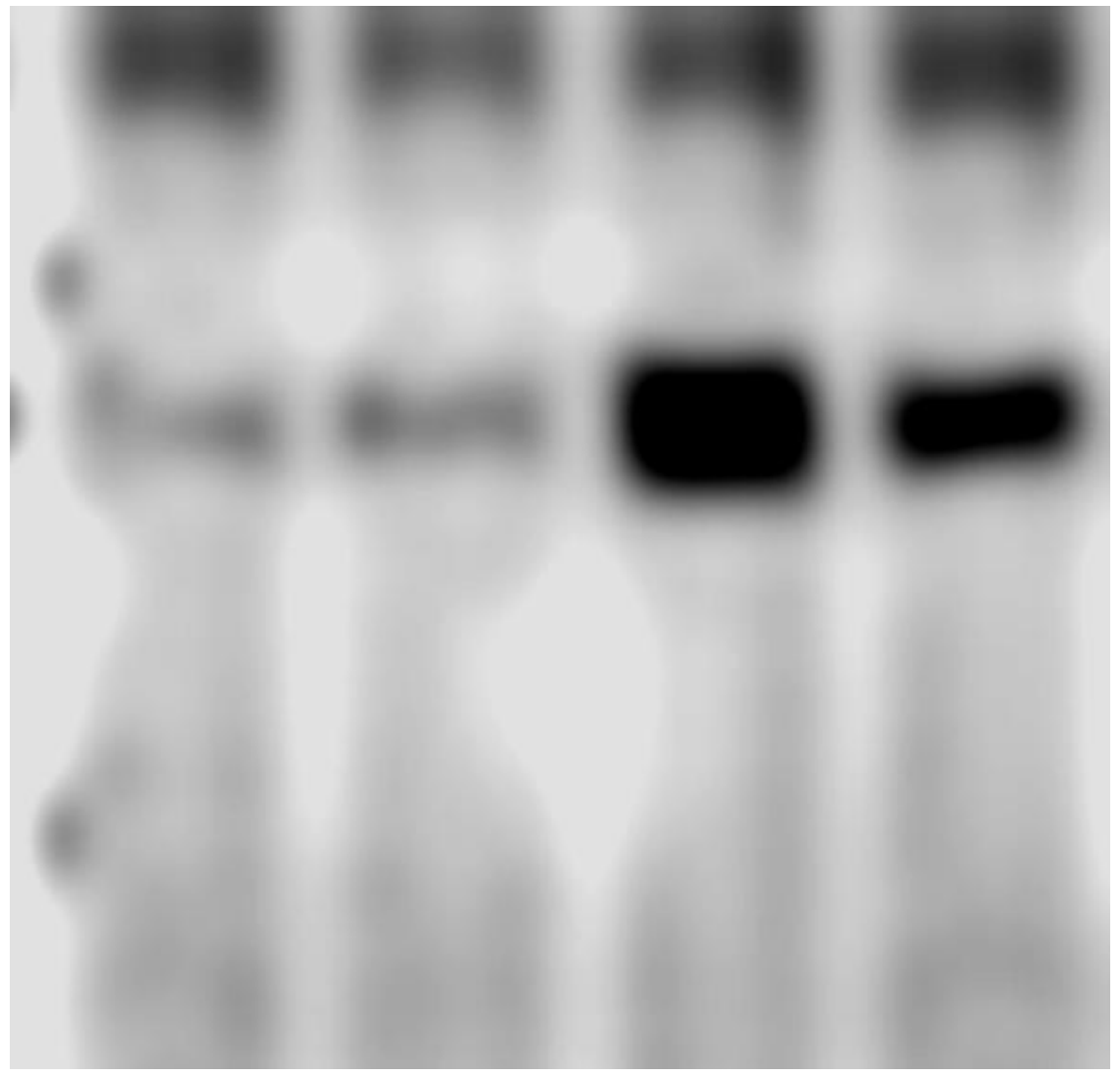

fig2f PKCλ

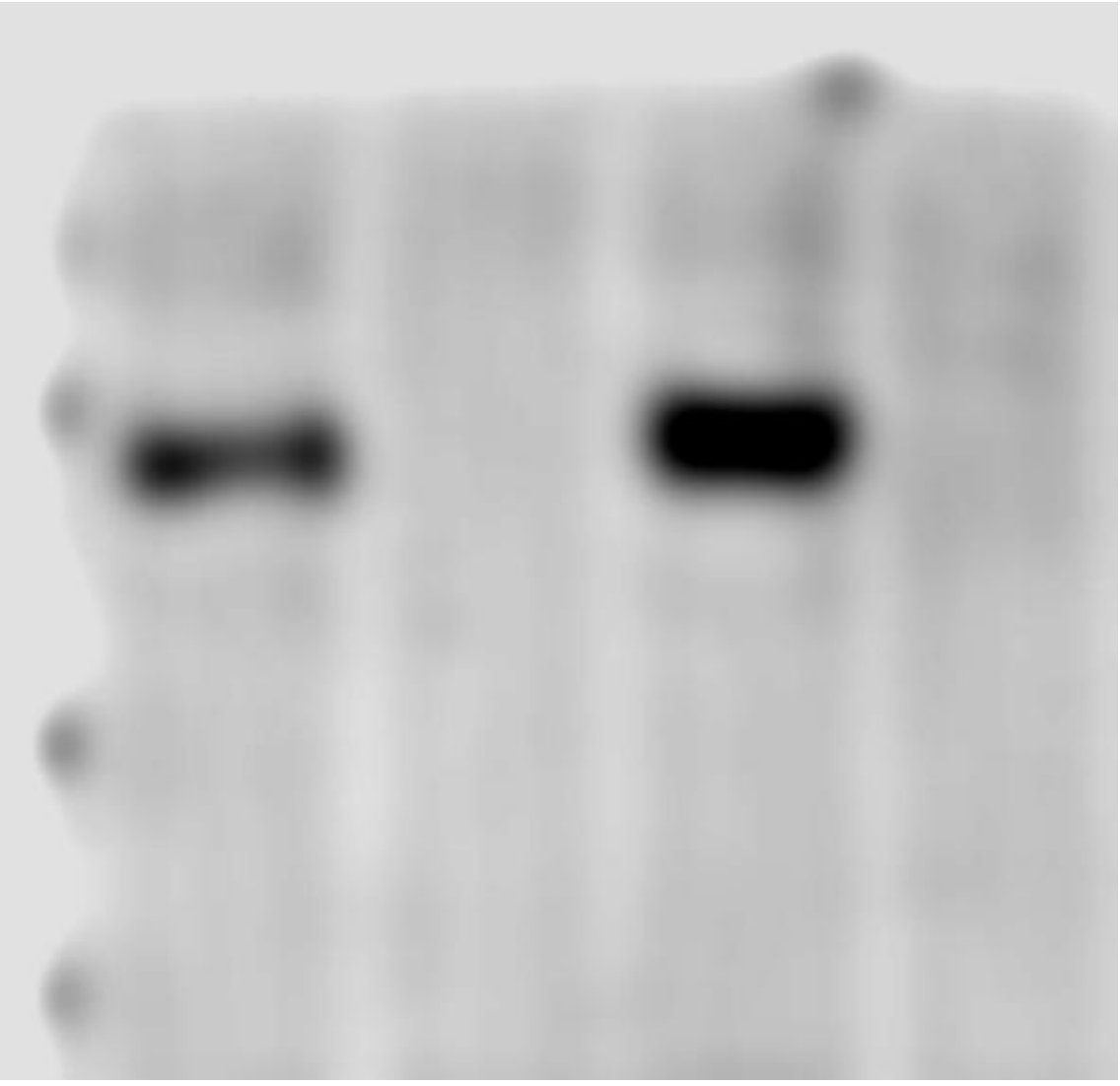

fig2f p-PKCλ

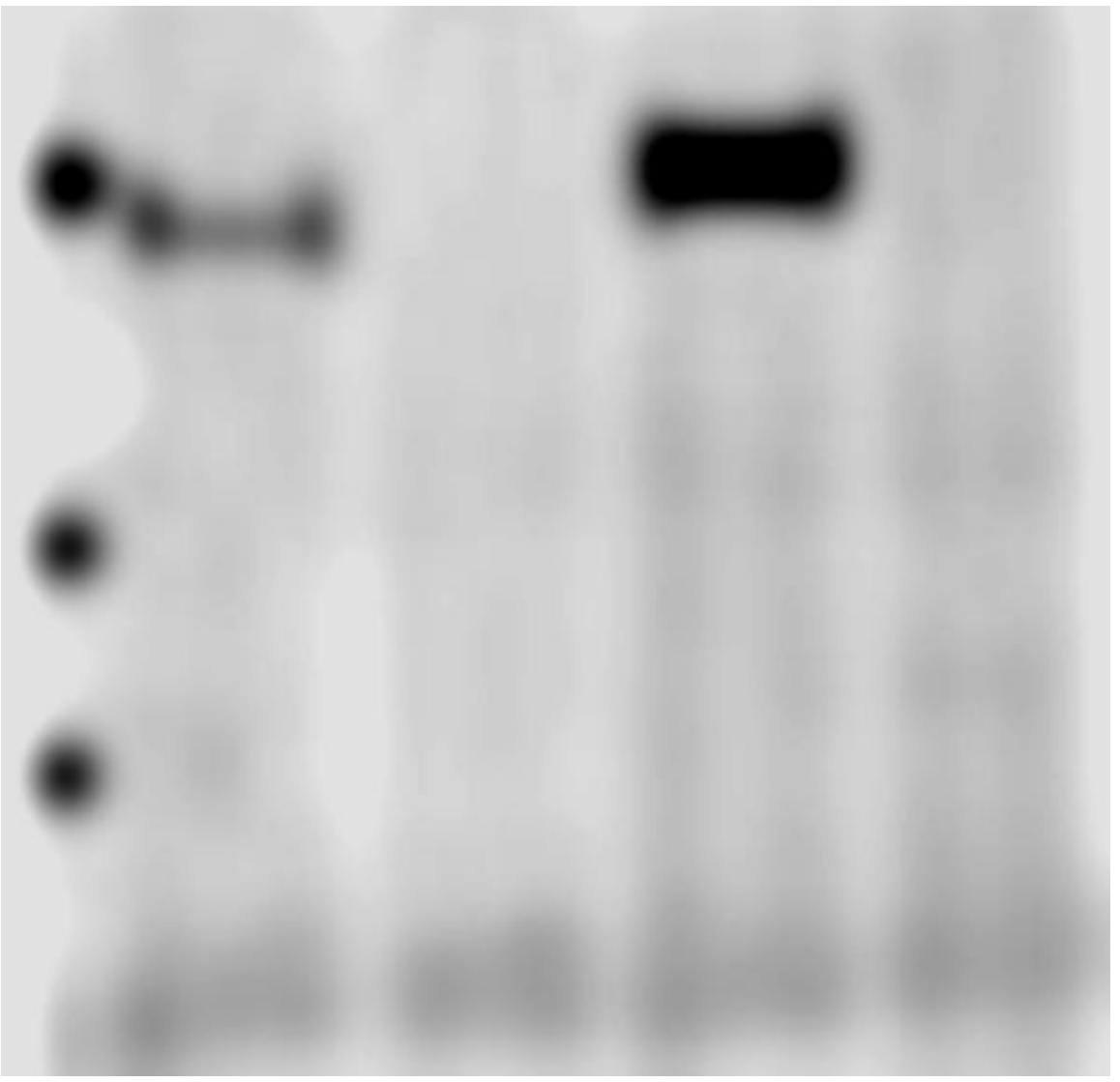

fig2f PSTAT3

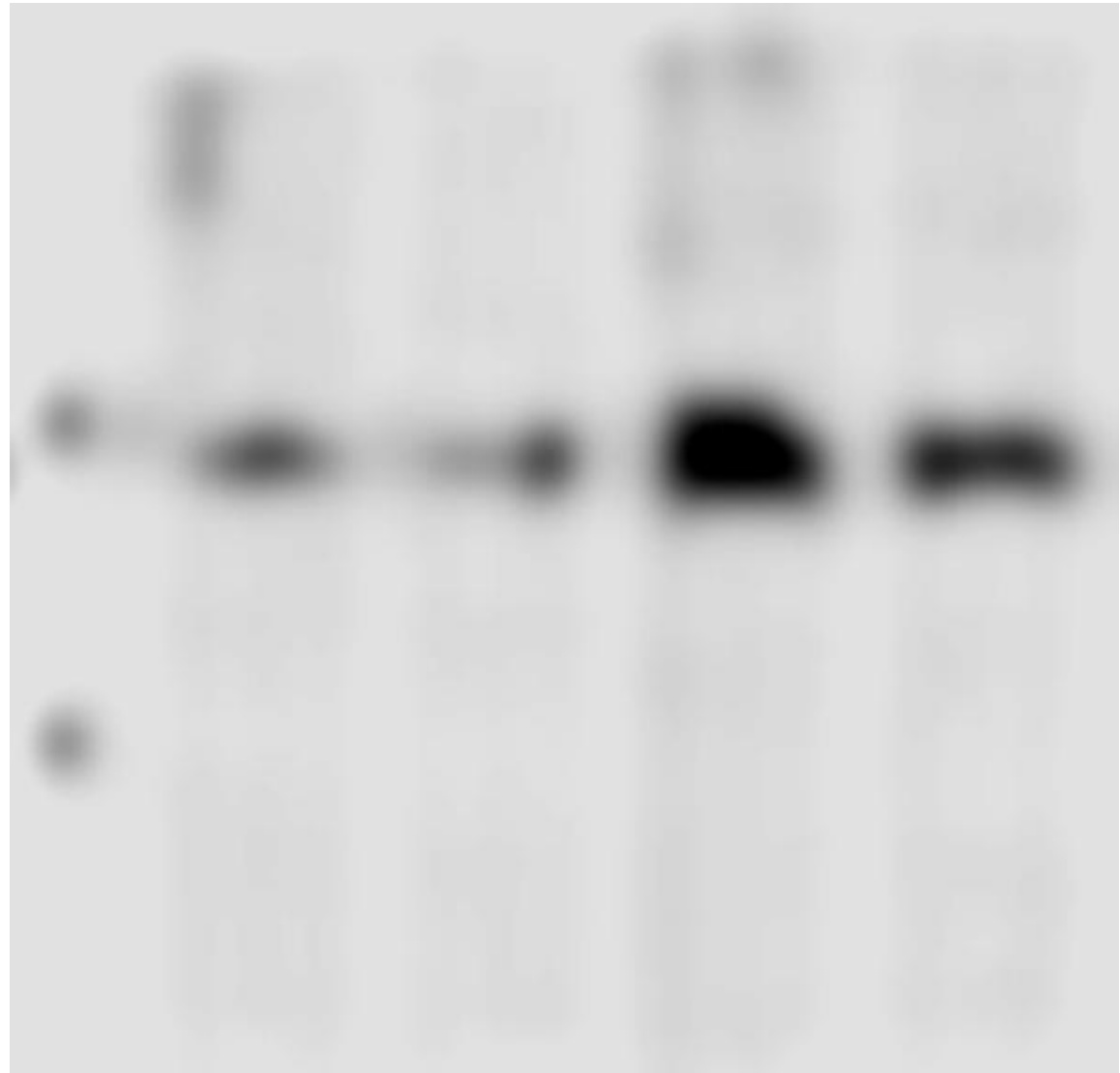

fig2f stat3

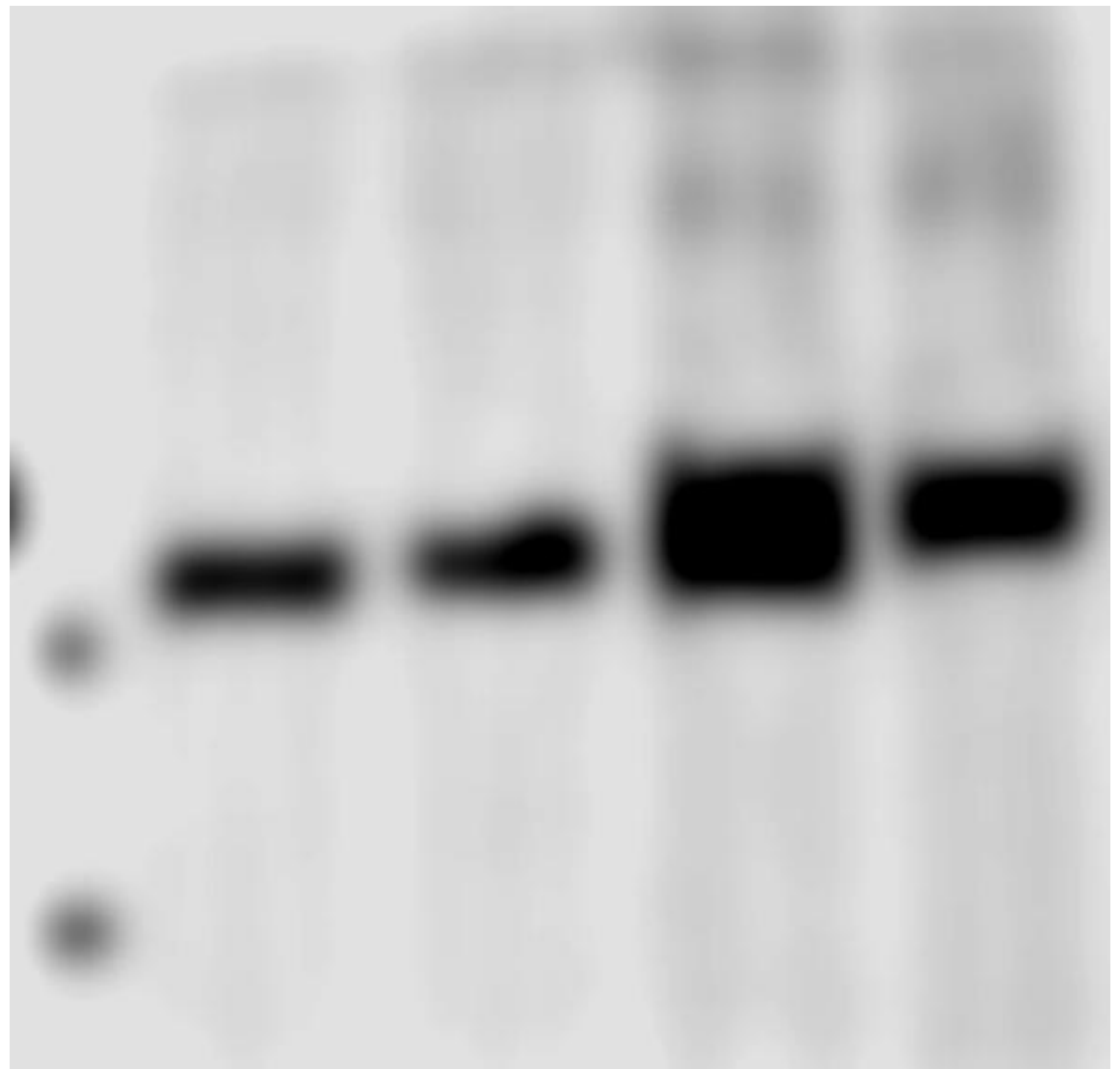

fig2f β-tubulin.jpg

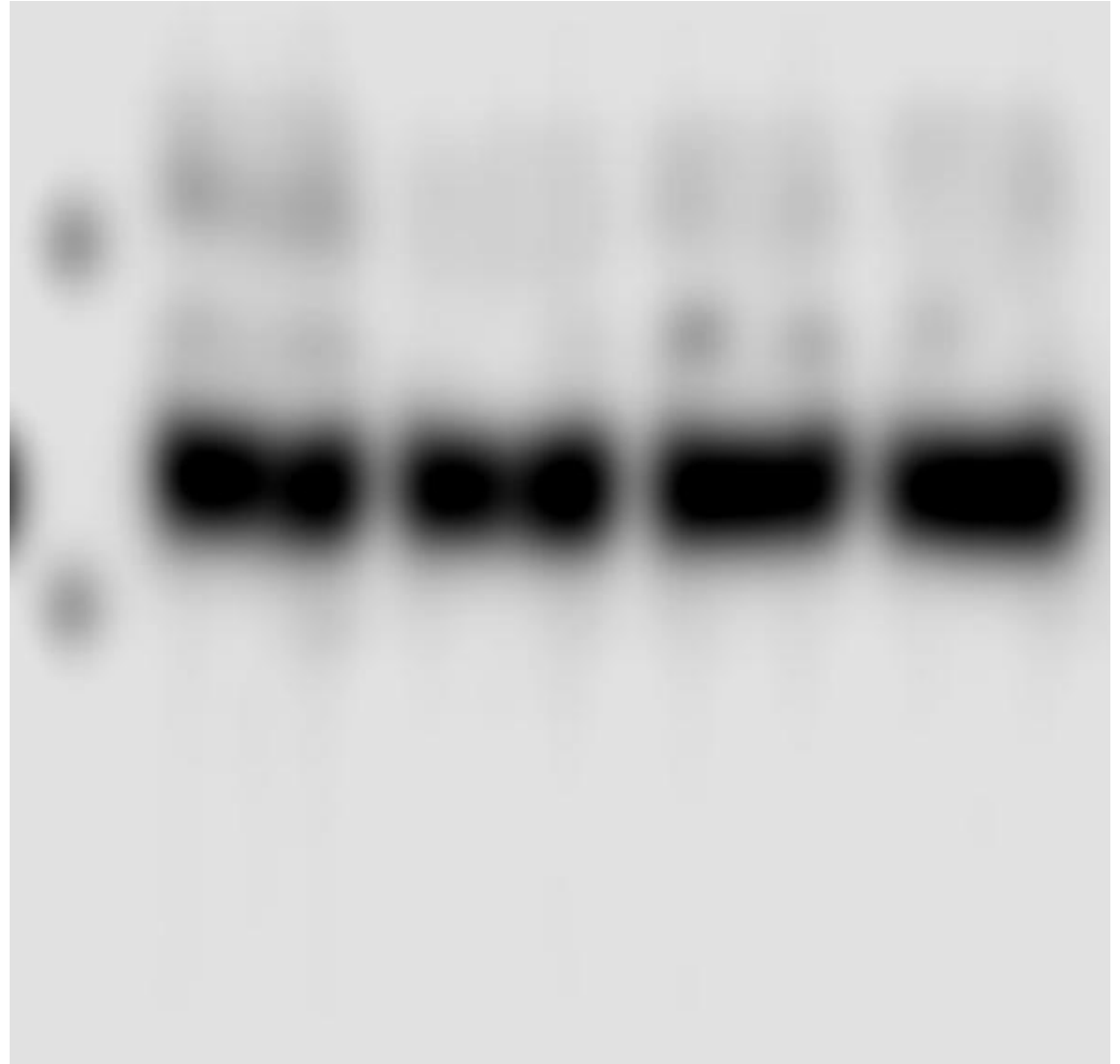

fig3B B-Tublin .jpg

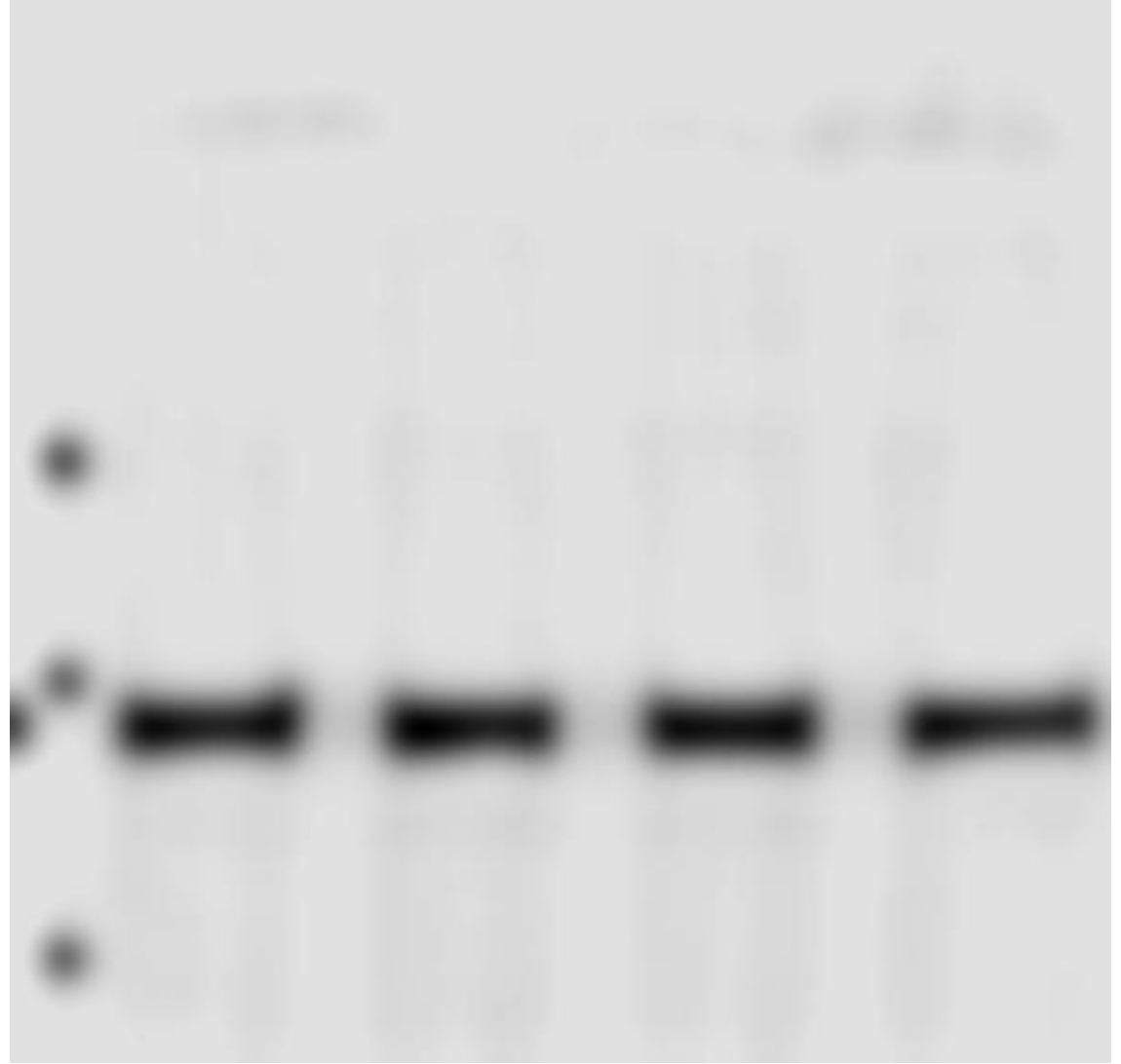

fig3B C-Ebp .jpg

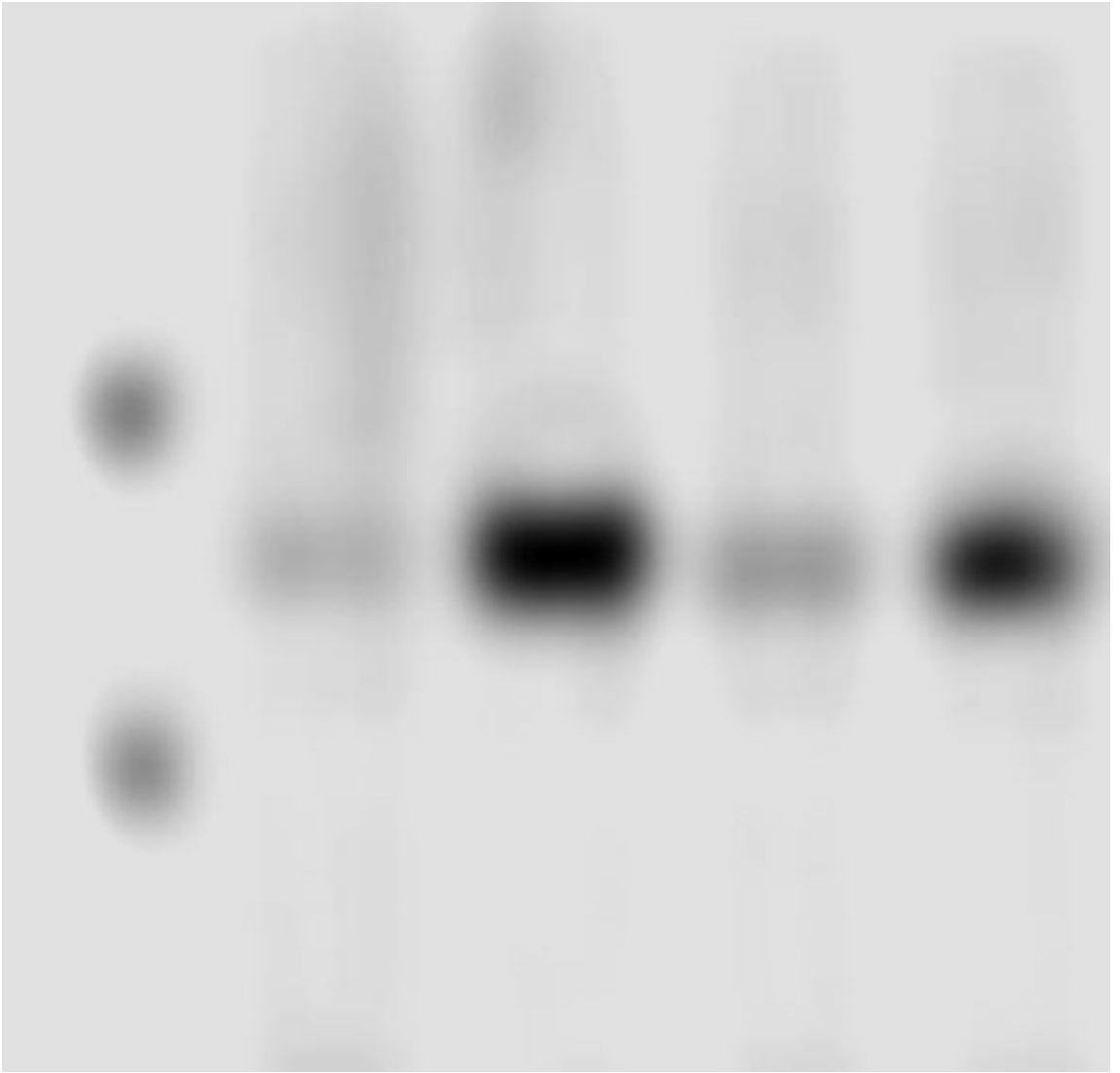

fig3B MAFbx .jpg

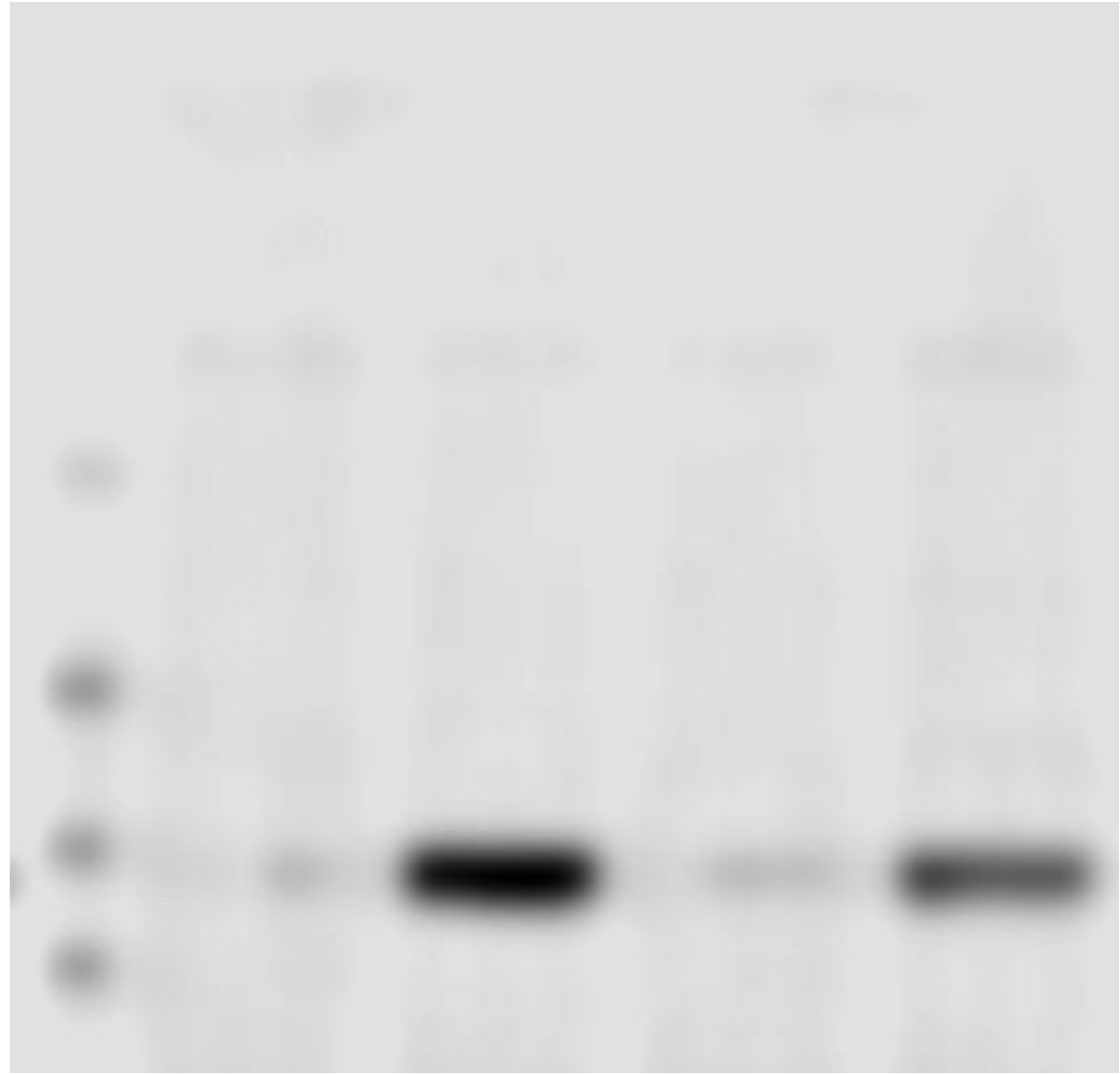

fig3B MuRF1.jpg

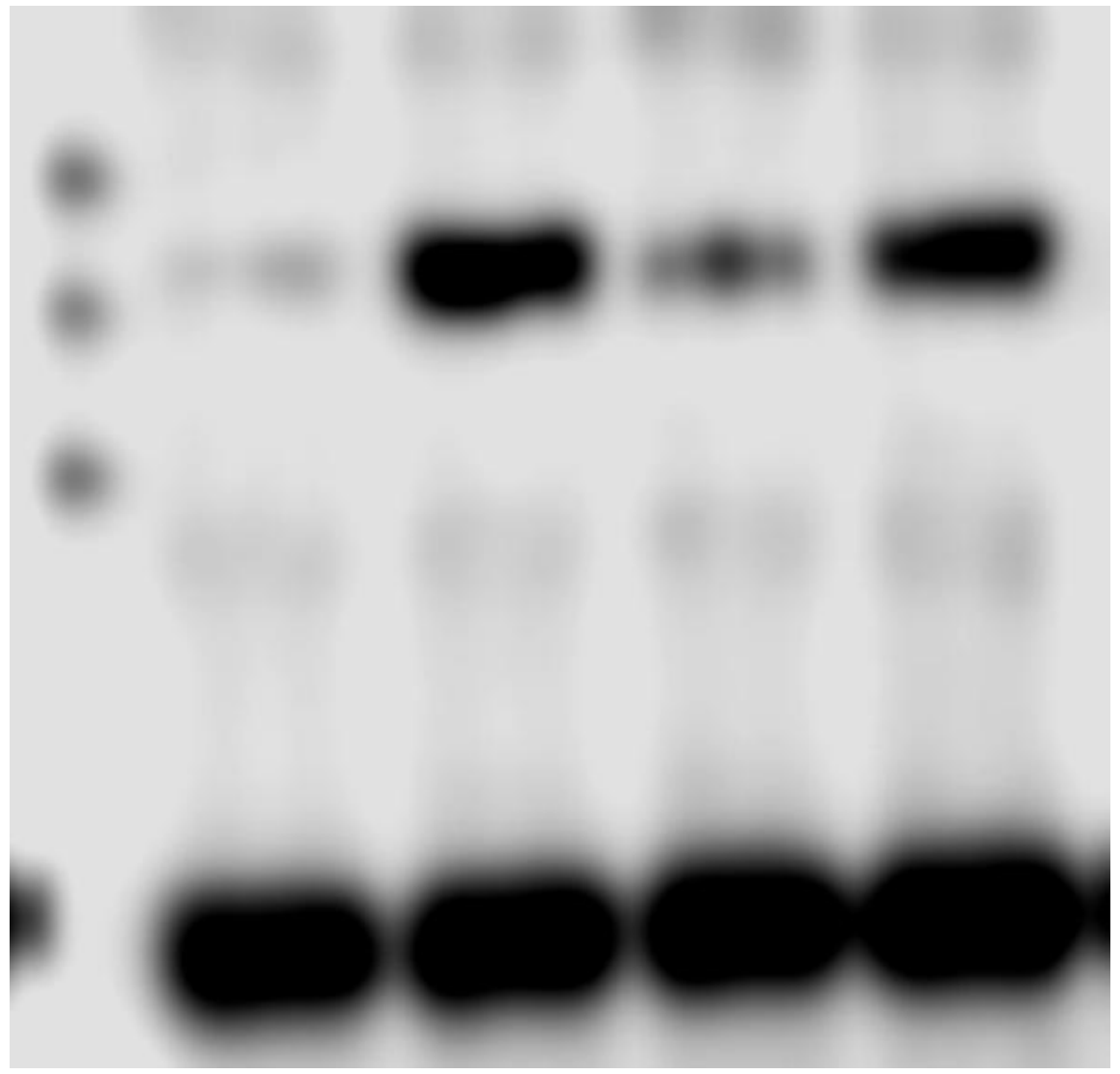

fig3B myostatin .jpg

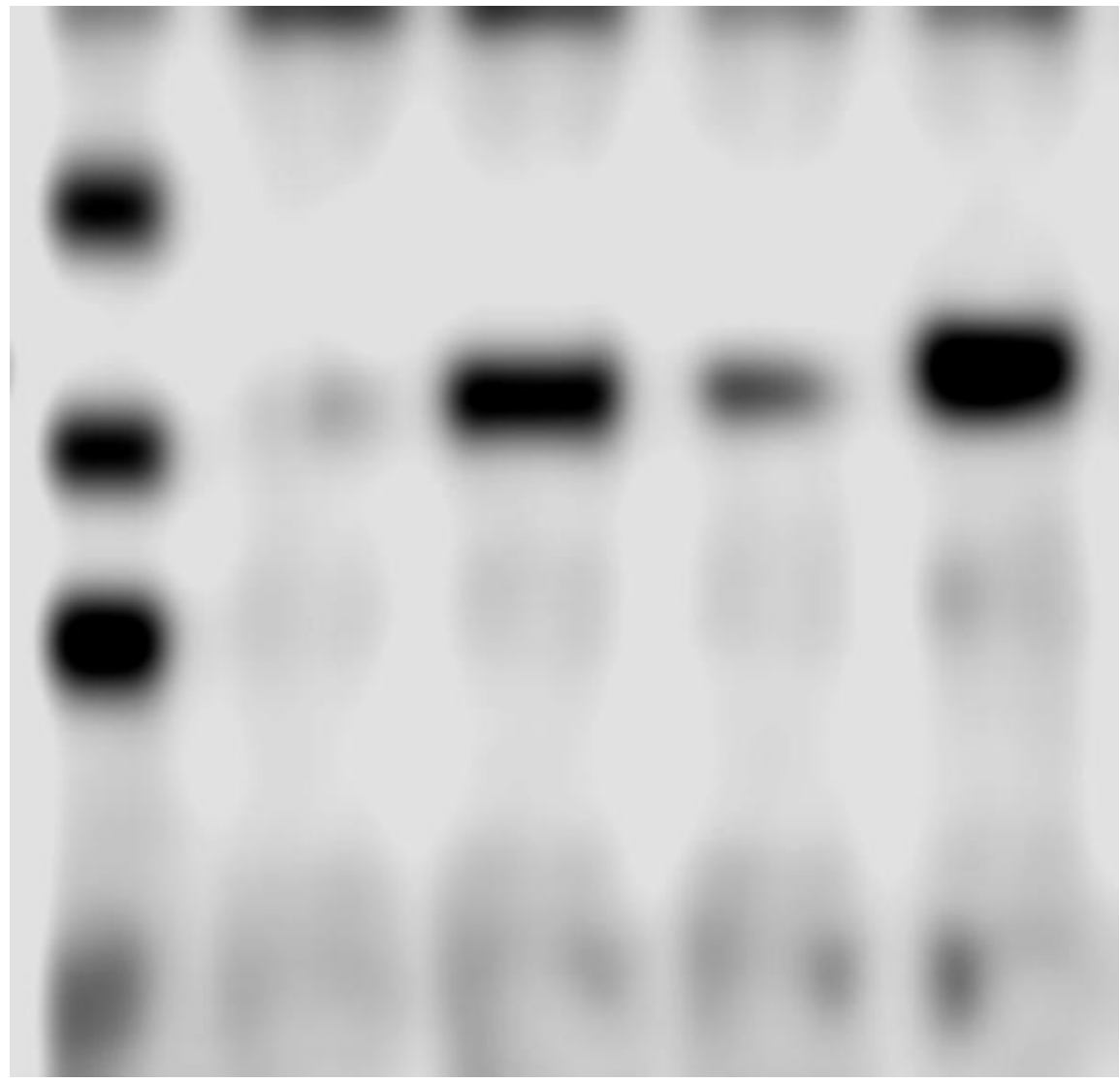

fig3B pkcλ.jpg

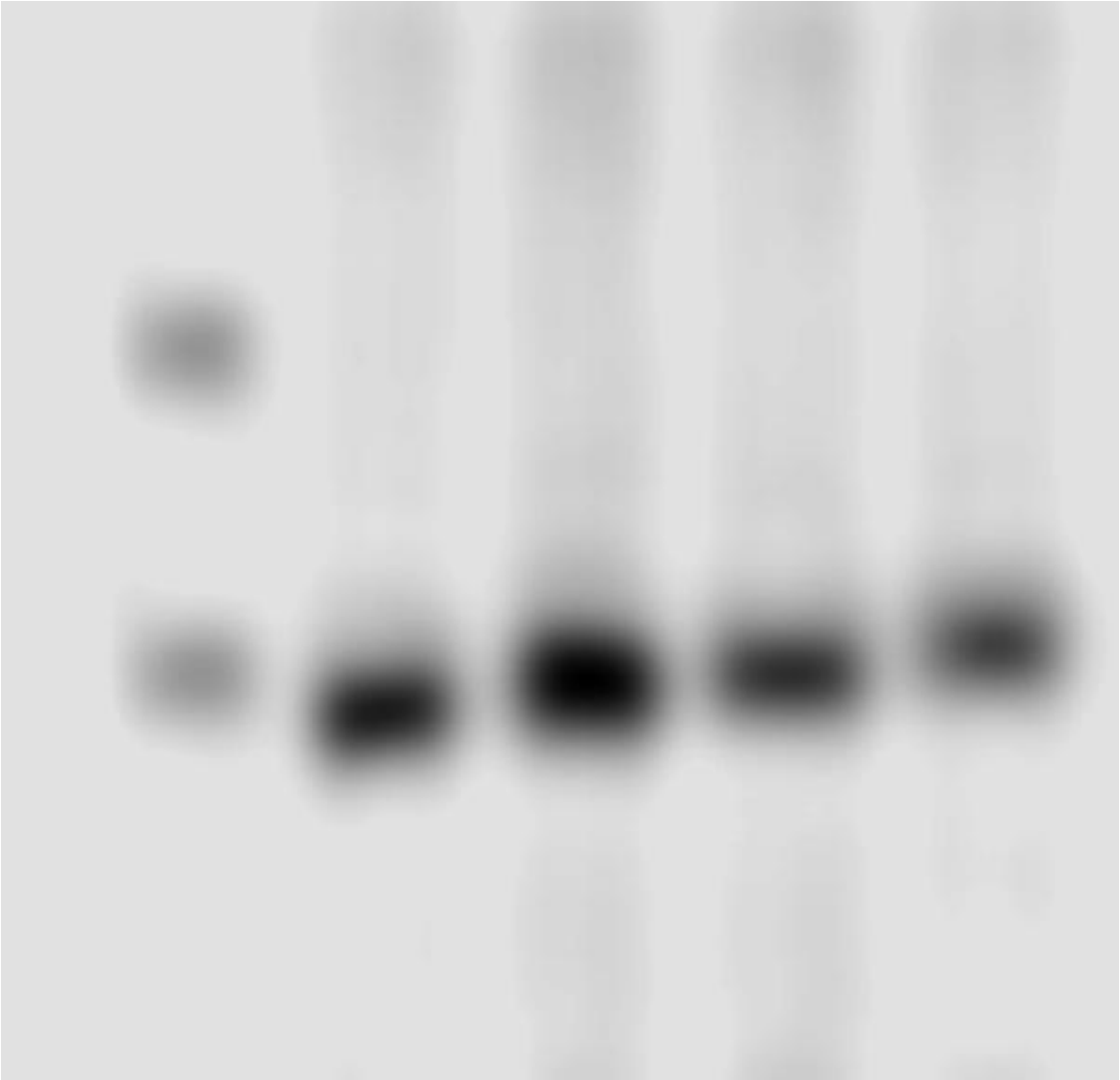

fig3B P-pkcλ .jpg

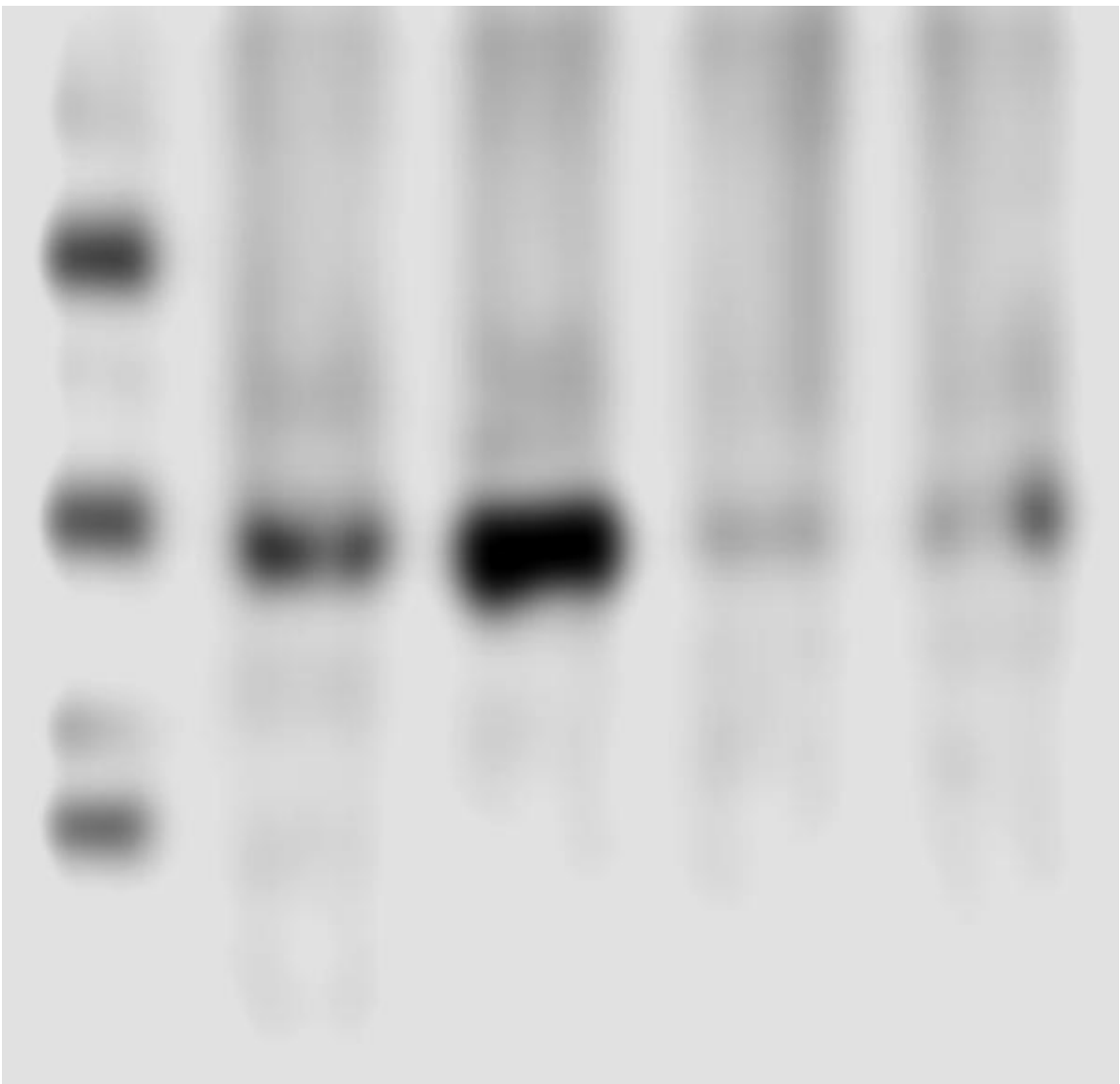

fig3B PSTAT3 .jpg

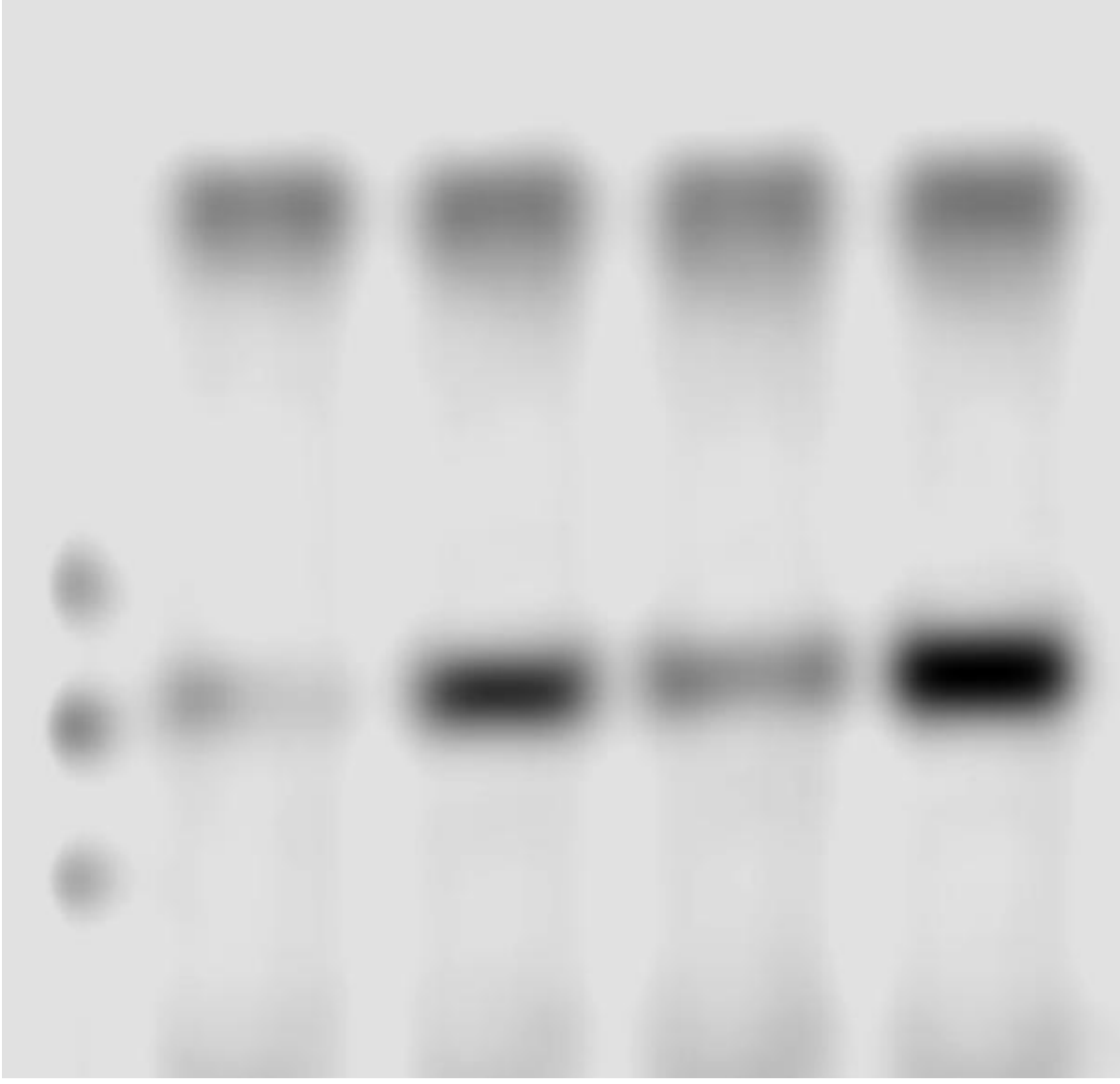

fig3B STAT3 .jpg

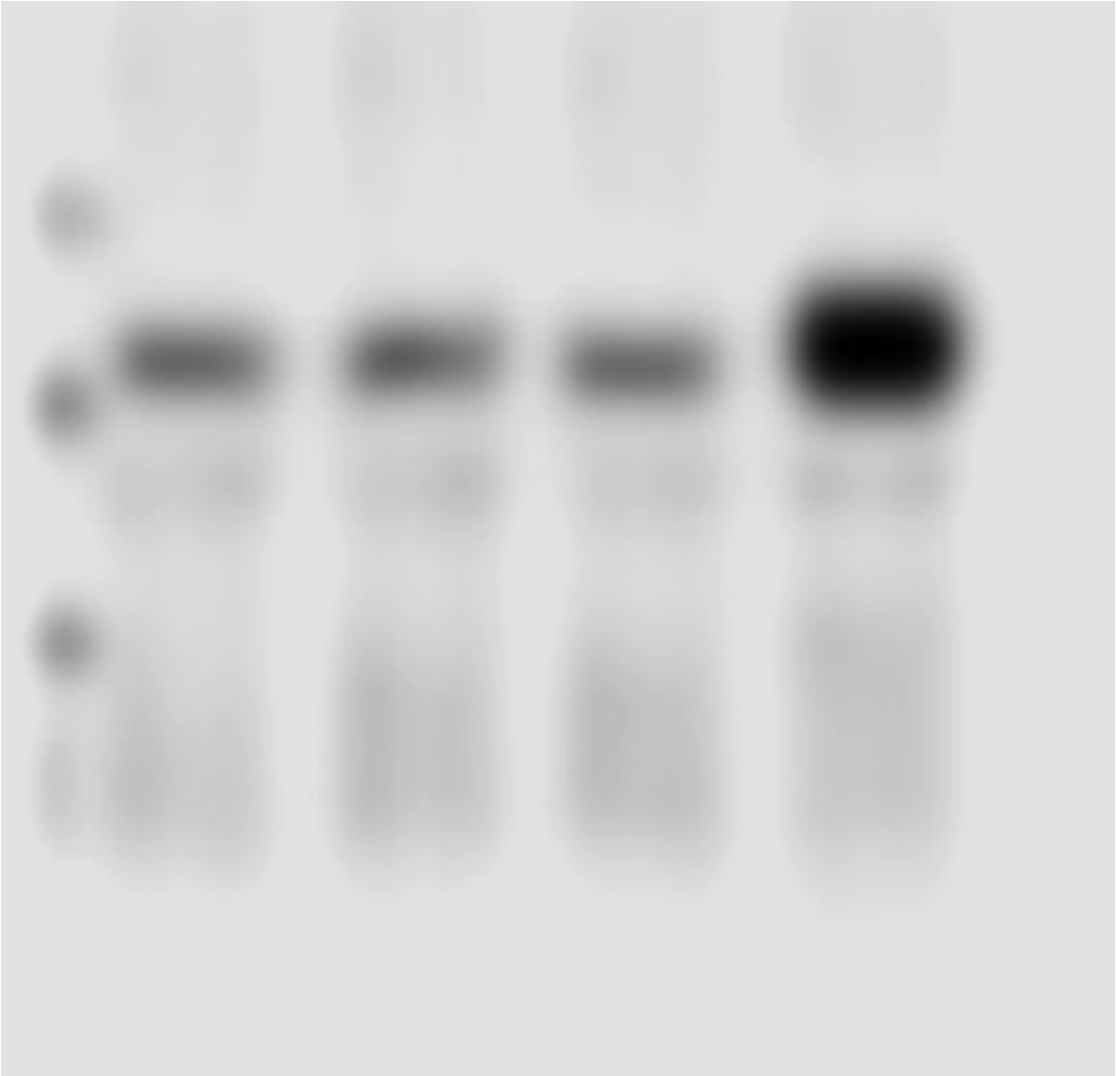

fig4c B-tubulin.jpg

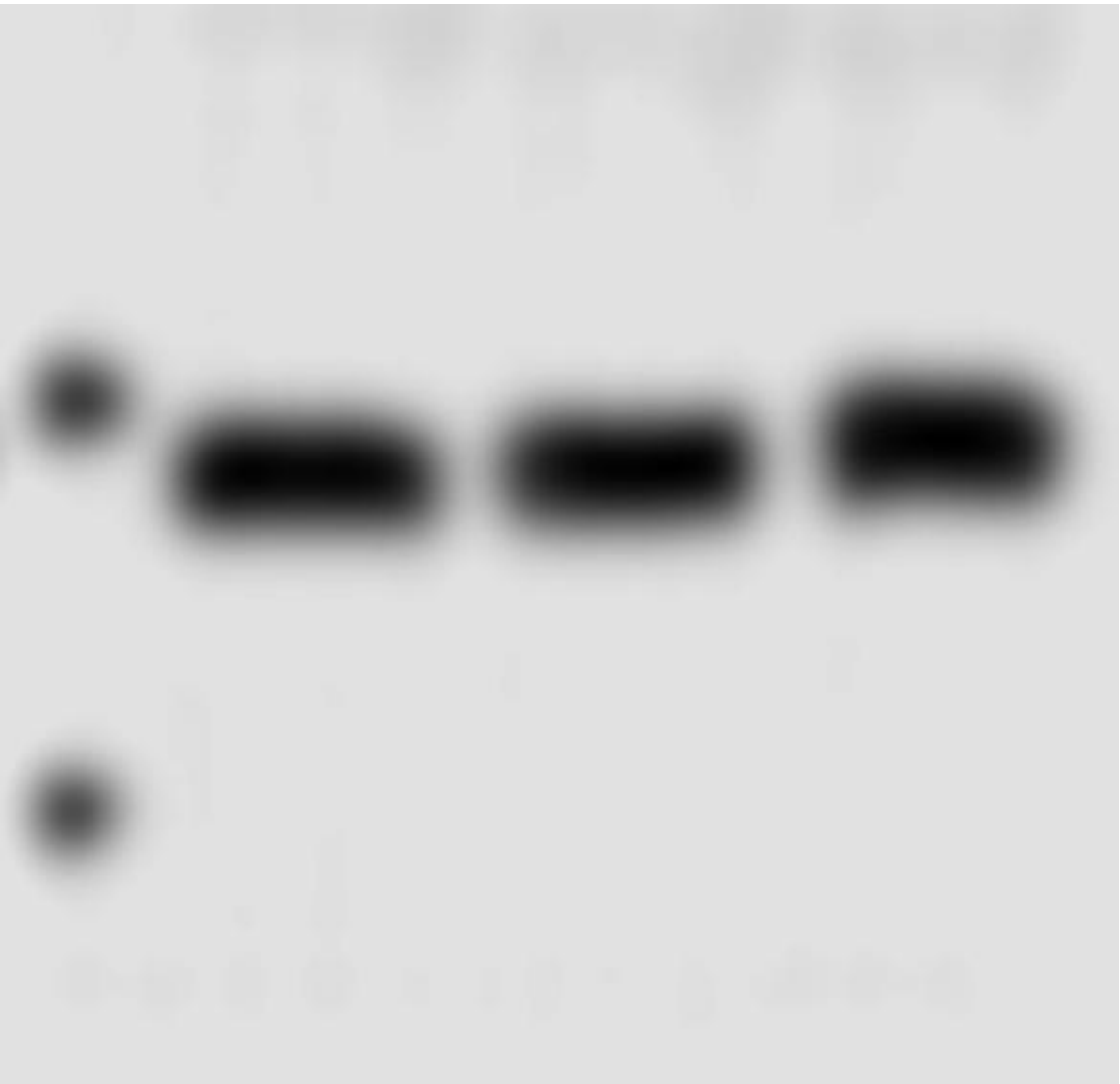

fig4c CEBP

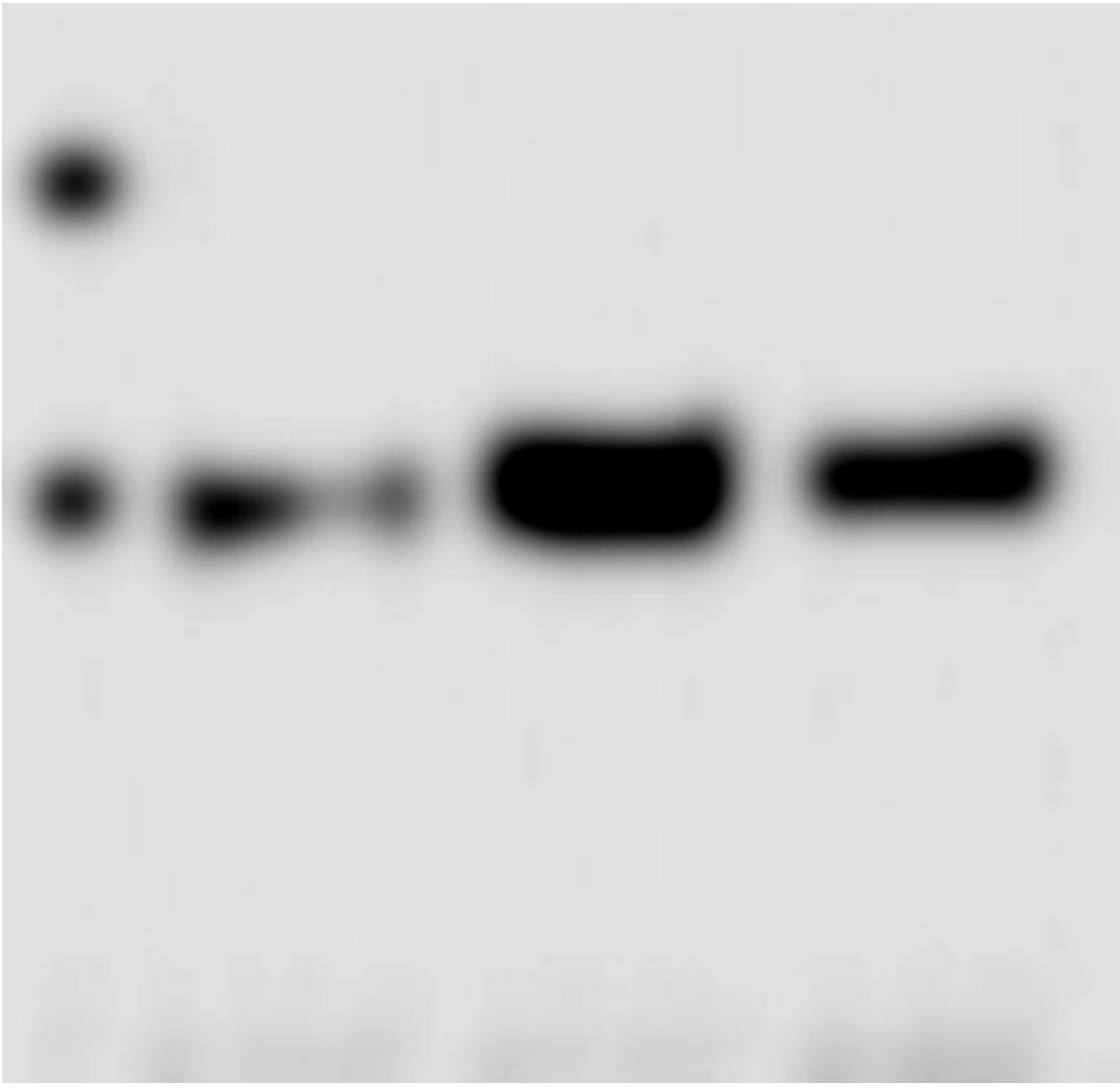

fig4c MAFbx.jpg

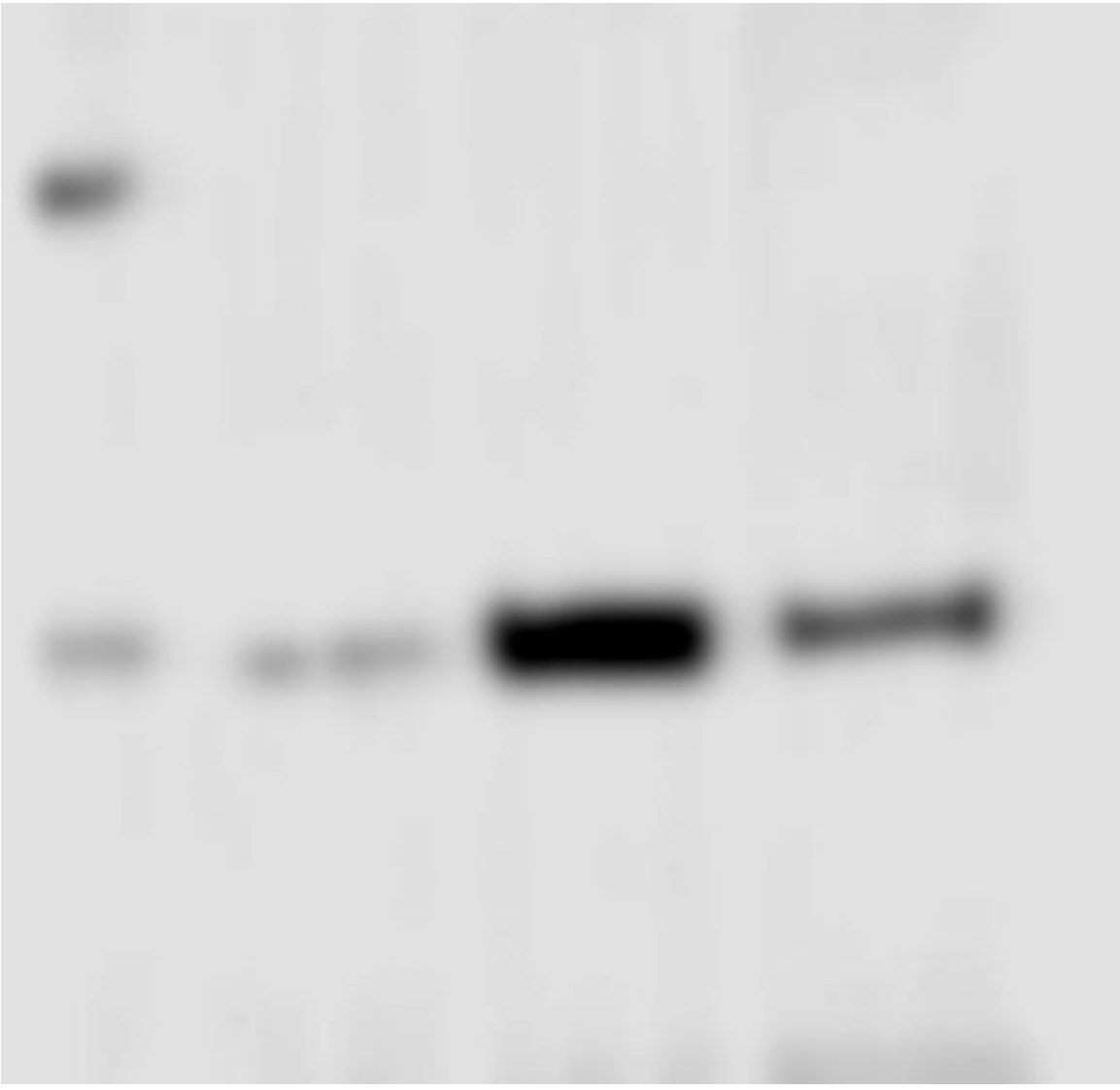

fig4c MuRF1.tif

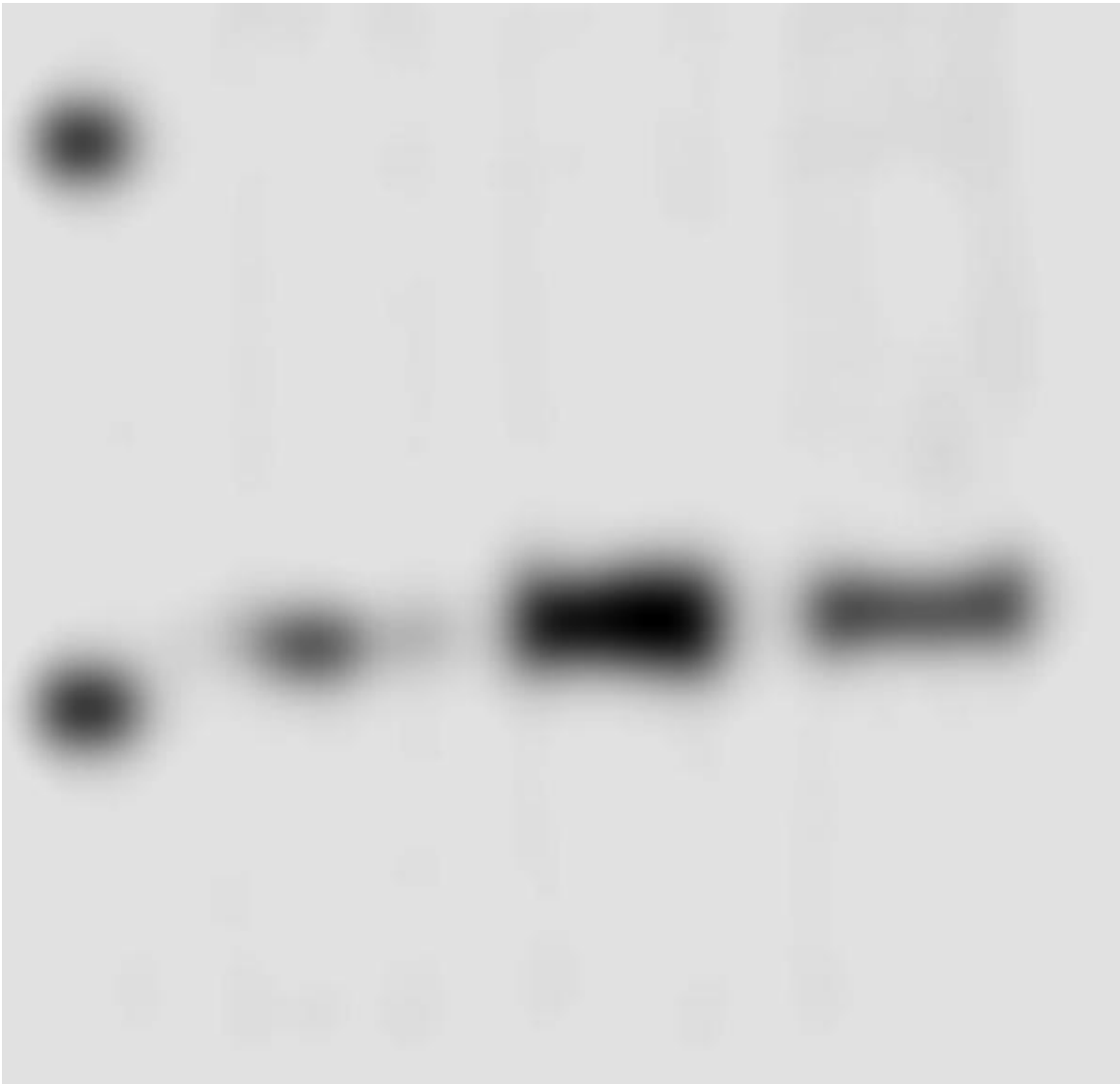

fig4c myostatin .jpg

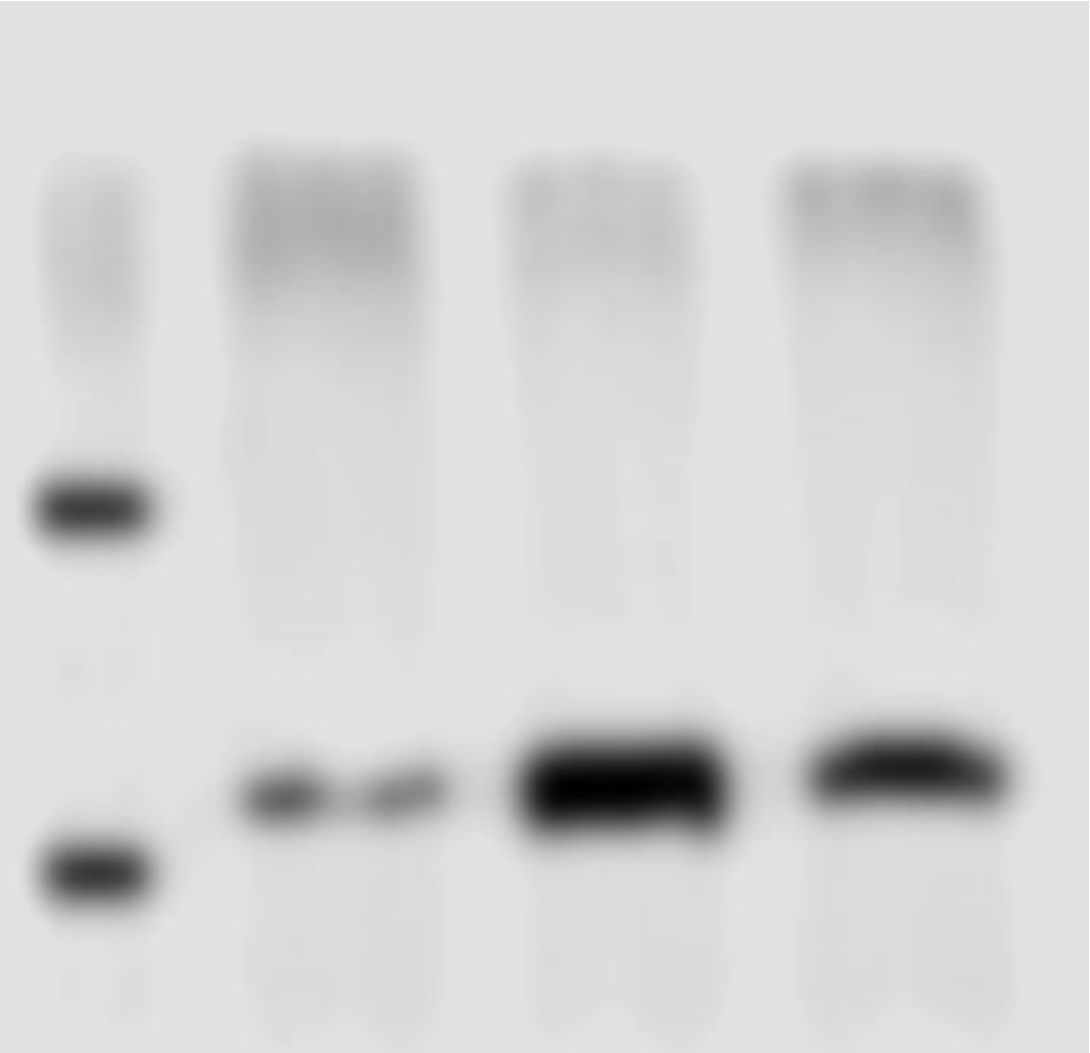

fig4c PKCλ

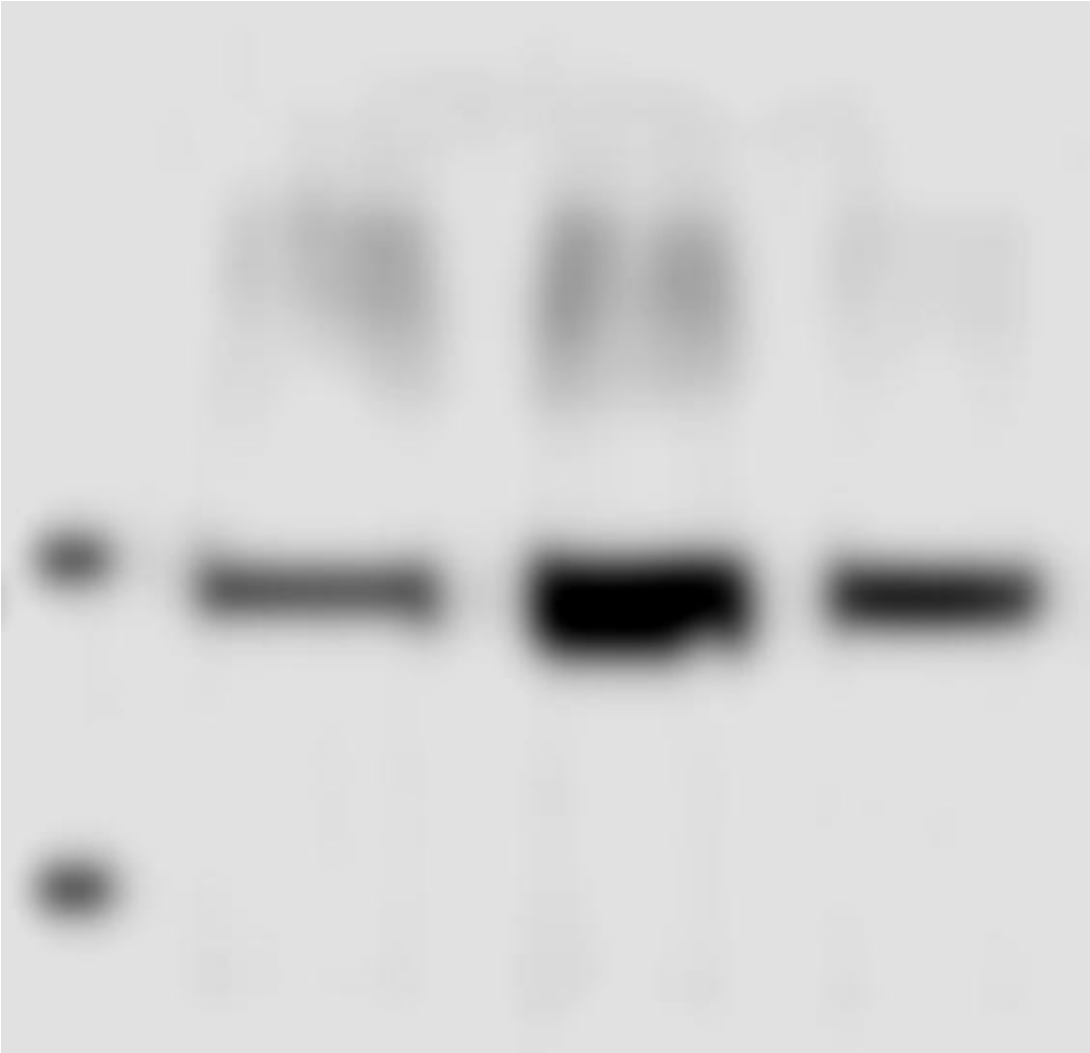

fig4c P-PKCλ

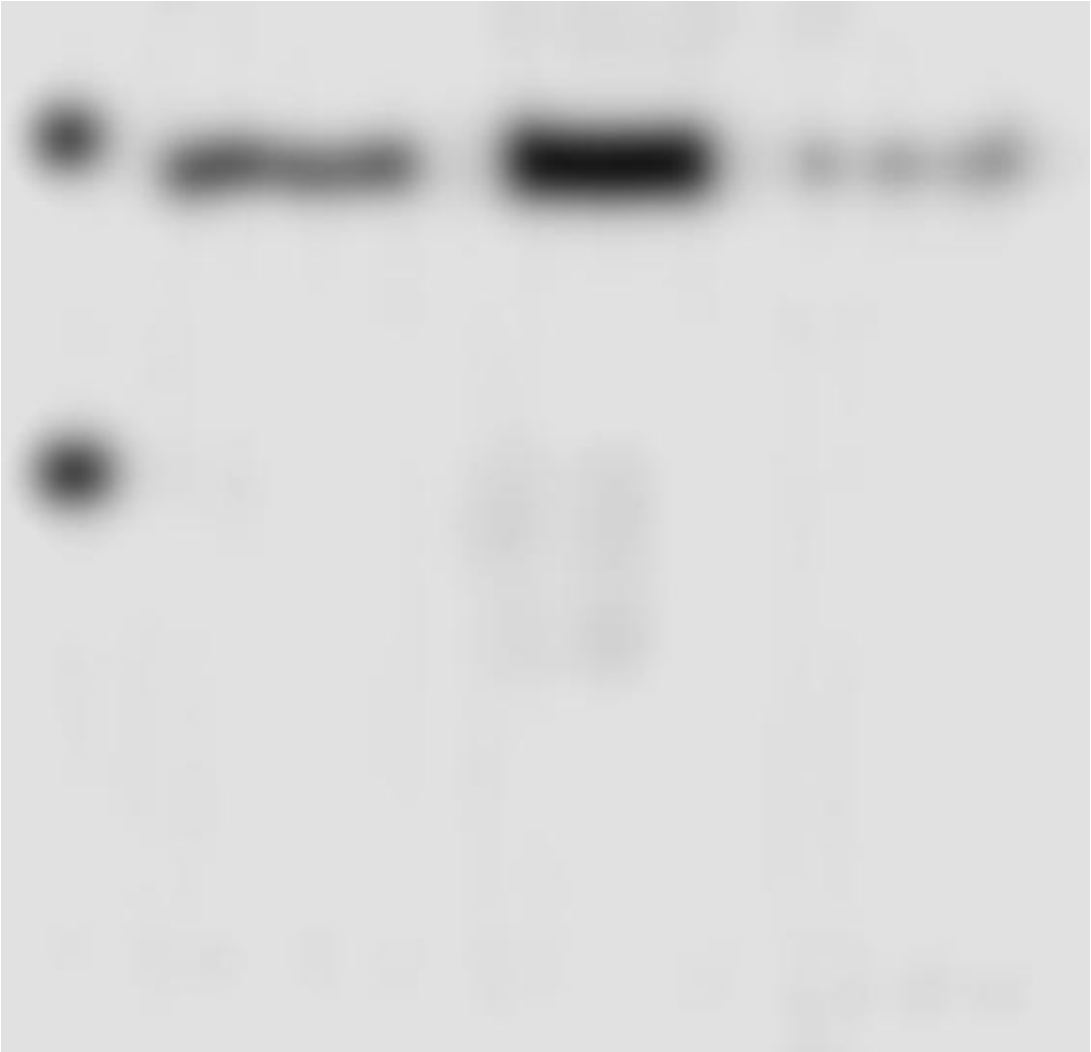

fig4c PSTAT3.jpg

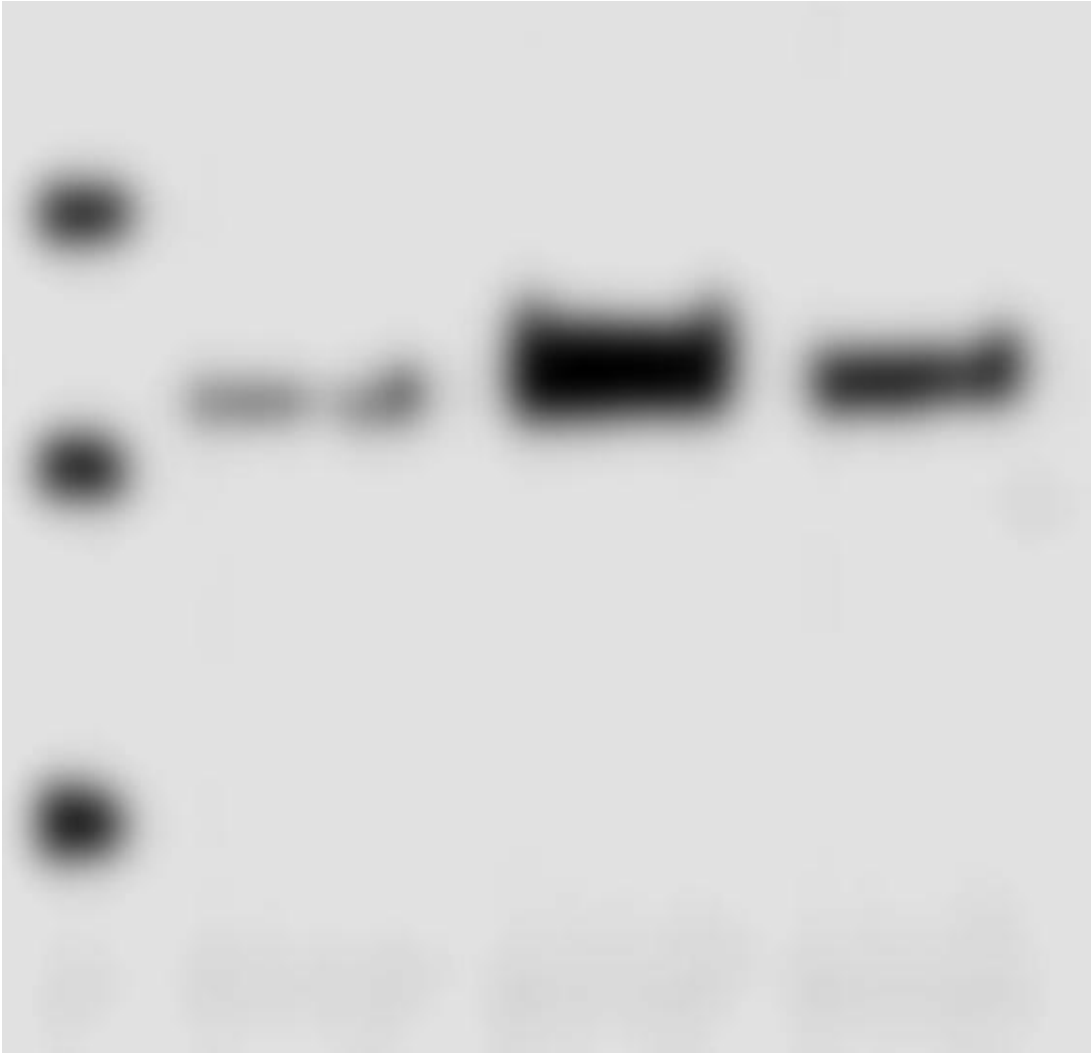

fig4c STAT3.jpg

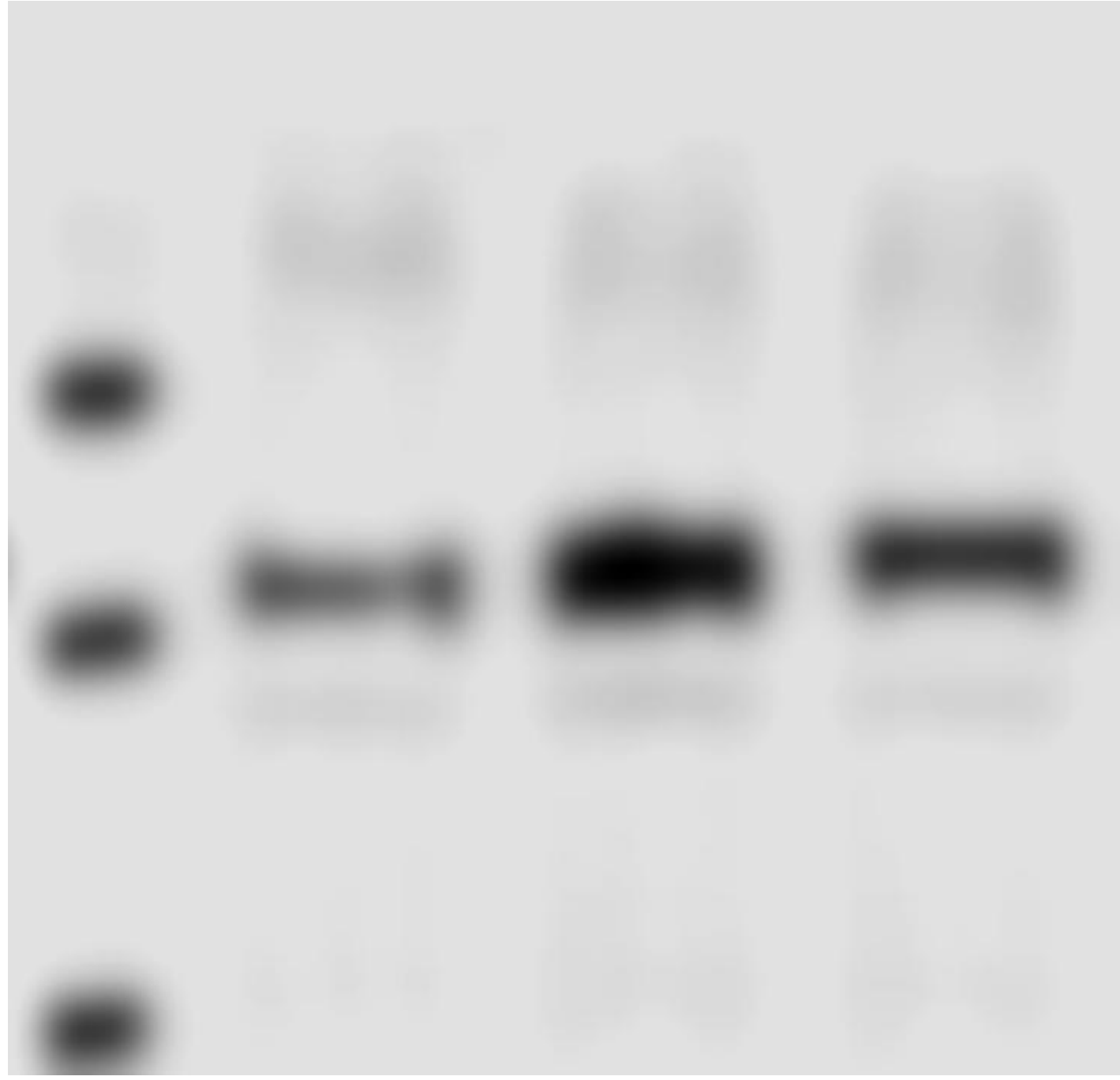

SF1A PKC-λ

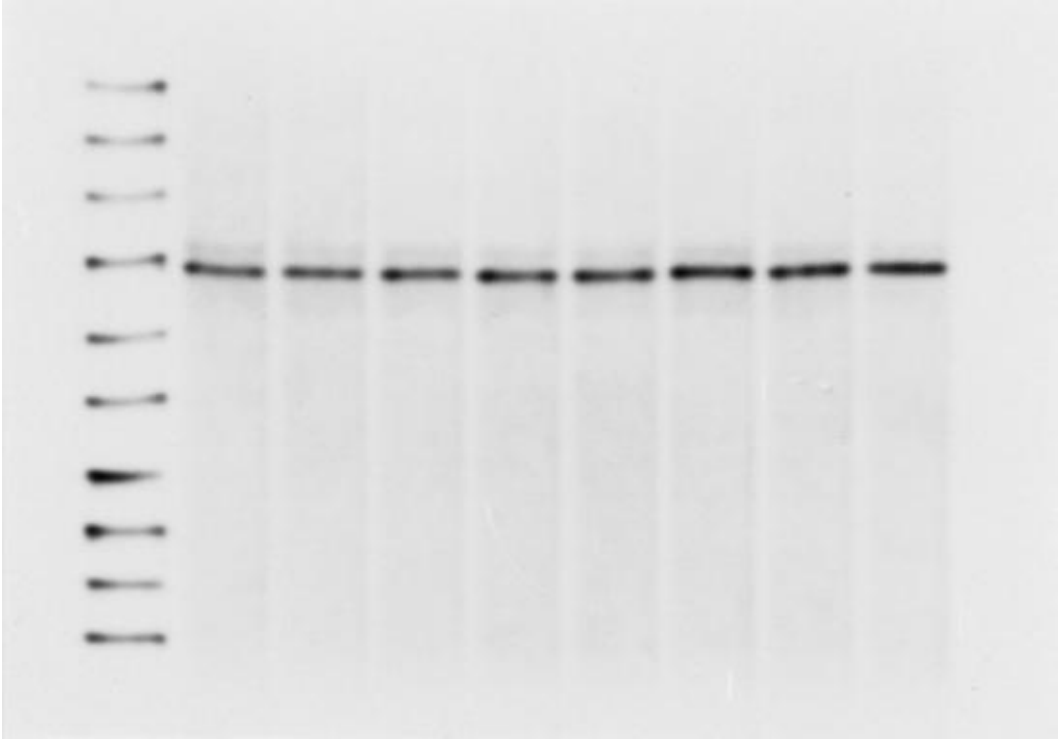

SF1A p-PKCλ

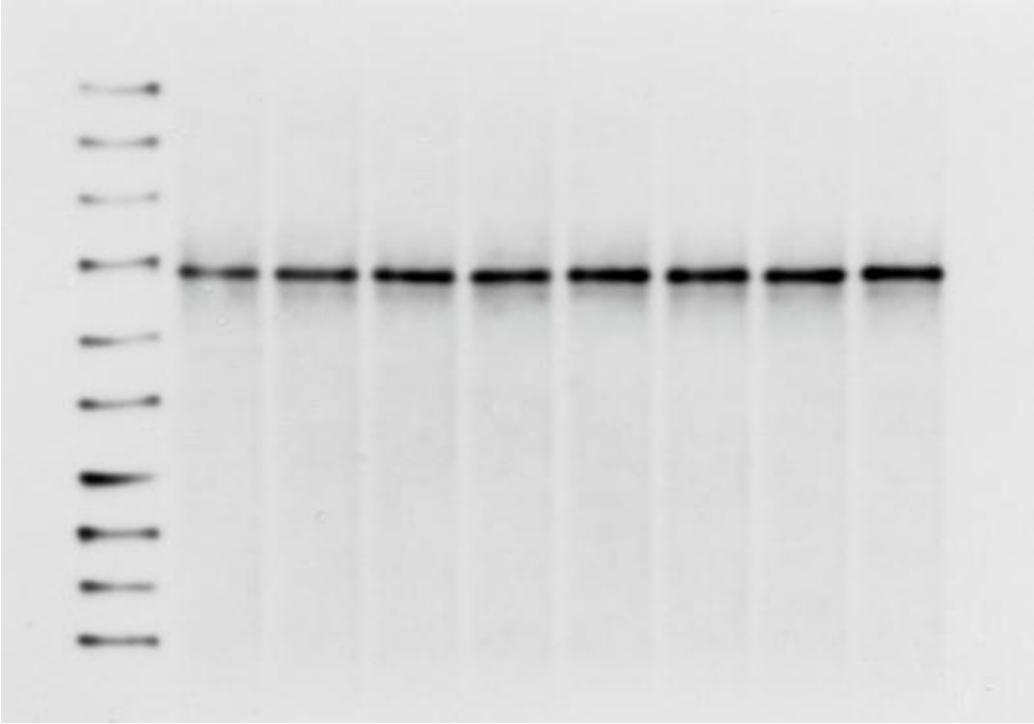

SF1A tubulin

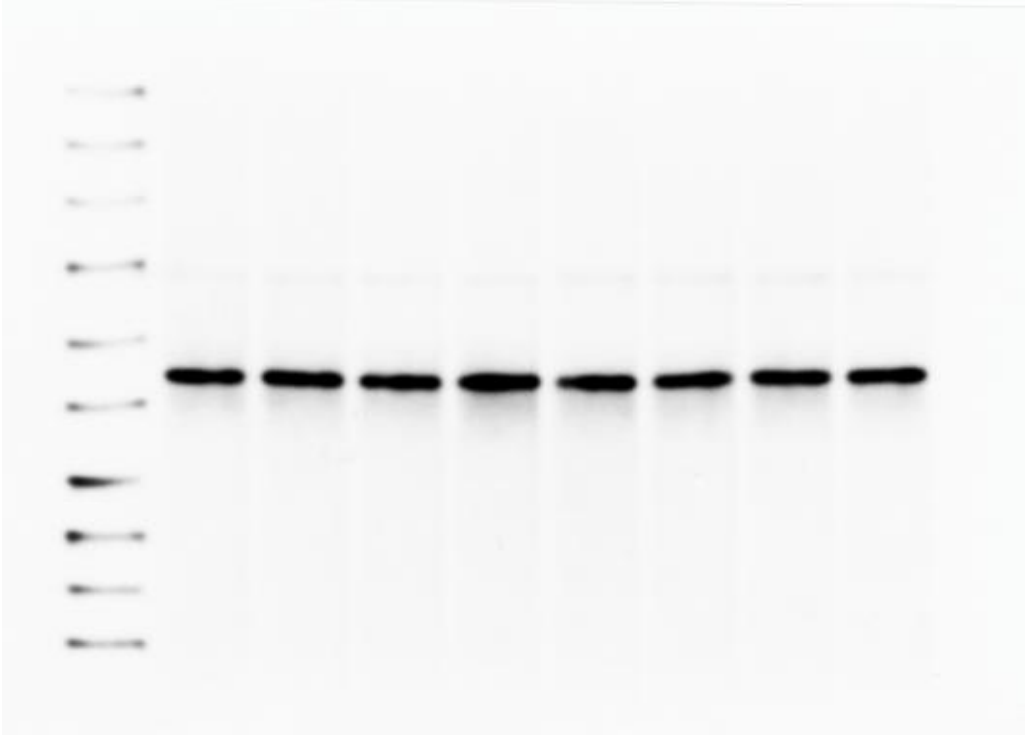

SF1B PKC-λ

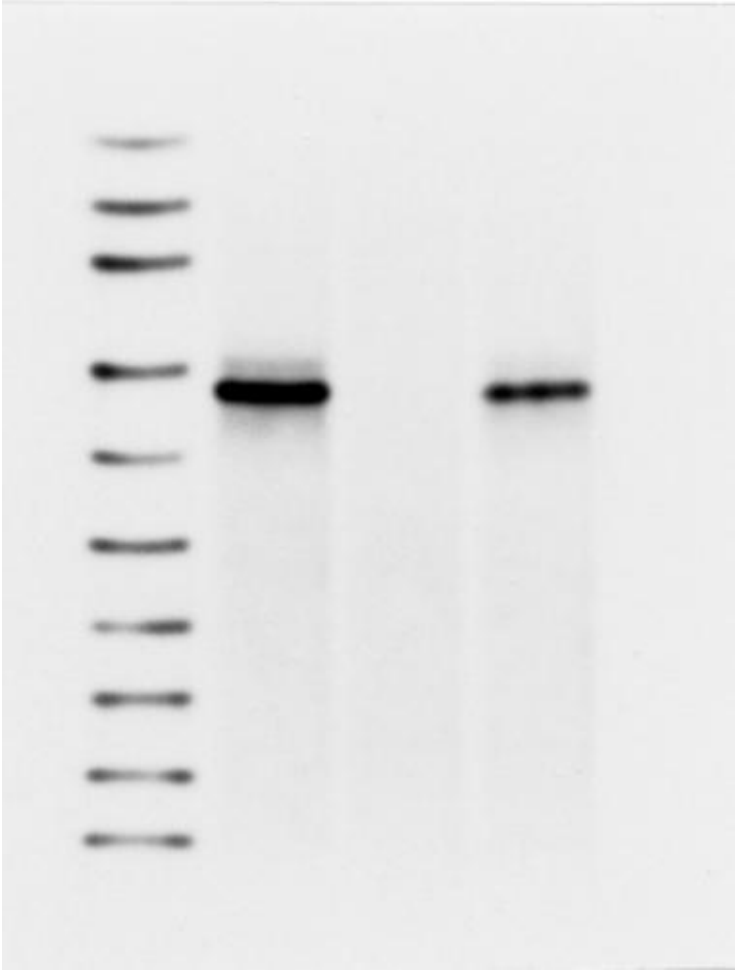

SF1B STAT3

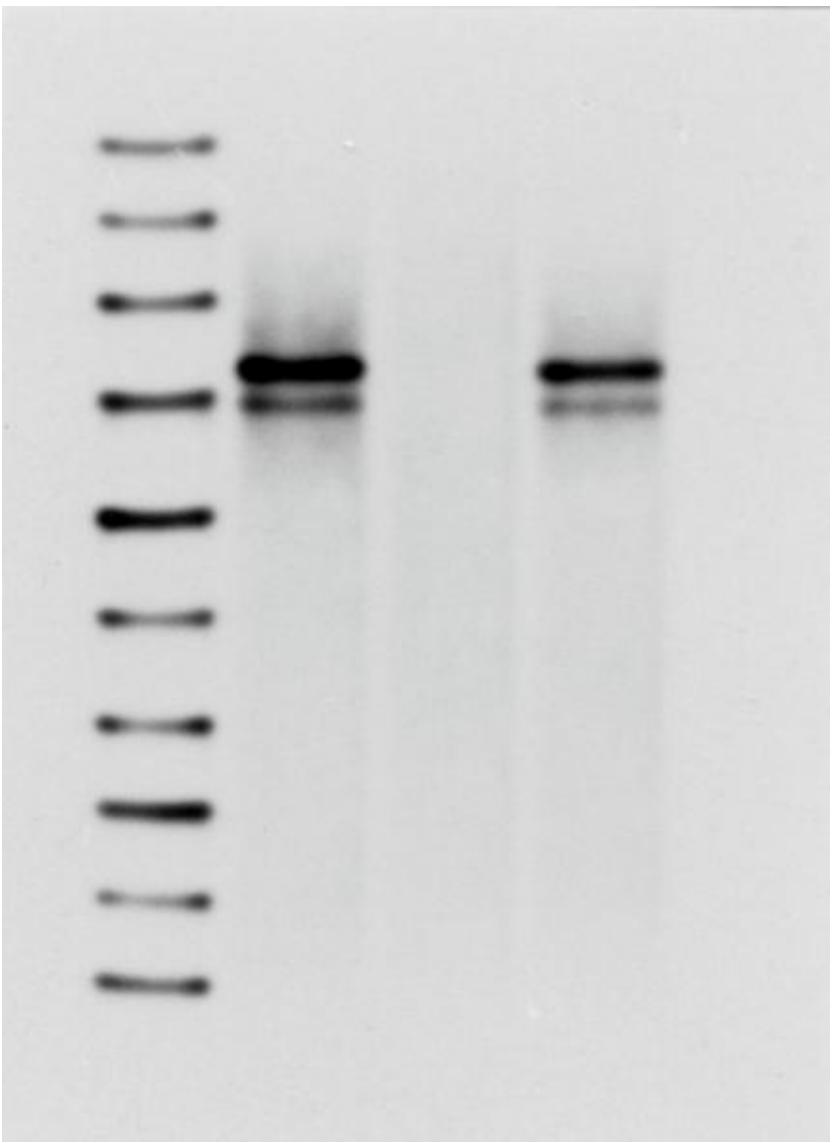

SF1C B-tubulin

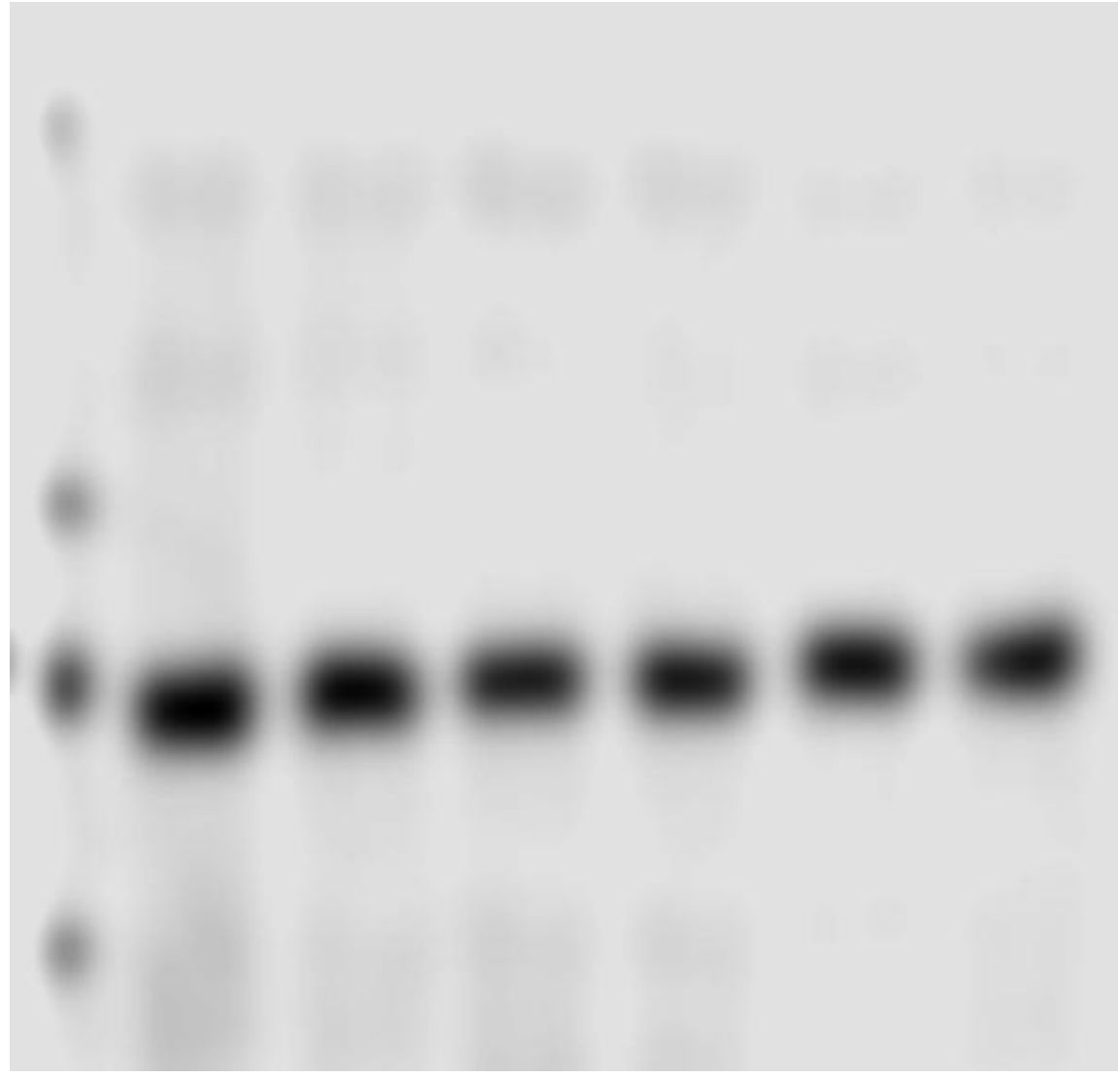

SF1C PKCλ.tif

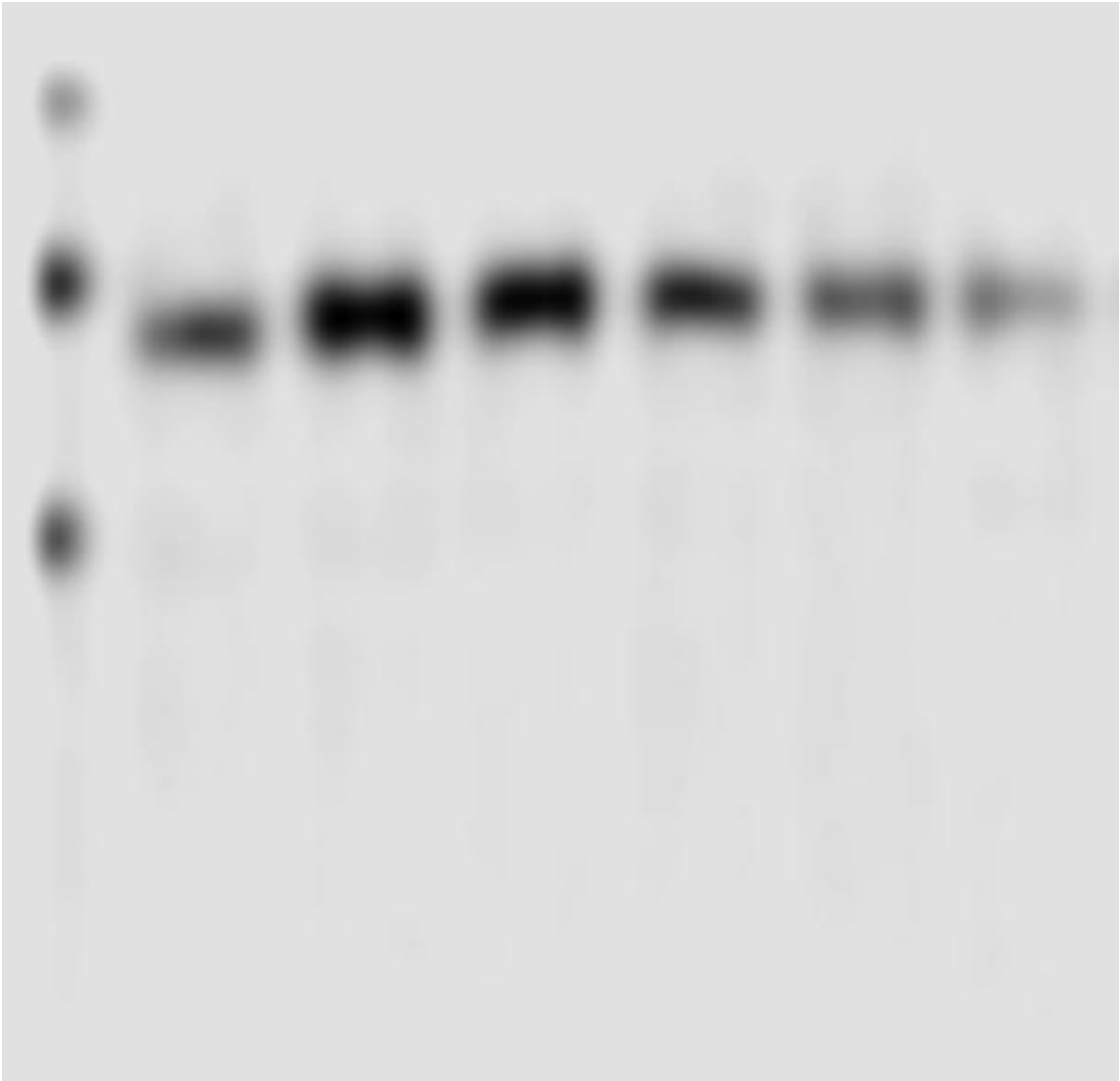

SF1C p-PKCλ

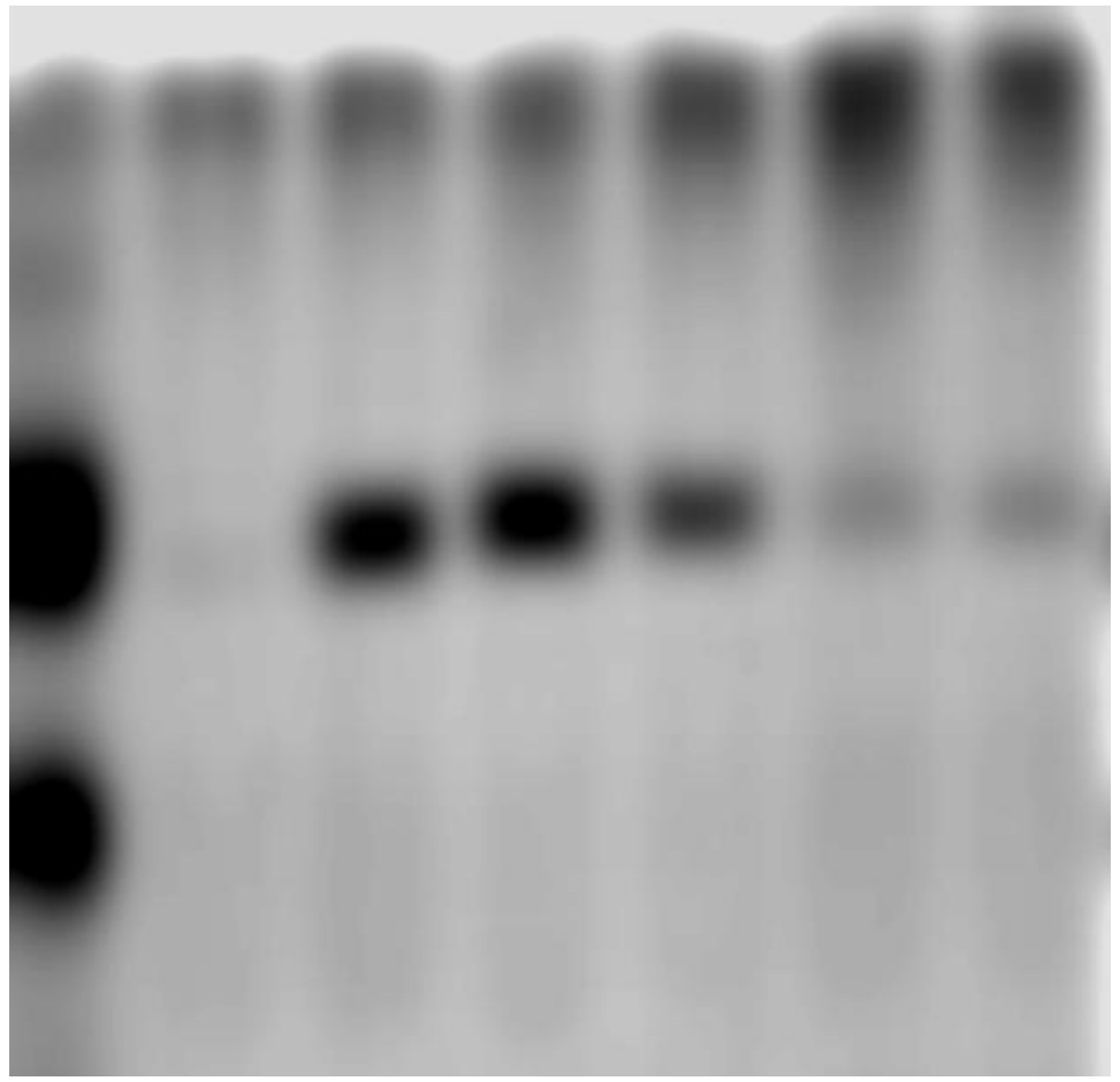

SF1D B-tubulin

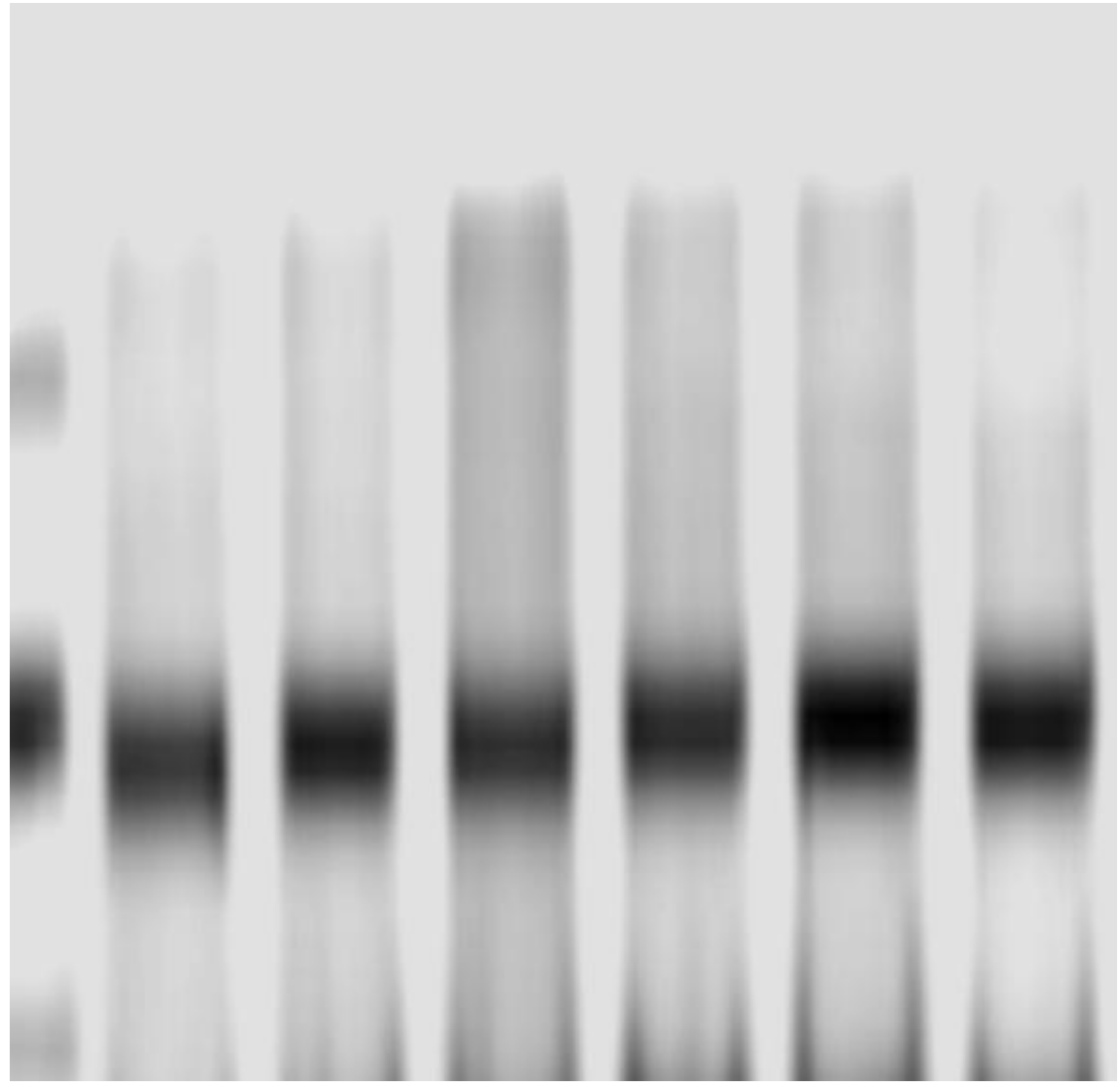

SF1D PKCλ

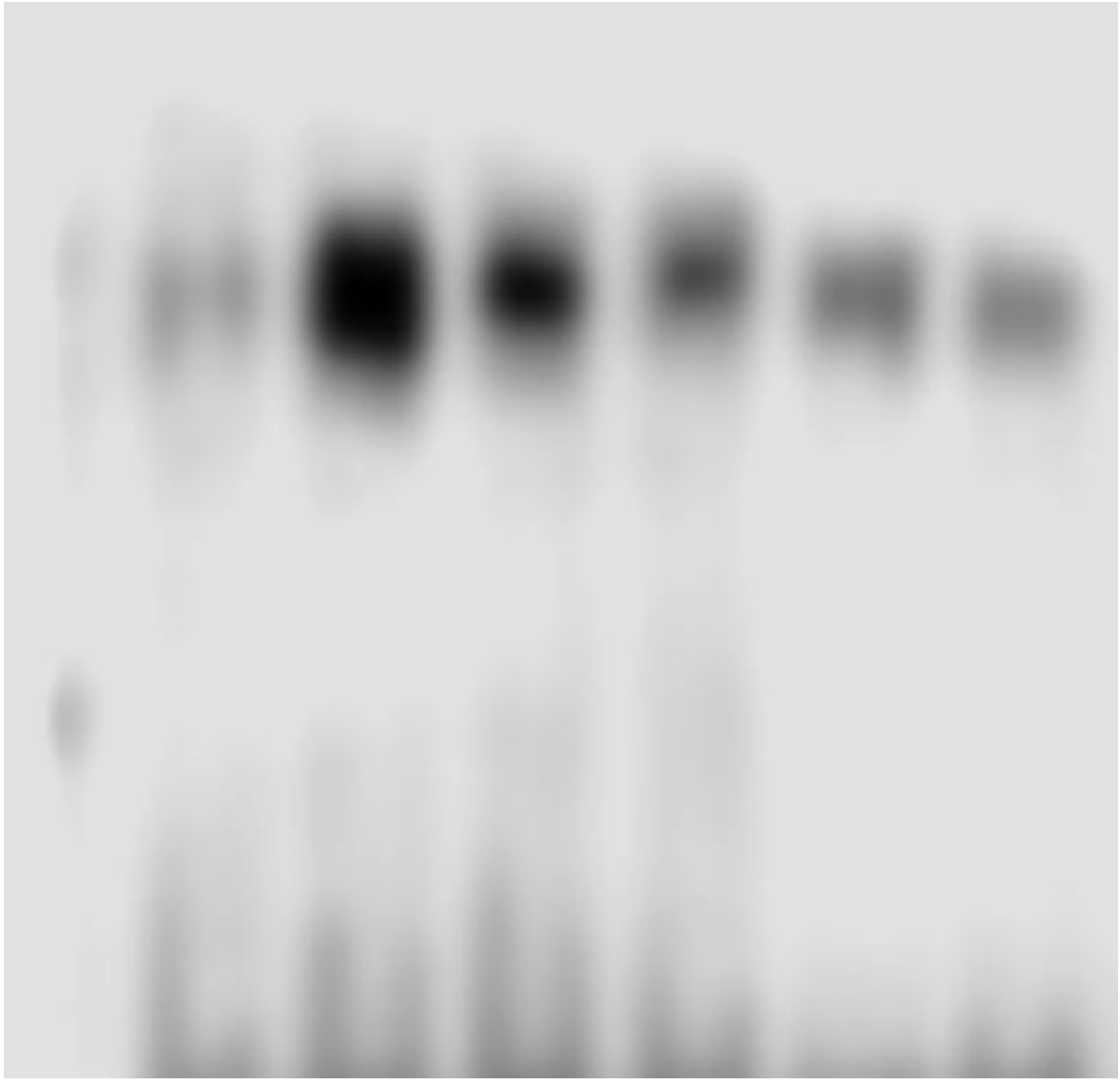

SF1D P-PKCλ

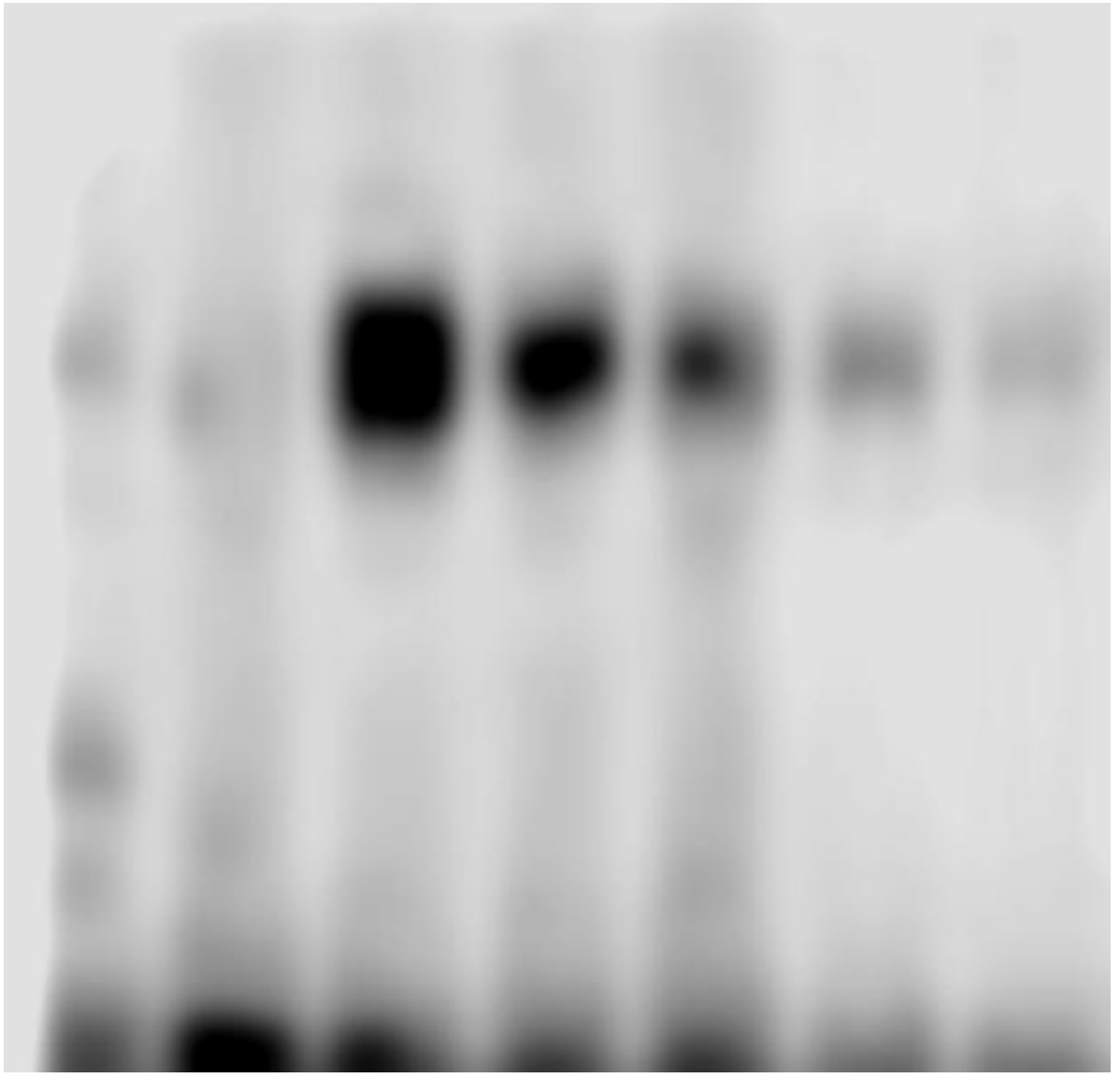

Supplement: Supplementary file 1 [file Image1.pdf]
